# 1,3‐Diketone‐Modified Nucleotides and DNA for Cross‐Linking with Arginine‐Containing Peptides and Proteins

**DOI:** 10.1002/anie.202105126

**Published:** 2021-07-02

**Authors:** Denise‐Liu' Leone, Martin Hubálek, Radek Pohl, Veronika Sýkorová, Michal Hocek

**Affiliations:** ^1^ Institute of Organic Chemistry and Biochemistry Czech Academy of Sciences Flemingovo nam. 2 16610 Prague 6 Czech Republic; ^2^ Department of Organic Chemistry Faculty of Science Charles University in Prague Hlavova 8 12843 Prague 2 Czech Republic

**Keywords:** bioconjugations, cross-linking reactions, DNA polymerases, nucleotides, proteins

## Abstract

Linear or branched 1,3‐diketone‐linked thymidine 5′‐*O*‐mono‐ and triphosphate were synthesized through CuAAC click reaction of diketone‐alkynes with 5‐azidomethyl‐dUMP or ‐dUTP. The triphosphates were good substrates for KOD XL DNA polymerase in primer extension synthesis of modified DNA. The nucleotide bearing linear 3,5‐dioxohexyl group (HDO) efficiently reacted with arginine‐containing peptides to form stable pyrimidine‐linked conjugates, whereas the branched 2‐acetyl‐3‐oxo‐butyl (PDO) group was not reactive. Reaction with Lys or a terminal amino group formed enamine adducts that were prone to hydrolysis. This reactive HDO modification in DNA was used for bioconjugations and cross‐linking with Arg‐containing peptides or proteins (e.g. histones).

## Graphical abstract

Arginine‐specific 1,3‐diketone‐linked reactive nucleotides are reported for the enzymatic construction of DNA probes and cross‐linking with peptides and proteins.

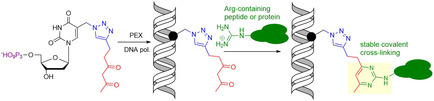

Protein‐DNA interactions are important in many biological processes[^1^, ^2^] and therefore identification of these interactions is urgently needed.^[3]^ Covalent cross‐linking of the binding proteins with reactive DNA, mostly driven by proximity effect, is one of the most promising methods for their studies. In addition, covalent protein‐DNA conjugates are also useful for other applications in chemical biology, biosensing^[4]^ or targeting of therapeutic nucleic acids^[5]^ and therefore new efficient bioconjugation methods are highly desirable.^[6]^ There are some widely‐used non‐specific cross‐linking methods, typically based on photochemical generation of reactive species, that is, radicals^[7]^ or carbenes^[8]^ in DNA that cross‐link randomly to any amino acids. More useful but also challenging are amino acid‐specific cross‐linking reactions and there were only handful of them reported for DNA‐protein cross‐linking. DNA bearing free thiol group cross‐linked with Cys through disulfide formation.^[9]^ Vinylsulfonamide group was also used to cross‐link with Cys through conjugate addition,^[10]^ whereas chloroacetamide alkylated Cys or His in proteins.^[11]^ Most commonly used was cross‐linking of aldehyde‐linked DNA with Lys either through reversible imine formation^[12]^ or through irreversible reductive amination[^13^, ^14^] that requires additional NaBH_3_CN as stoichiometric reductant. Recently we have reported squaramate‐linked DNA that reacted with Lys‐containing peptides and proteins without any additional reagent.^[15]^ As most of these reactive groups are not compatible with phosphoramidite synthesis, the polymerase synthesis using reactive‐labelled 2′‐deoxyribonucleoside triphosphates (dNTPs) is advantageously used for the construction of the reactive DNA probes.^[16]^

Arginine is another relevant nucleophilic amino acid often present in DNA‐binding proteins.^[17]^ To the best of our knowledge, there has been only one example of Arg targeting with DNA using 4,6‐dioxoheptylamido group attached to DNA postsynthetically to a 2′‐amino group of an 2′‐amino‐2′‐deoxyuridine which was chemically incorporated to the oligonucleotide (ON) through phosphoramidite chemistry.^[18]^ In affinity labelling and bioconjugations, the most common reactive groups used to target Arg are glyoxals^[19]^ and other 1,2‐dioxo derivatives.^[20]^ Since these highly reactive species were difficult to attach to dNTPs for polymerase synthesis of reactive DNA, we focused on 1,3‐diketones and report here the synthesis of dNTP building blocks bearing 2,4‐pentandione (either linear linked to C1 or branched linked through C3), their use in the enzymatic synthesis of reactive DNA probes and comparison of reactivity with Arg‐containing peptides and proteins.

We designed and synthesized modified thymidine nucleotides bearing the 1,3‐diketone attached at position 5 to ensure that the modification points out to the major groove of DNA and that the nucleotides can be substrates for DNA polymerases. We chose pentan‐2,4‐dione as the suitable 1,3‐diketone moiety and attached it to the nucleotide either through the terminal or internal carbon and planned to attached them through the Cu‐catalyzed alkyne‐azide cycloaddition (CuAAC)^[21]^ to 5‐azidomethyl‐2′‐deoxyuridine nucleotides, that are easily available through radical bromination of protected thymidine, azidation and phosphorylation.^[22]^ The building blocks were the corresponding terminal alkynes and oct‐7‐yne‐2,4‐dione (**1**) and 3‐(prop‐2‐yn‐1‐yl) pentane‐2,4‐dione (**2**) that were easily prepared according to published procedures.^[23]^ The CuAAC click reaction of alkynes **1** or **2** with azido‐linked thymidine monophosphate **dT^N3^MP** or triphosphate **dT^N3^TP** proceeded smoothly (Scheme 1) to give the desired products **dT^HDO^MP** (40 % yield), **dT^PDO^MP** (26 % yield), **dT^HDO^TP** (18 % yield), **dT^PDO^TP** (34 % yield). The isolated yields were lowered by difficult purification of the nucleotides and partial hydrolysis of the triphosphates, but the procedure was straightforward and enabled the preparation of sufficient quantities of the modified nucleotides. The modified **dT^X^MP** were then tested in model reactions with *N*‐BocArginine or Arg‐containing tripeptide (3 equiv). The reactions proceeded in NaHCO_3_/Na_2_CO_3_ buffer (0.5 M, pH 10) at 25 °C for 24 h. While the linearly‐linked **dT^HDO^MP** reacted well to give the corresponding conjugates **dT^HDOArg^MP** (42 % yield) or **dT^HDOARA^MP** (35 % yield), the branched **dT^PDO^MP** did not give any reaction even with large excess of the Arg or peptide (20 equiv). The conjugates **dT^HDOArg^MP** and **dT^HDOARA^MP** were isolated by reverse phase HPLC in pure form and fully characterized by NMR and MS to confirm the covalent binding and formation of the stable pyrimidine ring from the guanidine group and diketone. They did not undergo any hydrolysis in water at r.t. in 20 h (Figure S45 SI).

**Scheme 1:**
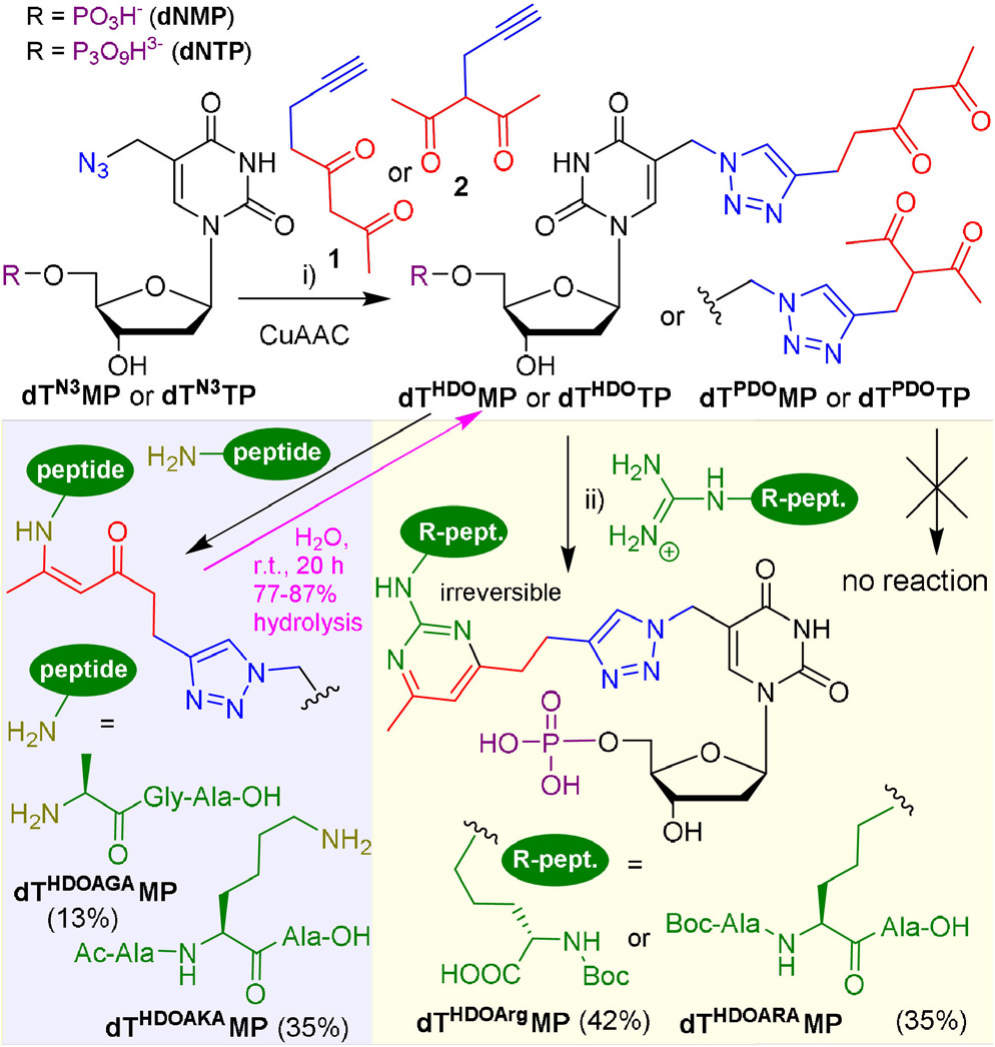
Synthesis of HDO‐ and PDO‐modified dNMPs and dNTPs and reactions with Arg and Arg‐ or Lys‐containing peptides. Conditions: NaHCO_3_/Na_2_CO_3_ buffer (0.5 M, pH 10), 25 °C, 24 h.

The reactions of **dT^HDO^MP** with excess of N‐terminal amino group of AGA tripeptide or with Lys‐containing tripeptide gave enamine adducts **dT^HDOAGA^MP** (13 %) and **dT^HDOAKA^MP** (35 %), that were prone to hydrolysis in water (77–87 % at r.t. in 20 h).

Then we tested the modified triphosphates **dT^HDO^TP** and **dT^PDO^TP** as substrates for KOD XL DNA polymerase in primer extension experiments (PEX) (Figure 1 a). Both of them were good substrates and gave full‐length 19‐ or 30‐mer PEX products containing 1, 2 or 4 modifications as demonstrated by PAGE analysis (Figure 1 e, for the ON sequences see Table S1 and for gels with 2 or 4 modifications, see Figure S1 in SI) and all the modified DNAs were also characterized by MALDI‐TOF analysis (Table S3 and Figures S20–S27 in SI).

**Figure 1:**
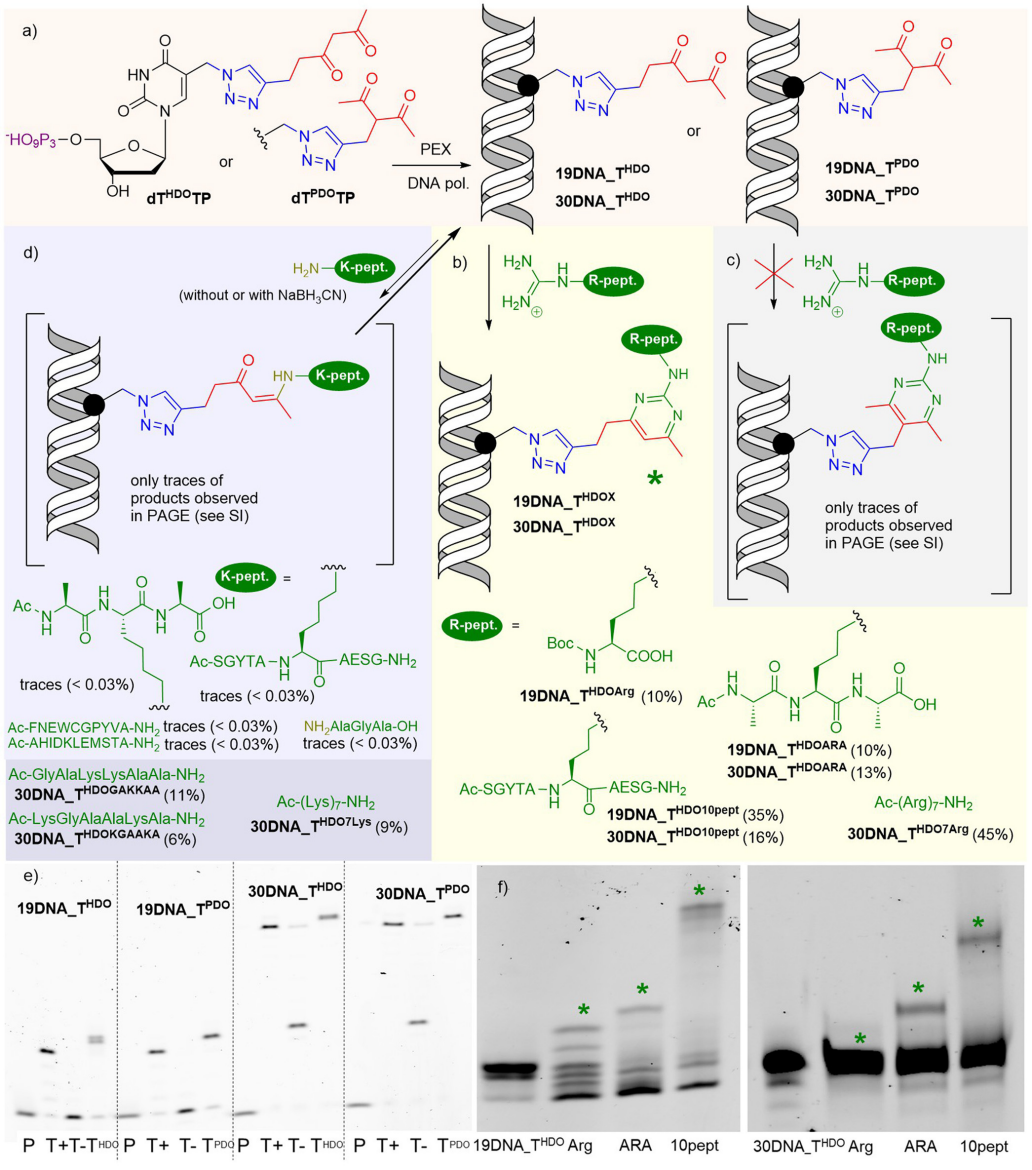
a) Enzymatic synthesis of **19/30DNA_T^HDO^** and **19/30DNA_T^PDO^**. b) Cross‐linking of **19/30DNA_T^HDO^** with Arg‐containing tri‐, hepta‐ and decapeptide c) Cross‐linking of **30DNA_T^PDO^** with Arg‐containing tri‐, hepta‐ and decapeptide d) Cross‐linking of **30DNA_T^HDO^** with Lys‐containing or N‐unprotected or negative control peptides. e) PAGE analysis of the PEX reactions with **dT^HDO^TP** or **dT^PDO^TP** using KOD XL DNA polymerase, primer prim^A^ and templates Temp^19_1T^ or Temp^30_1T^. P: primer, (T+): Natural dNTPs,(T‐):natural dNTPs without dTTP,(**T^HDO/PDO^**): **dT^HDO^TP** or **dT^PDO^TP**, dCTP,dGTP, dATP;. f) PAGE analysis of cross‐linking of **19DNA_T^HDO^** and **30DNA_T^HDO^** with N‐BocArg, and arginine containing tri‐ and decapeptide (SI, part 2.3).

Next we used 19‐bp or 30‐bp double‐stranded DNAs (**19DNA_T^HDO^**, **19DNA_T^PDO^**, **30DNA_T^HDO^** and **30DNA_T^PDO^**) containing one modification to test cross‐linking reaction with N‐BocArg and Ac‐Arg‐containing tripeptide and decapeptide (Figure 1 b,c). The reactions were conducted in presence of an excess of amino acid or peptide (10^6^ equiv) and at 37 °C for 18 h. Similarly to the reactions of model nucleotides, DNA bearing branched PDO gave only traces of conjugated products on PAGE analysis (Figures S4–S7 in SI), whereas the modified DNA bearing linear HDO group reacted well as shown in the PAGE analysis (Figure 1 f) confirming the formation of the desired cross‐linked conjugates with slower mobility in conversions 10–35 %: **19DNA_T^HDOArg^** (10 %), **19DNA_T^HDOARA^** (10 %), **19DNA_T^HDO10pept^** (35 %), **30DNA_T^HDOARA^** (13 %) and **30DNA_T^HDO10pept^** (16 %). Only for **30DNA_T^HDOArg^** no difference in mobility was observed in the PAGE analysis due to minute difference in size and resolution limit of the 20 % PAGE gel. Most of the products of conjugation were also successfully characterized by MALDI (Table S4 in SI).

Furthermore, we wanted to test the reactivity of **DNA_T^HDO^** toward Lys‐containing peptides which could form reversible enamine‐bonds under the reaction conditions (Figure 1 d). The reactions of **30DNA_T^HDO^** and Lys‐containing peptides, with N‐unprotected tripeptide and with two negative control peptides containing all amino acids except for Arg were conducted using the same conditions as for the arginine derivatives either in absence or in the presence of NaBH_3_CN. In all cases we have observed only traces (<0.03 %) of conjugation products with the exception of the reactions with peptides containing two or more Lys that gave 6–11 % conversion probably due to formation of double enamine adducts that were more stable toward hydrolysis (Table S5 in SI). These experiments clearly show the HDO group reacts with Lys or terminal NH_2_ only reversibly and the enamine adducts are unstable under denaturating or hydrolysis conditions.

Then we approached the ultimate goal of this study, to test the reactivity of the HDO‐modified DNA (**19DNA_T^HDO^** and **30DNA_T^HDO^**) with proteins. Following our previous work,^[15]^ we used bovine serum albumin (BSA) as a negative control of a protein containing 26 Arg which does not interact with DNA, GST‐tagged core domain of p53 protein (GSTp53CD)^[24]^ as an example of a DNA‐binding protein containing arginine but not in the proximity to the modification in the binding site,^[25]^ and finally a set of Arg‐rich histones (H2A, H2B, H3.1 and H4) that strongly bind DNA and their arginines participate on the interaction with DNA. Unlike the model‐studies with peptides (that required a large excess of Arg‐containing peptides to observe any cross‐linking), the cross‐linking reactions were performed using 5 equiv or even 1 equiv (SI, Figure S11 and S14) of the corresponding proteins. A simple kinetic study of the reaction of **30DNA_T^HDO^** with histone H4 showed that the maximum conversion is reached within 7–23 h whereas longer times lead to significant decomposition (SI, Figure S17). Therefore the reactions were performed for 18 h either in NaHCO_3_ buffer (pH 10, as used for the model‐studies) or in more physiologically relevant NaHCO_3_ (pH 8.5) or KHCO_3_/HEPES (pH 8.5) buffers. In all cases denaturing SDS‐PAGE analysis confirmed the formation of covalent adducts of **19DNA_T^HDO^** or **30DNA_T^HDO^** with the histones in conversions of 24–35 % (for 5 equiv of protein) or 12–27 % (for 1 equiv of protein) (Figure 2 b, Figure S11, S14, S15 in SI). The identity of the covalent DNA‐protein conjugates with H2A, H2B and H4 was also confirmed by SDS‐PAGE with protein staining (PageBlue^TM^) and by HPLC‐MS analysis using electrospray ionization^[26]^ (Figures S33–35 in SI). The irreversibility of the cross‐link was proved by successive reaction with hydroxylamine which did not cleave the cross‐linked products (Figure S13 in SI).^[18]^ On the other hand, no cross‐linked conjugates were observed in reactions with BSA or GSTp53CD and only traces of products were observed in the case of cross‐linking reactions of branched **30DNA_T^PDO^** with histones (Figure S16 and S19 in SI).

**Figure 2:**
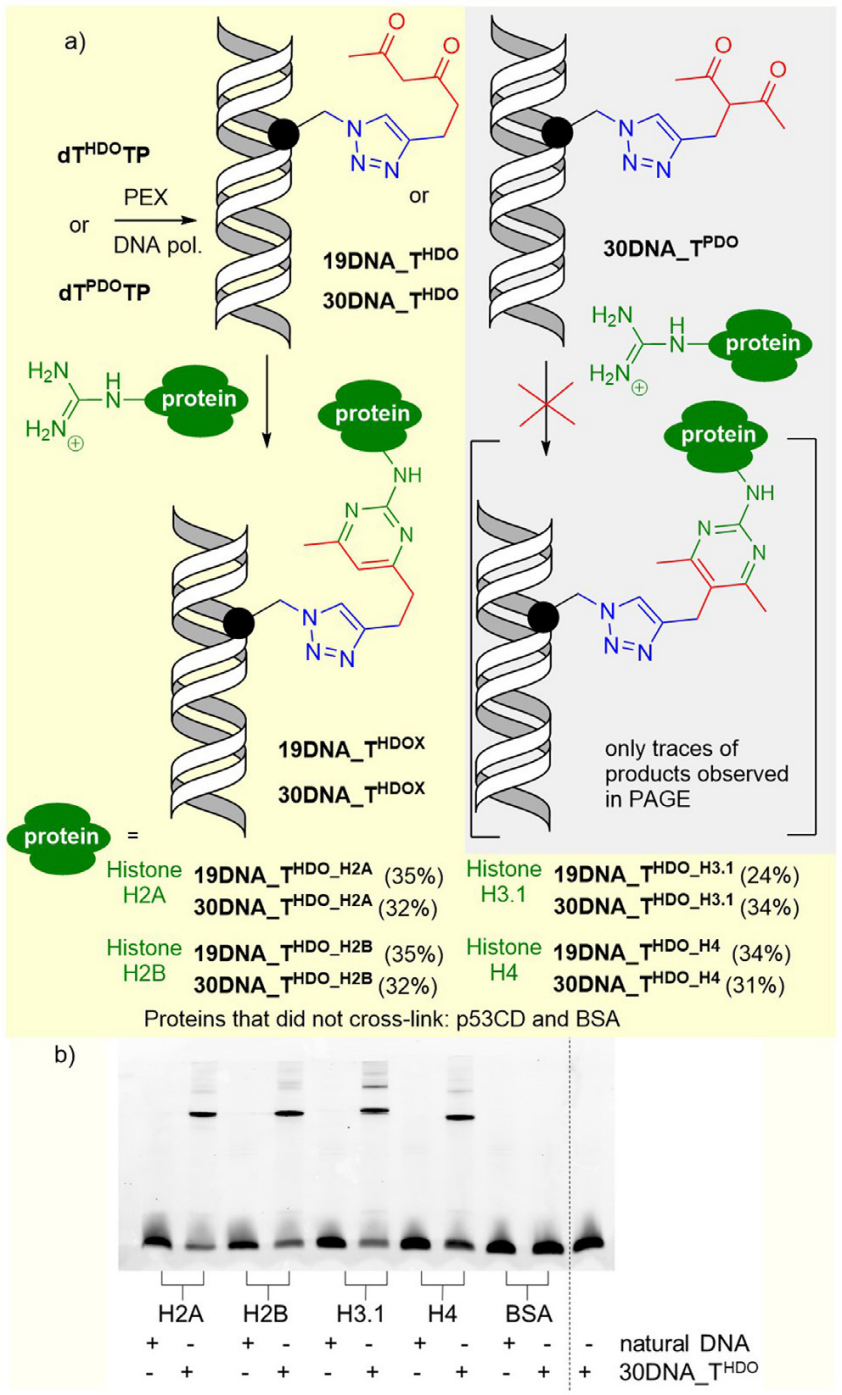
Cross‐linking of 1,3‐diketone‐modified DNA with proteins: a) reaction scheme. Conditions: NaHCO_3_/Na_2_CO_3_ buffer (0.05 M, pH 10), 37 °C, 18 h: b) 17.5 % SDS‐PAGE analysis of cross‐linking of **30_DNA_T^HDO^** with histones (5 equiv of protein) and BSA.

In conclusion we designed and synthesized novel 1,3‐diketone‐linked dNTPs and showed that they are good substrates for KOD XL polymerase in PEX reactions to construct reactive DNA probes. The HDO moiety reacts with arginine to form a stable aromatic pyrimidine ring and the covalent adducts are stable toward hydrolysis. The reaction of **DNA_T^HDO^** with Arg‐containing peptides proceeded only in presence of large excess of the peptide, whereas the reactions with Lys‐containing peptides gave mostly just traces of products because the enamine‐adducts were unstable and prone to hydrolysis. The reactivity of DNA bearing the branched PDO group (**DNA_T^PDO^**) with Arg‐containing peptides was much lower giving only traces of the conjugation products. Reactions of **DNA_T^HDO^** with Arg‐containing DNA‐binding proteins (histones) proceeded in good conversions even in 1:5 or 1:1 ratio, due to the proximity effect. Thus we have complemented the toolbox of reactive substituents for DNA cross‐linking with a new Arg‐specific reactive group. The approach and nucleotide building blocks can be now applied in construction of reactive DNA probes for cross‐linking to Arg‐containing DNA‐binding proteins, for synthesis of stable DNA‐peptide or DNA‐protein conjugates,[^4^, ^5^] as well as for post‐synthetic labelling of DNA.^[6]^

## Conflict of interest

The authors declare no conflict of interest.

## Supporting information

As a service to our authors and readers, this journal provides supporting information supplied by the authors. Such materials are peer reviewed and may be re‐organized for online delivery, but are not copy‐edited or typeset. Technical support issues arising from supporting information (other than missing files) should be addressed to the authors.

Supporting Information
