# Supplementary material for: 1,3‐Diketone‐Modified Nucleotides and DNA for Cross‐Linking with Arginine‐Containing Peptides and Proteins
Source: Angew Chem Int Ed Engl. 2021 Jul 2;60(32):17383–7. doi: 10.1002/anie.202105126 (PMC8362068; doi:10.1002/anie.202105126)
Supplement: Supplementary file 1 — Supporting Information [file ANIE-60-17383-s001.pdf]

Angewandte Chemie
Eine Zeitschrift der Gesellschaft Deutscher Chemiker

Supporting Information

1,3-Diketone-Modified Nucleotides and DNA for Cross-Linking with Arginine-Containing Peptides and Proteins

*Denise-Liu' Leone, Martin Hubálek, Radek Pohl, Veronika Sýkorová, and Michal Hocek**

anie_202105126_sm_miscellaneous_information.pdf

Table of contents

1. Experimental section- organic chemistry part
    1.1. Synthetic schemes
    1.2. General procedure for preparation of azido methyl uridine monophosphate and triphosphate (**dT^{N3}MP**, **dT^{N3}TP**)
    1.3. Synthesis of reactive moieties
        1.3.1. Synthesis of oct-7-yne-2,4-dione (**HDO**)
        1.3.2. Synthesis of 3-(prop-2-yn-1-yl) pentane-2,4-dione (**PDO**)
    1.4. Synthesis of modified monophosphates and triphosphates
        1.4.1 Synthesis of **dT^{HDO} MP**
        1.4.2 Synthesis of **dT^{PDO}MP**
        1.4.3 Synthesis of **dT^{HDO} TP**
        1.4.4 Synthesis of **dT^{PDO} TP**
    1.5 Cross-linking model reactions of **dT^{HDO} MP** and N-Boc-L-Arg-OH and N-Boc-L-AlaArgAla-OH
        1.5.1 Synthesis of **dT^{HDOArg} MP**
        1.5.2 Synthesis of **dT^{HDOARA}MP**
    1.6 Cross-linking model reactions of **dT^{HDO} MP** with NH_2AlaGlyAla-OH , N-Ac-L-Ala-LysAla-OH and N-Ac-AlaArgAla-OH
        1.6.1 Synthesis of **dT^{HDOAGA} MP**
        1.6.2 Synthesis of **dT^{HDOAKA} MP**
        1.6.3 Synthesis of **dT^{HDOARA} MP**
    1.7. Stability studies of the conjugates **dT^{HDOAGA}MP**, **dT^{HDOAKA}MP**, **dT^{HDOARA}MP** in H_2O

2. Experimental section- biochemistry part

2.1.1. List of sequences of primers, templates and oligonucleotides used in this study

2.2. Enzymatic synthesis of **HDO**- and **PDO**-modified DNA

2.2.1. Single incorporation of **dT$^{HDO/PDO}$TP** using 19-mer template -Analytical scale

2.2.2. Single incorporation of **dT$^{HDO/PDO}$TP** using 30-mer template - Analytical scale

2.2.3. Single incorporation of two **dT^{HDO}TP** using 30-mer template -Analytical scale

2.2.4. Single incorporation of four **dT^{HDO}TP** using 31-mer template- Analytical scale

2.2.5. Single incorporation of **dT$^{HDO/PDO}$TP** using 19-mer template – Semi-preparative scale

2.2.6. Single incorporation of **dT$^{HDO/PDO}$TP** using 30-mer template- Semi-preparative scale

2.2.7. DB Streptavidin magneto separation procedure

2.2.8. MALDI-TOF data of **dT^{HDO}**-modified oligonucleotides

2.3. Bio-conjugation reactions

2.3.1 Structures of peptides used in this study

2.3.2 Reaction of **19DNA_T^{HDO}** with N-Boc-L-Arg-OH - PAGE analysis

2.3.3 Reaction of **19DNA_T^{HDO}** with **30DNA_T^{HDO}** with N-Boc-L-Arg-OH and arginine containing peptides - PAGE analysis

2.3.4 Reaction of **19DNA_T^{HDO}** with **30DNA_T^{HDO}** with N-Boc-L-Arg-OH and arginine containing peptides - PAGE analysis

2.3.5 MALDI-TOF data of arginine-modified oligonucleotides adducts

2.4 Reactions of **30DNA_T$^{HDO/PDO}$** with lysine and arginine containing peptides-PAGE analysis

2.4.1 Reaction with heptapeptides as Ac-(Lys)$_7$-NH$_2$ and Ac-(Arg)$_7$-NH$_2$

2.4.2 Reaction with tripeptides as Ac-L-AlaLysAla-OH and Ac-L-AlaArgAla-OH

2.4.3 Reaction with hexapeptide Ac-GAKKAA-NH$_2$ and Ac-KGAAKA-NH$_2$.

2.4.4 Reaction with decapeptides as Ac-L-SGYTARAESG-NH$_2$ and Ac- L- SGYTAKAESG-NH$_2$

2.4.5 Reaction with decapeptide Ac- L- SGYTAKAESG-NH$_2$ and further reductive amination

2.4.6 Reaction with tripeptide NH$_2$-AlaGlyAla-OH

2.4.7 Reaction with undecapeptide Ac-L-AHIDKLEMSTA-NH_2 and decapeptide Ac-L-FNEWCGPYVA-NH_2
2.4.8 List of amino acids and synthetic peptides used in cross-linking reactions with **19/30DNA_T^{HDO}** and **30DNA_T^{PDO}** and conversions of cross-linking reactions.
2.5 Cross-linking of **DNA_T^{HDO}** and individual recombinant proteins (BSA, H2A, H2B, H3.1 and H4)-SDS analysis
2.5.1 Scheme of cross linking of modified **DNA_T^{HDO}** with Histone recombinant proteins
2.5.2 Reaction of **30DNA_T^{HDO}** with individual recombinant proteins
2.5.3 Reaction of **30DNA_T^{HDO}** with individual recombinant proteins and further treatment with NH_2OH
2.5.4 Reaction of **30DNA_T^{HDO}** with individual recombinant proteins (ratio 1:1)
2.5.5 Reaction of **19DNA_T^{HDO}** with individual recombinant proteins
2.5.6 Comparison of cross-linking of **30DNA_T^{HDO}** and **30DNA_T^{PDO}** with H4 protein- SDS analysis
2.6 Kinetic studies of cross-linking of **30DNA_T^{HDO}** with H4 protein- SDS analysis
2.7 Stability studies of **30DNA_T^{HDO}** and **30DNA_T^{PDO}** in different buffers- PAGE analysis
2.8 Cross-linking of **25DNA_T^{HDO}** and GSTp53CD
2.9 Characterization of cross-links between **30DNA_T^{HDO}** and individual recombinant proteins (BSA, H2A, H2B, H3.1 and H4) by mass spectrometry
2.9.1 Overview of DNA-protein cross-linking reactions and their conversions
2.9.2 Overview of DNA protein conjugates and MS analysis results.
3. Copies of mass spectra
3.1.MALDI TOF spectra
3.2.ESI-spectra
4. Copies of HPLC chromatograms
5. Copies of NMR spectra
6. References

1. Experimental section-organic chemistry part

General remarks for the synthetic part

^{1}H, ^{13}C and ^{31}P NMR spectra were acquired on a Bruker AVANCE IIIHD 600 (^{1}H at 600.1 MHz, ^{13}C at 150.9 MHz), Bruker AVANCE IIIHD 500 (^{1}H at 500.0 MHz, ^{13}C at 125.7 MHz, ^{31}P at 202.4 MHz) and JEOL ECZR 500 (^{1}H at 500.2 MHz, ^{13}C at 125.8 MHz, ^{31}P at 202.5) spectrometers, as indicated. ^{1}H and ^{13}C resonances were fully assigned using H,H-COSY, H,H-ROESY, H,C-HSQC and H,C-HMBC techniques. All chemical shifts are quoted on the δ scale in ppm and referenced using residual ^{1}H solvent signal in ^{1}H NMR spectra ($\delta(CHCl_3)$ = 7.26 ppm; $\delta(CHD_2OD)$ = 3.31 ppm; and ^{13}C solvent signal in ^{13}C NMR spectra ($\delta(CDCl_3)$ = 77.0 ppm; $\delta(CD_3OD)$ = 49.0 ppm). NMR spectra measured in D_2O were referenced to the signal of *t*-BuOH (10% v/v solution in D_2O, 1 drop) as the internal standard (1.24 ppm in ^{1}H, 32.43 ppm in ^{13}C). ^{31}P NMR spectra were referenced to H_3PO_4 signal (0 ppm) as the external standard. Coupling constants (*J*) are reported in Hz with the following splitting abbreviations: s = singlet, d= doublet, t = triplet, q = quartet, m = multiplet.. High resolution mass spectra were measured on LTQ Orbitrap XL (Thermo Fisher Scientific) using Electrospray Ionization Technique (ESI). Reactions were monitored by thin layer chromatography (TLC) on TLC Silica gel 60 F254 (Merck) and detected by UV (254 nm) or by solution of 4-anisaldehyde 3.6 v/v in ethanol and 10% v/v of sulphuric acid. Reactions were monitored by Advion Expression Compact Mass Spectrometer connected with Plate Express® TLC Plate Reader using ESI. High performance flash chromatography (HPFC) was performed with Biotage SP1 apparatus on DEAE SEPHADEX A-25 sodium form columns or with ISCO Combiflash Rf system on RediSep Rf Gold Silica Gel Disposable columns. Purification of nucleoside triphosphates and monophosphates was performed using HPLC (Waters modular HPLC system) on a column packed with 10 µm C18 reversed phase (Phenomenex, Luna C18 (2) 100 Å). Reactions were analysed by TLC using IPAV (Isopropyl alcohol /NH_4OH /H_2O, ratio 11/7/2) as mobile phase. All materials were purchased from commercial suppliers and used without further purification unless otherwise stated. $POCl_3$ and $PO(OMe)_3$ were distilled prior to use. The water used in synthetic part was of HPLC quality. Chemicals were of analytical grade.

1.1. Synthetic schemes

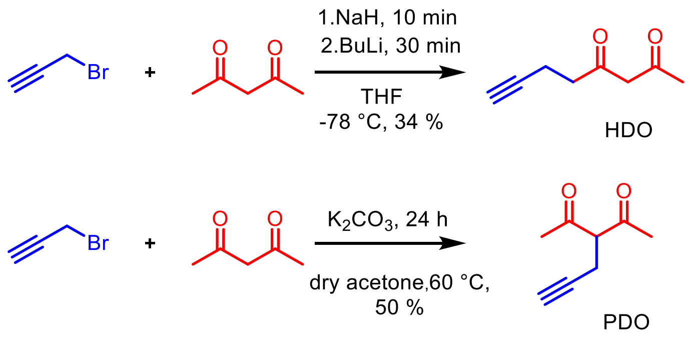

Scheme S1. Chemical synthesis of oct-7-yne-2,4-dione (**HDO**) and 3-(prop-2-yn-1-yl) pentane-2,4-dione (**PDO**) moieties used in the study.

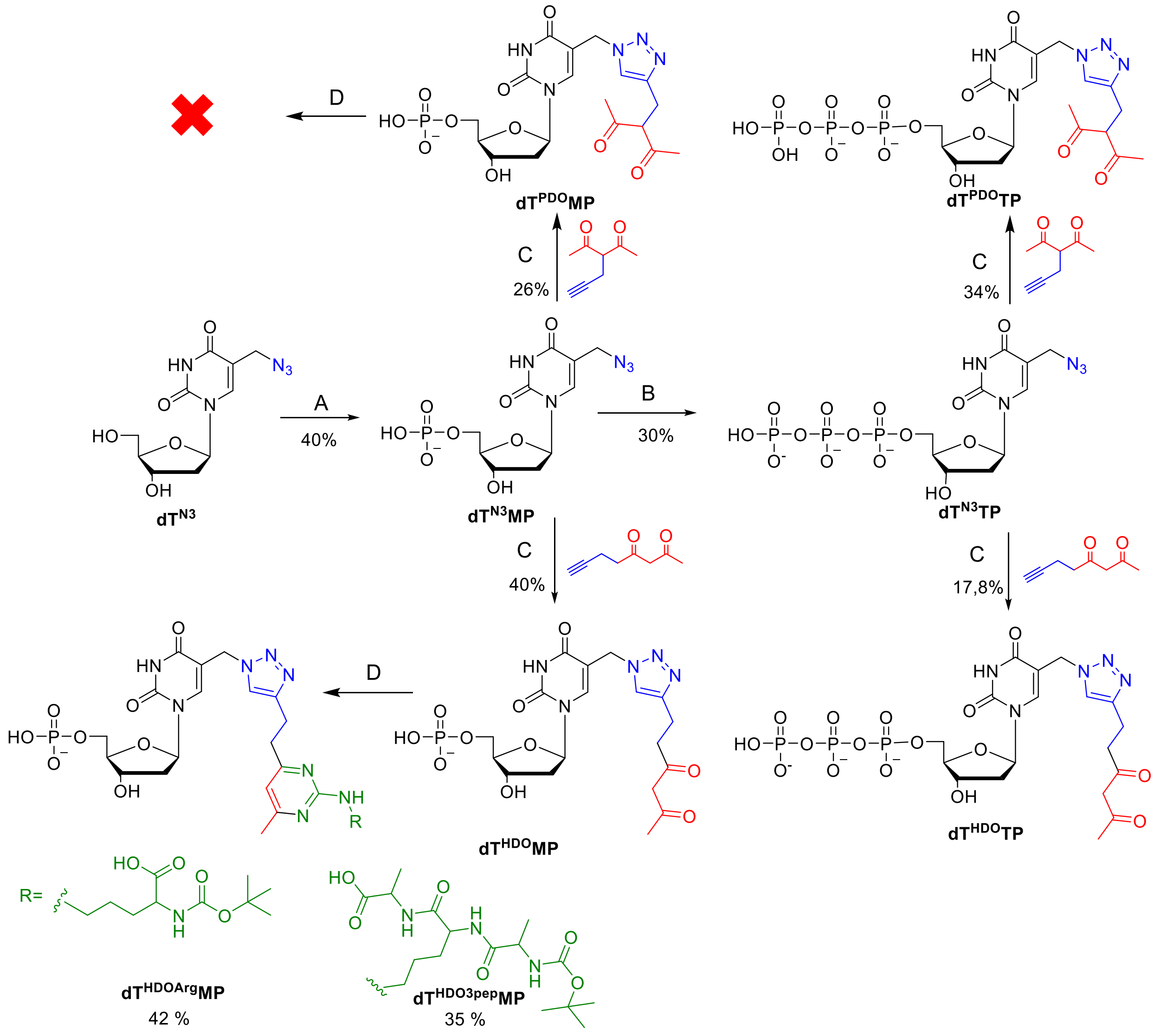

Scheme S2. Chemical synthesis of HDO- and PDO-modified 2'-deoxyuridine monophosphate and triphosphate and 2'-deoxyuridine monophosphate adducts with N-Boc-Arg-OH and arginine containing tripeptide as N-Boc-L-AlaArgAla-OH. Conditions A) $POCl_3$, $PO(OMe)_3$, 0°C, 6h. B)$(NHBu_3)_2H_2P_2O_7$, DMF, Bu_3N, 0°C, 2h; 2M TEAB, H_2O. C) $CuSO_4*H_2O$, $C_6H_7NaO_6$, H2O/tBuOH (1:1), rt, 1-18h. D) N-Boc-Arg-OH or N-Boc-L-AlaArgAla-OH, $NaHCO_3$ buffer pH 10, rt, 18-72h.

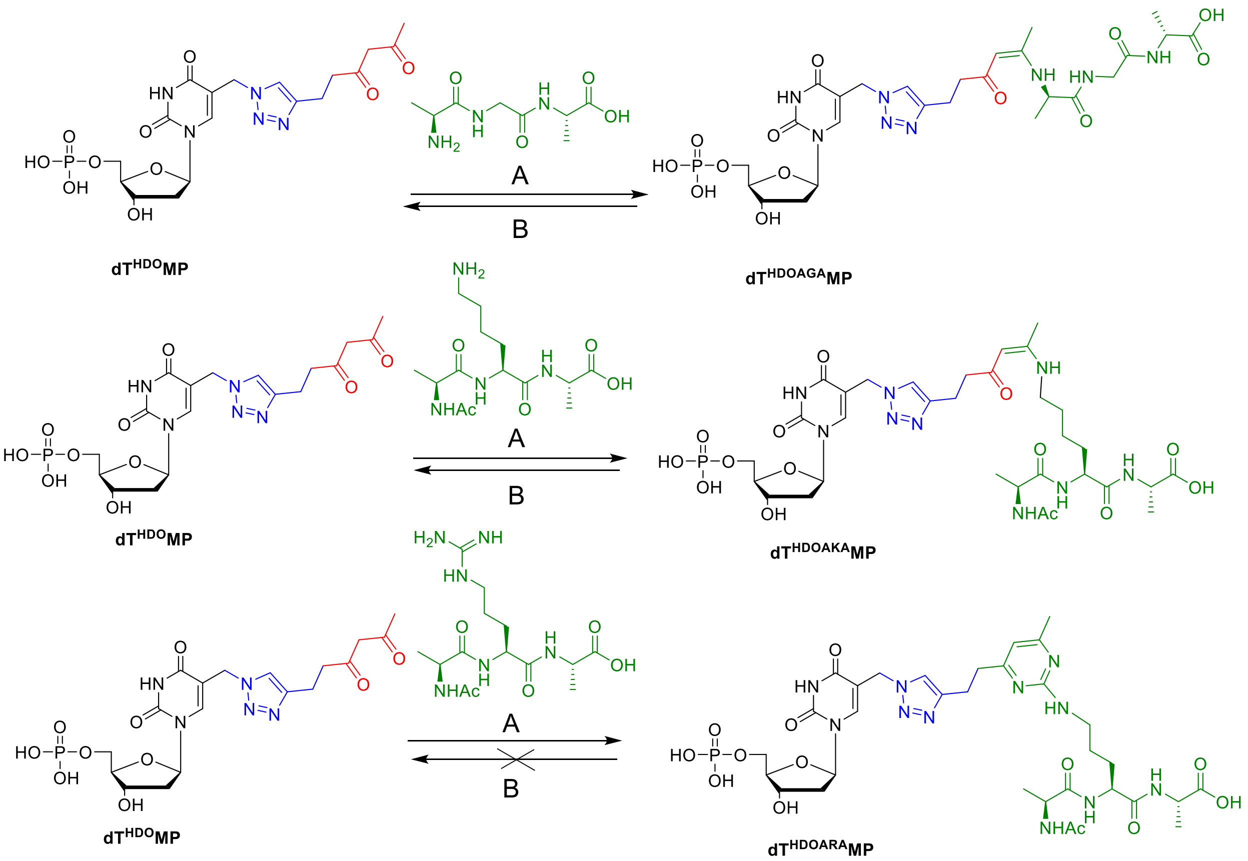

Scheme S3 A) Cross-linking model reactions of **dT^{HDO}MP** with α-NH_2, lysine and arginine containing tripeptides. Conditions: $NaHCO_3$ buffer 0.5 M pH 10, 25°C for 18 h. B) Stability studies of adducts **dT^{HDOAGA}MP**, **dT^{HDOAKA}MP** and **dT^{HDOARA}MP** in H_2O at 25°C for 20 h.

1.2. General procedure for preparation of azido thymidine monophosphate and triphosphate ($dT^{N3}MP$, $dT^{N3}TP$)

Compound **dT^{N3}** (0.200 g, 0.550 mmol) was dried at 25 °C overnight in vacuo. After cooling on ice, $PO(OMe)_3$ (1.5 mL) and $POCl_3$ (100 µL) were added under argon atmosphere. The reaction mixture was stirred for 6 h at 0°C. The reaction was stopped by addition of TEAB (2 M, 1 mL) and H_2O (0.6 mL). *For the triphosphate:* In a separate flask, the mixture of $(NHBu_3)_2H_2P_2O_7$ (1.5 g) and Bu_3N (600 µL) in dry DMF (6 mL) was prepared under argon atmosphere, cooled to 0 °C and then added by syringe to the reaction mixture. The mixture was stirred at 0 °C for 2 h. The product was purified by SEPHADEX Na^+ using H_2O/TEAB 2 M buffer as eluent. Subsequently the product was purified on RP-HPLC with use of linear gradient of 0.1 M TEAB (triethylammonium bicarbonate in H_2O) to 0.1 M TEAB in H_2O/MeOH (1:1) to MeOH as eluent. Several co-distillations with water followed by freeze-drying from water gave product **$dT^{N3}MP$** as yellow powder (103 mg, 40 % yield) and product **$dT^{N3}TP$** (111 mg, 30 % yield). Spectral data were in accordance with literature[1,2].

1.3. Synthesis of reactive moieties:

1.3.1. Synthesis of oct-7-yne-2,4-dione (HDO)

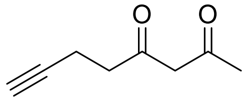

To a stirred solution of NaH (65.9 mg, 2.74 mmol) in THF was added acetoacetone (250 mg, 2.69 mmol). The solution was stirred for 10 min. After 10 min the reaction mixture was cooled to -78 °C and BuLi (1.56 mL 1.6 M in THF) was added slowly drop by drop. The solution was stirred for 30 min. Propargyl bromide (306 µL 2.75 mmol) was added and the solution was warmed to 25 °C and stirred it for other 3 h. The solution was quenched with NH_4Cl at 0 °C, diluted with EtOAc and washed 3 times with H_2O and brine. The organic fraction was dried under $MgSO_4$ and the crude mixture was purified by column chromatography using linear gradient of DCM/MeOH (0-20 %) to give a yellow oil as a product (117 mg, 34 % yield). The NMR spectra were in accordance with literature[3].

1.3.2. Synthesis of 3-(prop-2-yn-1-yl) pentane-2,4-dione (PDO)

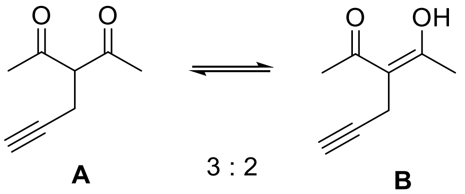

K_2CO_3 (1.49 g, 0.10 mol) was placed in a flask and then dried acetone was added (100 mL). 1 mL of propargyl bromide (1.57 g, 0.013 mol) and acetylacetone (4.4 g, 0.044 mol) were added simultaneously, and the reaction was stirred for 24 h at 60° C. The mixture was filtrated and the solvent evaporated. The product was purified by flesh chromatography (Hex:DCM 7:3). The product was isolated as a yellow oil (90 mg, 49 % yield). Rf = 0.8 (Hex:DCM 7:3)[4].

^{1}H NMR (500.2 MHz, $CDCl_3$): 2.02 (t, 2H, ^4J = 2.7, **H**C≡C-*A,B*); 2.21 (s, 6H, CH_3-*B*); 2.24 (s, 6H, CH_3-*A*); 2.68 (dd, 2H, ^3J = 7.5, ^4J = 2.7, C**H₂**C≡CH-*A*); 3.10 (d, 2H, ^4J = 2.7, C**H₂**C≡CH-*B*); 3.84 (t, 1H, ^3J = 7.5, CH-*A*); 16.50 (s, 1H, OH-*B*).

^{13}C NMR (125.8 MHz, $CDCl_3$): 17.33 (**C**H_2C≡CH-*A,B*); 23.07 (CH_3-*B*); 29.30 (CH_3-*A*); 66.63 (CH-*A*); 68.68 (H**C**≡C-*B*); 70.76 (H**C**≡C-*A*); 80.21 (HC≡**C**-*A*); 81.60 (H**C**≡C-*B*); 106.42 (**C**=C(OH)CH_3-*B*); 190.89 (CO-*B*); 202.17 (CO-*A*).

HR/MS (ESI$^+$) for $C_8H_{10}O_2Na$: 161.5736 $[M+Na]^+$ calculated, found 161.05730 $[M+Na]^+$.

1.4. Synthesis of modified monphosphates and triphosphates (dT^{HDO}MP, dT^{HDO}TP, dT^{PDO}MP, dT^{PDO}TP)

1.4.1.Synthesis of (2R,3S,5R)-5-{5-[(4-(3,5-dioxohexyl)-1H-1,2,3-triazol-1-yl]methyl}-2,4-dioxo-3,4-dihydropyrimidin-1[(2H)-yl]-3-hydroxytetrahydrofuran-2-yl methyl phosphate (dT^{HDO} MP)

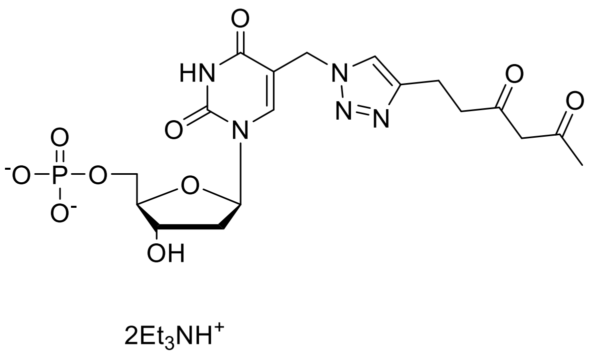

dT^{N3}MP (36.9 mg, 0.1 mmol) was dissolved in 1 mL of H_2O/tBuOH (1:1) in a pressure tube and degassed for 10 min. $CuSO_4$(II) pentahydrate (4.17 g, 0.20% mol) was dissolved in 0.5 mL of H_2O/*t*BuOH (1:1) in a pressure tube and degassed for 10 min. A solution of oct-7-yne-2,4-dione (55.36mg, 0.4 mmol) in DMSO (100 µL) was degassed for 10 min. To the $CuSO_4$ solution was added the **dT^{N3}MP** solution, then the oct-7-yne-2,4-dione solution and subsequently sodium ascorbate solution (80.52 mg, 0.4 mmol). The reaction mixture was degassed for 10 min and stirred for 1.5 h. The reaction was quenched by the addition of 5 M EDTA (1 mL) solution. The product was purified on RP-HPLC with use of linear gradient of 0.1 M TEAB (Triethylammonium bicarbonate in H_2O) to 0.1 M TEAB in H_2O/MeOH (1:1) to MeOH as eluent. Several co-distillations with water followed by freeze drying from water gave the product **dT^{HDO}MP** as red powder (20 mg, 40 % yield). Rf =0.5 (IPAV).

^{1}H NMR (600.1 MHz, D_2O): 1.27 (t, 18H, J_{vic} = 7.3, C**H$_3$**CH$_2$N); 2.21 (s, 3H, CH$_3$CO, partially exchanged by ^{2}H); 2.38 – 2.46 (m, 2H, H-2′); 2.90 – 2.97 (m, 2H, C**H$_2$**CH$_2$CO); 2.96 – 3.02 (m, 2H, CH$_2$C**H$_2$**CO); 3.20 (q, 12H, J_{vic} = 7.3, CH$_3$C**H$_2$**N); 4.02 (ddd, 1H, J_{gem} = 11.6, $J_{H,P}$ = 5.1, $J_{5'b,4'}$ = 3.5, H-5′b); 4.06 (ddd, 1H, J_{gem} = 11.6, $J_{H,P}$ = 4.5, $J_{5'a,4'}$ = 3.2, H-5′a); 4.21 (dddd, 1H, $J_{4',5'}$ = 3.5, 3.2, $J_{4',3'}$ = 2.9, $J_{H,P}$ = 1.8, H-4′); 4.60 (ddd, 1H, $J_{3',2'}$ = 5.5, 4.2, $J_{3',4'}$ = 2.9, H-3′); 5.34 (s, 2H, CH$_2$N); 6.33 (t, 1H, $J_{1',2'}$ = 6.8, H-1′); 7.81 (s, 1H, H-5-triazole); 8.34 (s, 1H, H-6); COCH$_2$CO exchanged by ^{2}H.

^{13}C NMR (150.9 MHz, D_2O): 11.06 (**C**H$_3$CH$_2$N); 21.51 (**C**H$_2$CH$_2$CO); 33.24 (**C**H$_3$CO); 41.96 (CH$_2$-2′); 45.23 (CH$_2$**C**H$_2$CO); 49.51 (CH$_3$**C**H$_2$N); 49.66 (CH$_2$N); 58.83 (CO**C**H$_2$CO); 66.88 (d, $J_{C,P}$ = 4.6, CH$_2$-5′); 73.91 (CH-3′); 88.48 (CH-1′); 89.01 (d, $J_{C,P}$ = 8.6, CH-4′); 111.48 (C-5); 126.20 (CH-5-triazole); 145.05 (CH-6); 149.43 (C-4-triazole); 154.33 (C-2); 167.33 (C-4); 211.05 (**C**OCH$_2$COCH$_3$); 211.13 (COCH$_2$**C**OCH$_3$)

^{31}P{^{1}H} NMR (202.4 MHz, D_2O): 2.74.

HR/MS (ESI$^-$) for $C_{18}H_{23}N_5O_{10}P^-$ 500.11880 [M-H]$^-$ calculated, found 500.11863 [M-H]$^-$

1.4.2. Synthesis of (2R,3S,5R)-5-{5-[4-(2-acetyl-3-oxobutyl)-1H-1,2,3-triazol-1-yl] methyl}-2,4-dioxo-3,4-dihydropyrimidin-[(2H)-yl-3-hydroxytetrahydrofuran-2-yl] methyl hydrogen phosphate (dT^{PDO} MP)

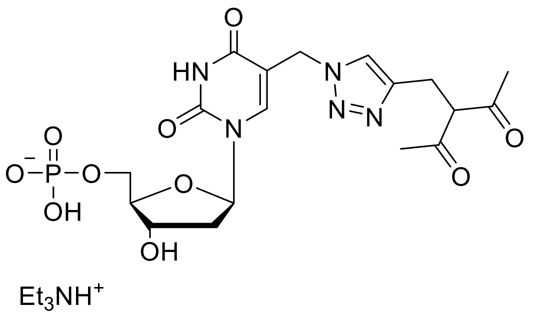

dT^N3^MP (100 mg, 0.27 mmol) was dissolved in 1.5 mL of H_2O/*t*BuOH (1:1) in a pressure tube and degassed for 10 min. $CuSO_4$(II) pentahydrate (4.17, g, 0.20 % mol) was dissolved in 1 mL of H_2O/*t*BuOH (1:1) in a pressure tube and degassed for 10 min. 3-(prop-2-yn-1-yl) pentane-2,4-dione (92 mg, 0.672 mmol) was degassed for 10 min. To the $CuSO_4$ solution was added the **dT^N3^MP** solution, then 3-(prop-2-yn-1-yl) pentane-2,4-dione and subsequently the sodium ascorbate solution (80.52 mg, 0.4 mmol). The reaction mixture was degassed for 10 min until the color changed to yellow and stirred for 1 h. The reaction was quenched by the addition of 5 M EDTA solution (1 mL). The product was purified on RP-HPLC with use of linear gradient of 0.1 M TEAB (triethylammonium bicarbonate in H_2O) to 0.1 M TEAB in H_2O/MeOH (1:1) to MeOH as eluent. Several co-distillations with water followed by freeze drying from water gave the product **dT^PDO^MP** as red powder (37 mg, 26 % yield). Rf =0.5 (IPAV).

^{1}H NMR (500.0 MHz, D_2O): 1.27 (t, 9H, J_{vic} = 7.3, C**H₃**CH₂N); 2.25 (s, 6H, CH₃CO); 2.42 (dd, 2H, $J_{2',1'}$ = 6.8, $J_{2',3'}$ = 4.8, H-2′); 3.19 (s, 2H, CH₂C); 3.20 (q, 6H, J_{vic} = 7.3, CH₃C**H₂**N); 4.04 (ddd, 1H, J_{gem} = 11.6, $J_{H,P}$ = 5.0, $J_{5'b,4'}$ = 3.3, H-5′b); 4.09 (ddd, 1H, J_{gem} = 11.6, $J_{H,P}$ = 4.5, $J_{5'a,4'}$ = 3.0, H-5′a); 4.21 (dtd, 1H, $J_{4',5'}$ = 3.3, 3.0, $J_{4',3'}$ = 3.0, $J_{H,P}$ = 1.9, H-4′); 4.60 (td, 1H, $J_{3',2'}$ = 4.8, $J_{3',4'}$ = 3.0, H-3′); 5.31, 5.35 (2 × d, 2 × 1H, J_{gem} = 14.9, CH₂N); 6.34 (t, 1H, $J_{1',2'}$ = 6.8, H-1′); 7.83 (s, 1H, H-5-triazole); 8.32 (s, 1H, H-6); CH(COMe)₂ exchanged by ^{2}H.

^{13}C NMR (125.7 MHz, D_2O): 11.06 (**C**H₃CH₂N); 26.24 (**C**H₂C); 32.73, 32.75 (**C**H₃CO); 42.05 (CH₂-2′); 49.51 (CH₃**C**H₂N); 49.77 (CH₂N); 67.08 (d, $J_{C,P}$ = 4.6, CH₂-5′); 68.35 (**C**H(COMe)₂); 73.89 (CH-3′); 88.53 (CH-1′); 88.92 (d, $J_{C,P}$ = 8.7, CH-4′); 111.35 (C-5); 126.88 (CH-5-triazole); 144.99 (CH-6); 146.88 (C-4-triazole); 154.32 (C-2); 167.27 (C-4); 211.42 (**C**OCH₃).

^{31}P{^{1}H} NMR (202.4 MHz, D_2O): 2.02.

HR/MS (ESI$^-$) for $C_{18}H_{23}N_5O_{10}P^-$ 500.11880 [M-H]$^-$ calculated, found 500.11807 [M-H]$^-$.

1.4.3. Synthesis of (2R,3S,5R)-5-{[5-((4-(3,5-dioxohexyl)-1H-1,2,3-triazol-1-yl] methyl}-2,4-dioxo-3,4-dihydropyrimidin-1[(2H)-yl-3-hydroxytetrahydrofuran-2-yl] methyl hydrogen triphosphate (dT^{HDO}TP)

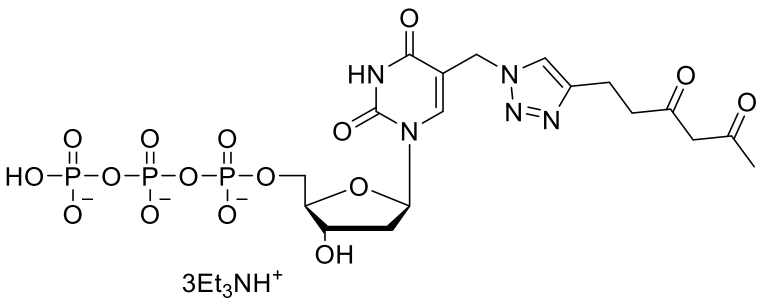

dT^{N3}TP (28.8 mg, 0.05 mmol) was dissolved in 0.5 mL of H_2O/*t*BuOH (1:1) in a pressure tube and degassed for 10 min. $CuSO_4$(II) pentahydrate (2.75 mg, 20 %mol) was dissolved in 0.5 mL of H_2O/*t*BuOH (1:1) in a pressure tube and degassed for 10 min. A solution of oct-7-yne-2,4-dione (30.41 mg, 0.022 mmol) in DMSO (100 μL) was degassed for 10 min. To the $CuSO_4$ solution was added the **dT^{N3}TP** solution, the oct-7-yne-2,4-dione solution and followed by the sodium ascorbate solution (43.6 mg, 0.22 mmol). The reaction mixture was degassed for 10 min and stirred for 18h. The reaction was stopped by the addition of 5 M EDTA (1mL) solution. The product was purified on RP-HPLC with use of linear gradient of 0.1 M TEAB (Triethylammonium bicarbonate in H_2O) to 0.1 M TEAB in H_2O/MeOH (1:1) to MeOH as eluent. Several co-distillations with water followed by freeze drying from water gave the product **dT^{HDO}TP** as red powder (6.5 mg, 17.8 % of yield) Rf =0.80 (IPAV).

^{1}H NMR (500.0 MHz, D_2O): 1.27 (t, 27H, J_{vic} = 7.3, C**H$_3$**CH$_2$N); 2.21 (s, 3H, CH$_3$CO, partially exchanged by ^{2}H); 2.38 – 2.48 (m, 2H, H-2′); 2.91 – 2.96 (m, 2H, C**H$_2$**CH$_2$CO); 2.97 – 3.02 (m, 2H, CH$_2$C**H$_2$**CO); 3.20 (q, 18H, J_{vic} = 7.3, CH$_3$C**H$_2$**N); 4.20 – 4.31 (m, 3H, H-4′,5′); 4.71 (m, 1H, H-3′); 5.32, 5.36 (2 × d, 2 × 1H, J_{gem} = 14.7, CH$_2$N); 6.33 (t, 1H, $J_{1',2'}$ = 6.7, H-1′); 7.83 (s, 1H, H-5-triazole); 8.29 (s, 1H, H-6); COCH$_2$CO exchanged by ^{2}H.

^{13}C NMR (125.7 MHz, D_2O): 11.06 (**C**H$_3$CH$_2$N); 21.54 (**C**H$_2$CH$_2$CO); 33.26 (**C**H$_3$CO); 41.82 (CH$_2$-2′); 45.27 (CH$_2$**C**H$_2$CO); 49.50 (CH$_3$**C**H$_2$N); 49.70 (CH$_2$N); 58.85 (CO**C**H$_2$CO); 68.02 (d, $J_{C,P}$ = 5.2, CH$_2$-5′); 73.27 (CH-3′); 88.43 (CH-1′); 88.64 (d, $J_{C,P}$ = 9.0, CH-4′); 111.49 (C-5); 126.12 (CH-5-triazole); 144.91 (CH-6); 149.44 (C-4-triazole); 154.36 (C-2); 167.36 (C-4); 211.10 (**C**OCH$_2$**C**OCH$_3$).

$^{31}P\{^1H\}$ NMR (202.4 MHz, D_2O): -22.12 (bt, J = 20.0, P_β); -10.83 (d, J = 20.0, P_α); -8.05 (bs, P_γ). HR/MS (ESI$^-$) for $C_{18}H_{25}N_5O_{16}P_3$ 660.05146 [M-H]$^-$ calculated, found 660.05072 [M-H]$^-$.

1.4.4. Synthesis of (2R,3S,5R)-5-{[5-((4-(2-acetyl-3-oxobutyl)-1H-1,2,3-triazol-1-yl] methyl}-2,4-dioxo-3,4-dihydropyrimidin-1[(2H)-yl))-3-hydroxytetrahydrofuran-2-yl] methyl triphosphate (dT^{PDO}TP)

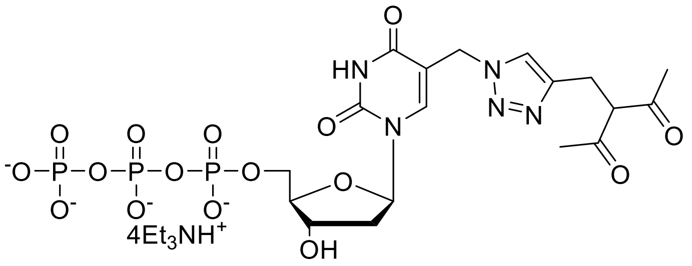

dT^{N3}TP (28.8 mg, 0.05 mmol) was dissolved in 0.5 mL of H_2O/*t*BuOH (1:1) in a pressure tube and degassed for 10 min. $CuSO_4$(II) pentahydrate (2.75 mg, 20 %mol) was dissolved in 0.5 mL of H_2O/tBuOH (1:1) in a pressure tube and degassed for 10 min. was degassed for 10 min. To the CuSO4 solution was added the **dT^{N3}TP** solution. 3-(prop-2-yn-1-yl) pentane-2,4-dione **(**31.2mg, 0.22 mmol) was added followed by sodium ascorbate solution (43.6 mg, 0.22 mmol). The reaction mixture was degassed for 10 min and stirred for 18h. The reaction was stopped by the addition of 5M EDTA (1 mL) solution. The product was purified on RP-HPLC with use of linear gradient of 0.1 M TEAB (Triethylammonium bicarbonate in H_2O) to 0.1 M TEAB in H_2O/MeOH (1:1) to MeOH as eluent. Several co-distillations with water followed by freeze drying from water gave the product **dT^{PDO}TP** as white powder (12.3 mg 34 % yield). Rf = 0.80 (IPAV)

^{1}H NMR (500.0 MHz, D_2O): 1.25 (t, 36H, J_{vic} = 7.3, C**H$_3$**CH$_2$N); 2.23 (s, 6H, CH$_3$CO); 2.35 – 2.46 (m, 2H, H-2′); 3.166 (s, 2H, CH$_2$C); 3.169 (q, 24H, J_{vic} = 7.3, CH$_3$C**H$_2$**N); 4.19 (m, 1H, H-4′); 4.20 – 4.29 (m, 2H, H-5′); 4.69 (dt, 1H, $J_{3',2'}$ = 5.8, 4.0, $J_{3',4'}$ = 4.0, H-3′); 5.30, 5.34 (2 × d, 2 × 1H, J_{gem} = 14.8, CH$_2$N); 6.30 (t, 1H, $J_{1',2'}$ = 6.6, H-1′); 7.84 (s, 1H, H-5-triazole); 8.27 (s, 1H, H-6); CH(COMe)$_2$ exchanged by ^{2}H.

^{13}C NMR (125.7 MHz, D_2O): 11.07 (**C**H$_3$CH$_2$N); 26.29 (**C**H$_2$C); 32.77, 32.79 (**C**H$_3$CO); 41.79 (CH$_2$-2′); 49.47 (CH$_3$**C**H$_2$N); 67.94 (d, $J_{C,P}$ = 5.6, CH$_2$-5′); 68.41 (**C**H(COMe)$_2$); 73.14 (CH-3′);

88.38 (CH-1′); 88.64 (d, $J_{C,P}$ = 9.1, CH-4′); 111.36 (C-5); 126.81 (CH-5-triazole); 144.96 (CH-6); 146.90 (C-4-triazole); 154.36 (C-2); 167.30 (C-4); 211.44 (**C**OCH_3).

$^{31}P\{^1H\}$ NMR (202.4 MHz, D_2O): -22.27 (t, J = 20.3, P_β); -10.88 (d, J = 20.3, P_α); -7.72 (bd, J = 20.3, P_γ).

HR/MS (ESI^-) for $C_{18}H_{25}N_5O_{16}P_3$ 660.05146 $[M-H]^-$ calculated, found 660.05072 $[M-H]^-$.

1.5. Cross-linking model reactions between dT^{HDO} MP N-Boc-L-Arginine and N-Boc-L-AlaArgAla-OH

1.5.1. Synthesis of (2R,3S,5R)-5-{[5-((4-(2-(2-(((S)-4-((tert-butoxycarbonyl)amino)-4-carboxybutyl)amino)-6-methylpyrimidin-4-yl)ethyl)-1H-1,2,3-triazol-1-yl]methyl}-2,4-dioxo-3,4-dihydropyrimidin-1[(2H)-yl)-3-hydroxytetrahydrofuran-2-yl]methyl phosphate (dT^{HDOArg}MP)

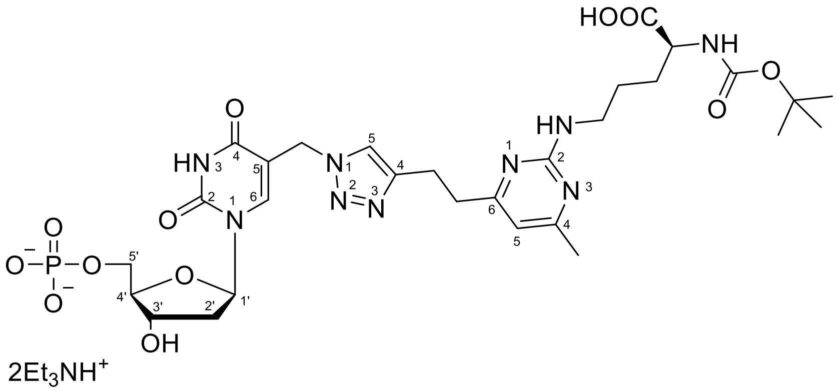

dT^{HDO}MP (7.6 mg, 0.015 mmol) and N-Boc-L-Arginine (12.47 mg 0.045 mmol) were dissolved in $NaHCO_3/Na_2CO_3$ buffer (0.5 M, pH 10, 240 μL) and the mixture was stirred for 24 h at 25 °C. The product was purified on RP-HPLC with use of linear gradient of 0.1 M TEAB (triethylammonium bicarbonate in H_2O) to 0.1 M TEAB in H_2O/MeOH (1:1) to MeOH as eluent. Several co-distillations with water followed by freeze drying gave the product as a yellow powder **dT^{HDOArg}MP** (4.8 mg, 42.8 % yield,) Rf =0.85 (IPAV).

^1H NMR (500.0 MHz, D_2O): 1.27 (t, 18H, J_{vic} = 7.3, C**H$_3$**CH_2N); 1.34 (s, 9H, $(CH_3)_3C$); 1.58 – 1.71 (m, 3H, H-4,3b-Arg); 1.82 (m, 1H, H-3a-Arg); 2.21 (s, 3H, CH_3); 2.41 (dd, 2H, $J_{2',1'}$ = 6.9, $J_{2',3'}$ = 4.8, H-2′); 2.84 – 2.90 (m, 2H, triazine-CH_2C**H$_2$**-pyrimidine); 3.04 – 3.09 (m, 2H, triazine-

C**H**$_2$CH$_2$-pyrimidine); 3.20 (q, 12H, J_{vic} = 7.3, CH$_3$C**H**$_2$N); 3.27 – 3.39 (m, 2H, H-5-Arg); 3.91 (m, 1H, H-2-Arg); 4.01 (ddd, 1H, J_{gem} = 11.6, $J_{H,P}$ = 5.1, $J_{5'b,4'}$ = 3.5, H-5′b); 4.04 (ddd, 1H, J_{gem} = 11.6, $J_{H,P}$ = 4.6, $J_{5'a,4'}$ = 3.3, H-5′a); 4.19 (dddd, 1H, $J_{4',5'}$ = 3.5, 3.3, $J_{4',3'}$ = 3.0 $J_{H,P}$ = 1.7, H-4′); 4.60 (ddd, 1H, $J_{3',2'}$ = 4.8, $J_{3',4'}$ = 3.0, H-3′); 5.30 (s, 2H, CH$_2$N); 6.30 (s, 1H, H-5-pyrimidine); 6.32 (t, 1H, $J_{1',2'}$ = 6.9, H-1′); 7.62 (s, 1H, H-5-triazole); 8.32 (s, 1H, H-6).
^{13}C NMR (125.7 MHz, D$_2$O): 11.06 (**C**H$_3$CH$_2$N); 25.32 (CH$_3$); 26.35 (triazine-**C**H$_2$CH$_2$-pyrimidine); 27.99 (CH$_2$-4-Arg); 30.46 ((**C**H$_3$)$_3$C); 32.10 (CH$_2$-3-Arg); 38.62 (triazine-CH$_2$**C**H$_2$-pyrimidine); 41.95 (CH$_2$-2′); 43.45 (CH$_2$-5-Arg); 49.50 (CH$_2$N, CH$_3$**C**H$_2$N); 58.71 (CH-2-Arg); 66.84 (d, $J_{C,P}$ = 4.6, CH$_2$-5′); 73.95 (CH-3′); 83.62 ((CH$_3$)$_3$**C**); 88.45 (CH-1′); 89.06 (d, $J_{C,P}$ = 8.5, CH-4′); 111.68 (C-5); 112.69 (CH-5-pyrimidine); 126.31 (CH-5-triazole); 144.94 (CH-6); 149.32 (C-4-triazole); 154.27 (C-2); 160.43 (*t*BuOCON); 164.34 (C-2-pyrimidine); 167.03 (C-4); 171.55 (C-4-pyrimidine); 173.09 (C-6-pyrimidine); 182.83 (C-1-Arg).
^{31}P{^{1}H} NMR (202.4 MHz, D$_2$O): 2.95.
HR/MS (ESI$^-$) for $C_{29}H_{41}N_9O_{12}P^-$ 738.26178 [M-H]$^-$ calculated, found 738.26164 [M-H]$^-$.

Synthesis of (2R,3S,5R)-5-{[(5-((4-(2-(2-(((S)-4-((S)-2-((tert-butoxycarbonyl)amino) propanamido)-5-(((S)-1-carboxyethyl)amino)-5-oxopentyl)amino)-6-methylpyrimidin-4-yl)ethyl)-1H-1,2,3-triazol-1-yl]methyl}-2,4-dioxo-3,4-dihydropyrimidin-1[(2H)-yl)-3-hydroxytetrahydrofuran-2-yl]methyl hydrogen phosphate (dT^{HDOARA}MP)

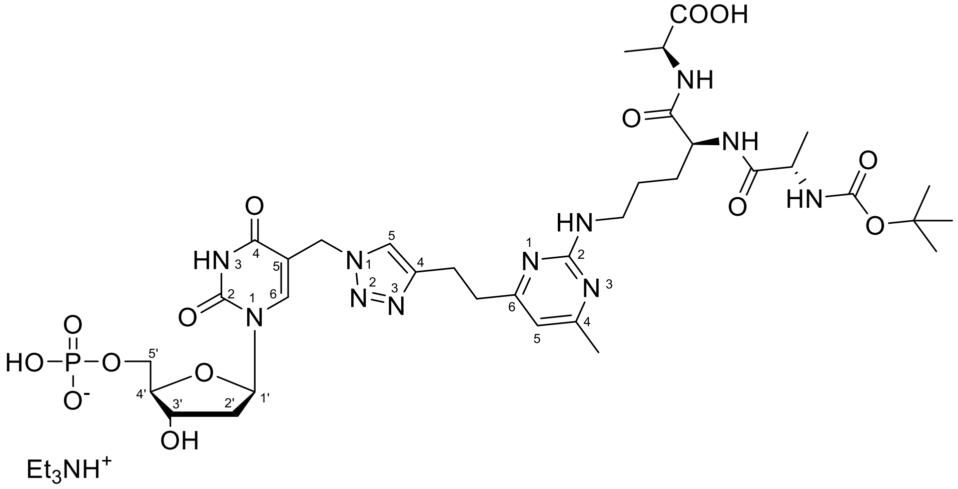

dT^{HDO}MP (10 mg 0.019 mmol) and N-Boc-L-AlaArgAla (24.9 mg, 0.059 mmol) were dissolved in $NaHCO_3$/Na_2CO_3 buffer (0.5M, pH 10. 240 μL) and the mixture was stirred 89 h at 25 °C. The product was purified on RP-HPLC with use of linear gradient of 0.1 M TEAB (Triethylammonium bicarbonate in H_2O) to 0.1 M TEAB in H_2O/MeOH (1:1) to MeOH as eluent. Several co-distillations with water followed by freeze drying gave the product as a yellow powder **dT^{HDOARA}MP** (6.1 mg, 35 % yield) Rf =0.75 (IPAV).

^{1}H NMR (500.0 MHz, CD_3OD): 1.30 (t, 9H, J_{vic} = 7.3, C**H$_3$**CH$_2$N); 1.32 (d, 3H, J_{vic} = 7.2, H-3-AlaBoc); 1.35 (d, 3H, J_{vic} = 7.1, H-3-Ala); 1.41 (bs, 9H, (CH$_3$)$_3$C); 1.62 – 1.75 (m, 3H, H-4,3b-Arg); 1.92 (m, 1H, H-3a-Arg); 2.23 (s, 3H, CH$_3$); 2.30 (ddd, 1H, J_{gem} = 13.8, $J_{2'b,1'}$ = 6.1, $J_{2'b,3'}$ = 3.2, H-2′b); 2.35 (ddd, 1H, J_{gem} = 13.8, $J_{2'a,1'}$ = 7.6, $J_{2'a,3'}$ = 6.0, H-2′a); 2.86 (bt, 2H, J_{vic} = 7.7, triazine-CH$_2$C**H$_2$**-pyrimidine); 3.02 – 3.07 (m, 2H, triazine-C**H$_2$**CH$_2$-pyrimidine); 3.18 (q, 6H, J_{vic} = 7.3, CH$_3$C**H$_2$**N); 3.39 (bt, 2H, $J_{5,4}$ = 6.5, H-5-Arg); 4.05 – 4.15 (m, 4H, H-4′,5′, H-2-AlaBoc); 4.19 (q, 1H, $J_{2,3}$ = 7.1, H-2-Ala); 4.40 (dd, 1H, $J_{2,3}$ = 8.6, 5.1, H-2-Arg); 4.54 (dt, 1H, $J_{3',2'}$ = 6.0, 3.2, $J_{3',4'}$ = 3.2, H-3′); 5.33, 5.37 (2 × d, 2 × 1H, J_{gem} = 14.5, CH$_2$N); 6.321 (s, 1H, H-5-pyrimidine); 6.324 (dd, 1H, $J_{1',2'}$ = 7.6, 6.1, H-1′); 7.82 (s, 1H, H-5-triazole); 8.46 (s, 1H, H-6).

^{13}C NMR (125.7 MHz, CD_3OD): 9.30 (**C**H$_3$CH$_2$N); 18.41 (CH$_3$-3-AlaBoc); 19.11 (CH$_3$-3-Ala); 23.67 (CH$_3$); 25.05 (triazine-**C**H$_2$CH$_2$-pyrimidine); 26.92 (CH$_2$-4-Arg); 28.72 ((**C**H$_3$)$_3$C); 30.67 (CH$_2$-3-Arg); 37.60 (triazine-CH$_2$**C**H$_2$-pyrimidine); 41.52 (CH$_2$-2′); 41.65 (CH$_2$-5-Arg); 47.74 (CH$_2$N); 47.85 (CH$_3$**C**H$_2$N); 51.50 (CH-2-Ala; 51.54 (CH-2-AlaBoc); 54.51 (CH-2-Arg); 65.80 (d, $J_{C,P}$ = 4.1, CH$_2$-5′); 72.70 (CH-3′); 80.56 ((CH$_3$)$_3$**C**); 86.91 (CH-1′); 88.14 (d, $J_{C,P}$ = 8.4, CH-4′); 109.76 (CH-5-pyrimidine); 109.79 (C-5); 124.05 (CH-5-triazole); 143.02 (CH-6); 147.92 (C-4-triazole); 152.13 (C-2); 157.64 (*t*BuOCON); 163.61 (C-2-pyrimidine); 164.73 (C-4); 169.07 (C-4-pyrimidine); 171.16 (C-6-pyrimidine); 173.01 (C-1-Arg); 175.85 (C-1-AlaBoc); 178.91 (C-1-Ala).

^{31}P{^{1}H} NMR (202.4 MHz, CD_3OD): 1.43.

HR/MS (ESI$^-$) for $C_{35}H_{51}N_{11}O_{14}P^-$ 880.33600 [M-H]$^-$ calculated, found 880.33526 [M-H]$^-$.

1.6. Cross-linking model reactions of dT^{HDO}MP with NH_2AlaGlyAla-OH , N-Ac-L-AlaLysAla-OH and N-Ac-AlaArgAla-OH

1.6.1 Synthesis of {[(Z)-6-(1-((1-((2R,4R,5R)-4-hydroxy-5-((phosphonooxy)methyl)tetrahydrofuran-2-yl)-2,4-dioxo-1,2,3,4-tetrahydropyrimidin-5-yl)methyl)-1H-1,2,3-triazol-4-yl)-4-oxohex-2-en-2-yl)]}-D-alanylglycyl-D-alanine (dT^{HDOAGA}MP)

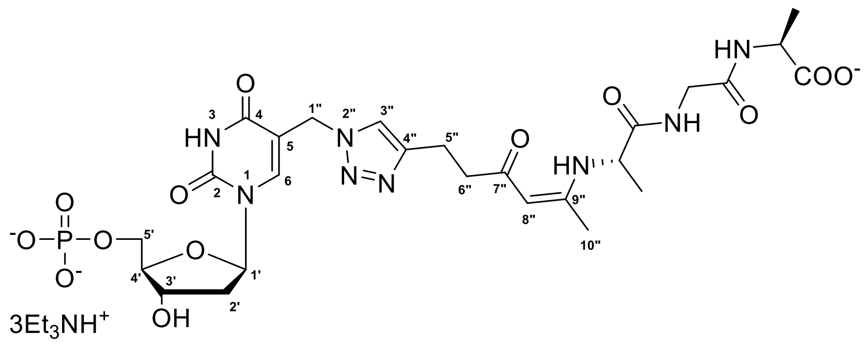

dT^{HDO}MP (10 mg 0.019 mmol) and NH_2AlaGlyAla-OH (13 mg, 0.059 mmol) were dissolved in $NaHCO_3/Na_2CO_3$ buffer (0.5 M, pH 10, 240 μL) and the mixture was stirred 18 h at 25 °C. The product was purified on RP-HPLC with use of linear gradient of 0.1 M TEAB (Triethylammonium bicarbonate in H_2O) to 0.1 M TEAB in H_2O/MeOH (1:1) to MeOH as eluent. Several co-distillations with water followed by freeze drying gave the product as a white powder **dT^{HDOAGA}MP** (1.85 mg, 13 % yield) Rf =0.62 (IPAV).

^{1}H NMR (500.0 MHz, D_2O, ref(*t*BuOH) = 1.24 ppm): 1.21 (t, 27H, J_{vic} = 7.3, C**H$_3$**CH$_2$N); 1.32 (d, 3H, $J_{3,2}$ = 7.3, H-3-Ala-3); 1.48 (d, 3H, $J_{3,2}$ = 7.0, H-3-Ala-1); 1.93 (s, 3H, H-10″); 2.33 (ddd, 1H, J_{gem} = 13.9, $J_{2'b,1'}$ = 6.6, $J_{2'b,3'}$ = 4.1, H-2′b); 2.36 (ddd, 1H, J_{gem} = 13.9, $J_{2'a,1'}$ = 7.5, $J_{2'a,3'}$ = 5.7, H-2′a); 2.52 – 2.65 (m, 2H, H-6″); 2.90 – 2.96 (m, 2H, H-5″); 3.06 (q, 18H, J_{vic} = 7.3, CH$_3$C**H$_2$**N); 3.91 (d, 1H, J_{gem} = 16.7, H-2b-Gly); 3.92 – 3.97 (m, 2H, H-5′); 3.98 (d, 1H, J_{gem} = 16.7, H-2a-Gly); 4.12 (m, 1H, H-4′); 4.15 (q, 1H, $J_{2,3}$ = 7.3, H-2-Ala-3); 4.38 (q, 1H, $J_{2,3}$ = 7.0, H-2-Ala-1); 4.56 (m, 1H, H-3′); 5.13 (s, 0.5H, H-8″); 5.23, 5.27 (2 × d, 2 × 1H, J_{gem} = 14.4, H-1″); 6.34 (dd, 1H, $J_{1',2'}$ = 7.5, 6.6, H-1′); 7.71 (s, 1H, H-3″); 8.09 (s, 1H, H-6).

^{13}C NMR (125.7 MHz, D_2O, ref(*t*BuOH) = 32.43 ppm): 11.39 (**C**H_3CH_2N); 20.34 (CH_3-3-Ala-3); 21.22, 21.28 (CH3-10″); 21.52 (CH_3-3-Ala-1); 24.46 (CH_2-5″); 41.67 (CH_2-2′); 43.15, 43.19 (CH_2-6′); 45.26 (CH_2-2-Gly); 49.25 (CH_3**C**H_2N); 50.78 (CH_2-1″); 53.71 (CH-2-Ala-3); 55.37 (CH-2-Ala-1); 66.51 (d, $J_{C,P}$ = 4.4, CH_2-5′); 74.11 (CH-3′); 88.16 (CH-1′); 88.65 (d, $J_{C,P}$ = 8.3, CH-4′); 99.17 (CH-8″); 111.81 (C-5); 125.79 (CH-3″); 143.93 (CH-6); 149.70 (C-4″); 161.22 (C-2); 169.35, 169.41 (C-9″); 172.76 (C-1-Gly); 176.07 (C-4); 178.31 (C-1-Ala-1); 182.78 (C-1-Ala-3); 200.83, 200.87 (C-7″).

$^{31}P\{^1H\}$ NMR (202.4 MHz, D_2O): 4.61.

HR/MS (ESI$^-$) for $C_{26}H_{36}N_8O_{13}P^-$ 699.21449 $[M-H]^-$ calculated, found 699.21411 $[M-H]^-$

1.6.2 Synthesis of N^2-(acetyl-L-alanyl)-N6-{[(Z)-6-(1-((1-((2R,4R,5R)-4-hydroxy-5-((phosphonooxy)methyl)tetrahydrofuran-2-yl)-2,4-dioxo-1,2,3,4-tetrahydropyrimidin-5-yl)methyl)-1H-1,2,3-triazol-4-yl)-4-oxohex-2-en-2-yl)]}-L-lysyl-L-alanine (dT^{HDOAKA}MP).

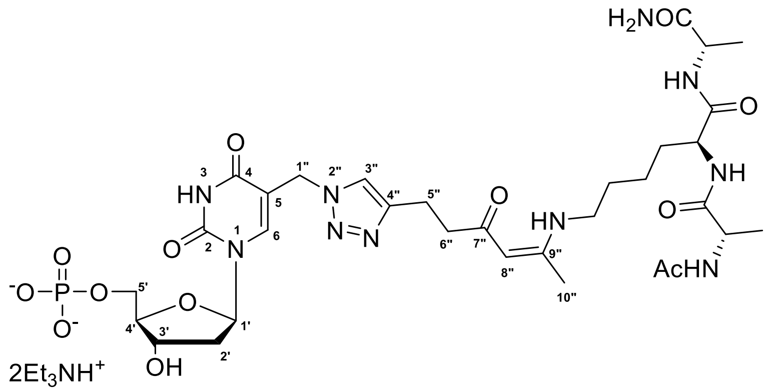

dT^{HDO}MP (10 mg 0.019 mmol) and N-Ac-L-AlaLysAla-OH (19.5 mg, 0.059 mmol) were dissolved in $NaHCO_3/Na_2CO_3$ buffer (0.5 M, pH 10, 240 μL) and the mixture was stirred 18 h at 25 °C. The product was purified on RP-HPLC with use of linear gradient of 0.1 M TEAB (Triethylammonium bicarbonate in H_2O) to 0.1 M TEAB in H_2O/MeOH (1:1) to MeOH as eluent. Several co-distillations with water followed by freeze drying gave the product as a yellow powder **dT^{HDOAKA}MP** (5.7 mg, 35 % yield) Rf =0.70 (IPAV).

^1H NMR (500.0 MHz, D_2O, ref(*t*BuOH) = 1.24 ppm): 1.22 (t, 18H, J_{vic} = 7.3, C**H$_3$**CH_2N); 1.31 (d, 3H, $J_{3,2}$ = 7.2, H-3-Ala); 1.35 (d, 3H, $J_{3,2}$ = 7.2, H-3-AlaAc); 1.36 – 1.48 (m, 2H, H-4-Lys); 1.55

– 1.66 (m, 2H, H-5-Lys); 1.73, 1.87 (2 × m, 2 × 1H, H-3-Lys); 1.95 (s, 3H, H-10″); 2.00 (s, 3H, CH_3CO); 2.30 – 2.40 (m, 2H, H-2′); 2.51 (t, 2H, $J_{6″,5″}$ = 7.5, H-6″); 2.91 (t, 2H, $J_{5″,6″}$ = 7.5, H-5″); 3.07 (q, 12H, J_{vic} = 7.3, $CH_3C\mathbf{H_2}N$); 3.32 (t, 2H, $J_{6,5}$ = 7.1, H-6-Lys); 3.94 (t, 2H, $J_{H,P}$ = $J_{5′,4′}$ = 4.8, H-5′); 4.10 (q, 1H, $J_{2,3}$ = 7.2, H-2-Ala); 4.12 (m, 1H, H-4′); 4.28 (q, 1H, $J_{2,3}$ = 7.2, H-2-AlaAc); 4.30 (dd, 1H, $J_{2,3}$ = 9.6, 4.7, H-2-Lys); 4.56 (m, 1H, H-3′); 5.25 (s, 2H, H-1″); 6.34 (t, 1H, $J_{1′,2′}$ = 6.8, H-1′); 7.70 (s, 1H, H-3″); 8.09 (s, 1H, H-6). H-8″ is completely exchanged by D

^{13}C NMR (125.7 MHz, D_2O, ref(*t*BuOH) = 32.43 ppm): 11.36 ($\mathbf{C}H_3CH_2N$); 19.49 (CH_3-3-AlaAc); 20.39 (CH_3-3-Ala); 21.07 (CH_3-10″); 24.41 ($\mathbf{C}H_3CO$); 24.73 (CH_2-5″); 25.18 (CH_2-4-Lys); 31.35 (CH_2-5-Lys); 33.25 (CH_2-3-Lys); 41.68 (CH_2-2′); 42.85 (CH_2-6″); 45.58 (CH_2-2-Lys); 49.27 ($CH_3\mathbf{C}H_2N$); 50.74 (CH_2-1″); 52.55 (CH-2-AlaAc); 53.79 (CH-2-Ala); 56.27 (CH-2-Lys); 66.50 (d, $J_{C,P}$ = 4.1, CH_2-5′); 74.11 (CH-3′); 88.14 (CH-1′); 88.66 (d, $J_{C,P}$ = 8.4, CH-4′); 97.42 (CH-8″); 111.82 (C-5); 125.77 (CH-3″); 143.95 (CH-6); 149.80 (C-4″); 161.08 (b, C-2); 171.27 (C-9″); 175.61 (C-1-Lys); 175.93 (b, C-4); 176.87 ($CH_3\mathbf{C}O$); 178.19 (C-1-AlaAc); 182.50 (C-1-Ala); 198.14 (C-7″).

^{31}P{^{1}H} NMR (202.4 MHz, D_2O): 4.61.

HR/MS (ESI$^-$) for $C_{32}H_{47}N_9O_{14}P^-$ 812.29856 [M-H]$^-$ calculated, found 812.29690 [M-H]$^-$

1.6.3 Synthesis of {[(S)-2-((S)-2-acetamidopropanamido)-5-((3-(2-(1-((1-((2R,4R,5R)-4-hydroxy-5-((phosphonooxy)methyl)tetrahydrofuran-2-yl)-2,4-dioxo-1,2,3,4-tetrahydropyrimidin-5-yl)methyl)-1H-1,2,3-triazol-4-yl)ethyl)-5-methylphenyl)amino)pentanoyl]}-L-alanine (dT^{HDOARA}MP).

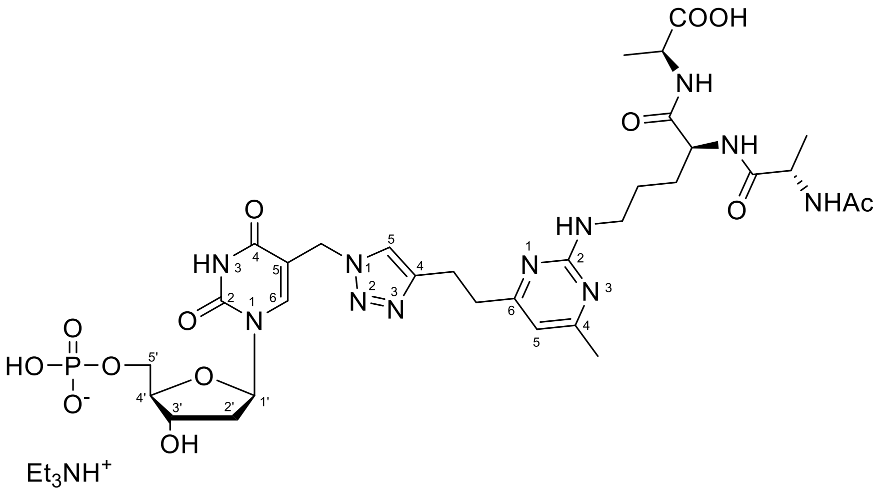

dT^{HDO}MP (10 mg 0.019 mmol) and N-Ac-L-AlaArgAla-OH (21.1 mg, 0.059 mmol) were dissolved in $NaHCO_3$/Na_2CO_3 buffer (0.5 M, pH 10, 240 µL) and the mixture was stirred 18 h at 25 °C. The product was purified on RP-HPLC with use of linear gradient of 0.1 M TEAB (Triethylammonium bicarbonate in H_2O) to 0.1 M TEAB in H_2O/MeOH (1:1) to MeOH as eluent. Several co-distillations with water followed by freeze drying gave the product as a yellow powder **dT^{HDOARA}MP** (2.1 mg, 12.7 % yield) Rf =0.75 (IPAV).

^{1}H NMR (600.1 MHz, D_2O, ref(*t*BuOH) = 1.24 ppm): 1.27 (t, 9H, J_{vic} = 7.3, C**H$_3$**CH$_2$N); 1.31 (d, 3H, J_{vic} = 7.2, H-3-Ala); 1.33 (d, 3H, J_{vic} = 7.2, H-3-AlaAc); 1.60 – 1.71 (m, 2H, H-4-Arg); 1.75, 1.91 (2 × m, 2 × 1H, H-3-Arg); 1.98 (s, 3H, CH_3CO); 2.22 (s, 3H, CH_3); 2.38 (ddd, 1H, J_{gem} = 14.1, $J_{2'b,1'}$ = 7.3, $J_{2'b,3'}$ = 6.0, H-2′b); 2.43 (ddd, 1H, J_{gem} = 14.1, $J_{2'a,1'}$ = 6.3, $J_{2'a,3'}$ = 3.5, H-2′a); 2.90 (bt, 2H, J_{vic} = 7.1, triazine-CH$_2$C**H$_2$**-pyrimidine); 3.05 – 3.10 (m, 2H, triazine-C**H$_2$**CH$_2$-pyrimidine); 3.20 (q, 6H, J_{vic} = 7.3, CH$_3$C**H$_2$**N); 3.34 (bt, 2H, $J_{5,4}$ = 6.8, H-5-Arg); 4.02 – 4.10 (m, 2H, H-5′); 4.11 (q, 1H, $J_{2,3}$ = 7.2, H-2-Ala); 4.21 (bm, 1H, H-4′); 4.27 (q, 1H, $J_{2,3}$ = 7.2, H-2-AlaAc); 4.34 (dd, 1H, $J_{2,3}$ = 9.0, 5.4, H-2-Arg); 4.59 (dt, 1H, $J_{3',2'}$ = 6.0, 3.5, $J_{3',4'}$ = 3.5, H-3′); 5.26, 5.30 (2 × d, 2 × 1H, J_{gem} = 14.9, CH_2N); 6.33 (s, 1H, H-5-pyrimidine); 6.33 (dd, 1H, $J_{1',2'}$ = 7.3, 6.3, H-1′); 7.64 (s, 1H, H-5-triazole); 8.27 (s, 1H, H-6).

^{13}C NMR (150.9 MHz, ref(*t*BuOH) = 32.43 ppm): 11.06 (**C**H$_3$CH$_2$N); 19.50 (CH$_3$-3-Ala); 20.37 (CH$_3$-3-AlaAc); 24.37 (**C**H$_3$CO); 25.05 (CH_3); 26.24 (triazine-**C**H$_2$CH$_2$-pyrimidine); 27.68 (CH$_2$-4-Arg); 31.12 (CH$_2$-3-Arg); 38.50 (triazine-CH$_2$**C**H$_2$-pyrimidine); 42.07 (CH$_2$-2′); 43.18 (CH$_2$-5-Arg); 49.45 (CH_2N); 49.52 (CH$_3$**C**H$_2$N); 52.44 (CH-2-AlaAc); 53.77 (CH-2-Ala); 56.22 (CH-2-Arg); 67.25 (d, $J_{C,P}$ = 4.2, CH$_2$-5′); 73.89 (CH-3′); 88.54 (CH-1′); 88.83 (d, $J_{C,P}$ = 8.6, CH-4′);

111.59 (C-5); 112.66 (CH-5-pyrimidine); 126.21 (CH-5-triazole); 144.77 (CH-6); 149.32 (C-4-triazole); 154.21 (C-2); 163.33 (C-2-pyrimidine); 166.96 (C-4); 170.99 (C-4-pyrimidine); 173.28 (C-6-pyrimidine); 175.43 (C-1-Arg); 176.77 (CH_3**C**O); 178.05 (C-1-AlaAc); 182.36 (C-1-Ala).
$^{31}P\{^{1}H\}$ NMR (202.4 MHz, D_2O): 1.60.
HR/MS (ESI^-) for $C_{32}H_{45}N_{11}O_{13}P^-$ 822.29414 $[M-H]^-$ calculated, found 822.29294 $[M-H]^-$.

1.7 Stability studies of the conjugates dT^{HDOAGA}MP, dT^{HDOAKA}MP, dT^{HDOARA}MP in H_2O.

The isolated compounds**, dT^{HDOAGA}MP**, **dT^{HDOAGA}MP**, **dT^{HDOAGA}**MP were stirred in 1 mL of H_2O at 25°C for 20 h. Subsequently they were diluted and analysed by HPLC using Column X-Bridge Prep. RP 18, 5 µm DBD, 19x150 mm with use of linear gradient of 0.1 M TEAB (Triethylammonium bicarbonate in H_2O) to 0.1 M TEAB in H_2O/MeOH (1:1) to MeOH as eluent. In the case of **dT^{HDOAGA}MP** and **dT^{HDOAKA}MP** hydrolysis to the starting material **dT^{HDO}MP** was observed (see Scheme S3 and Figure S41-43 A), whereas only 23% (430 mg) of **dT^{HDOAGA}MP** or 13% (740 mg) of **dT^{HDOAKA}MP** were recovered. In the case of **dT^{HDOARA}MP** no hydrolysis was observed (see SI section 5 Figure S45) and 95% (2 mg) of **dT^{HDOARA}MP** was recovered. The compounds were analysed by NMR spectroscopy and ESI- mass spectrometry (See Scheme S3 and Figure S41-43-45 B).

2. Experimental section- biochemistry part

General remarks for the biochemical part

All gels were analysed by fluorescence imaging using Typhoon FLA 9500 (GE Healthcare). Mass spectra of oligonucleotides were measured on UltrafleXtreme MALDI-TOF/TOF (Bruker) mass spectrometer with 1 kHz smartbeam II laser. UV-Vis spectra were measured at room temperature on NanoDrop1000 (ThermoScientific). Fluorescence was measured on a Fluoromax 4 spectrofluorimeter (HORIBA Scientific). Samples were concentrated on CentriVap Vacuum Concentrator system (Labconco). Synthetic oligonucleotides (primers, templates and biotinylated templates; for sequences see Table S1) were purchased from Generi Biotech (Czech Republic).

Natural nucleoside triphosphates (dATP, dGTP, dTTP, dCTP) were purchased from Thermo Scientific. BSA and histone human recombinant proteins (H2A, H2B, H3.1 and H4) were purchased from New England Biolabs. KOD XL DNA polymerase and corresponding polymerase reaction buffer from Merck Millipore, streptavidin magnetic particles were obtained from Sigma Aldrich (Merck), QIAquick® Nucleotide Removal Kit QIAGEN (Biotech, Czech Republic). Milli-Q water was used for all experiments. PAGE stop solution used after PEX reactions contains: 95% [v/v] formamide, 0.5 mM EDTA, 0.025% [w/v] bromophenol blue, 0.025% [w/v] xylene cyanol, 0.025% SDS. VPS loading buffer used for protein SDS gels contains: 0.05 M TRIS (pH 6.8), 17% glycerol, 16 mM mercaptoethanol, 3.5% SDS, and bromophenol blue. PageBlue™ protein staining solution was obtained from Thermofisher Scientific. Samples after PEX reaction were analyzed by either 12.5% or 20% PAGE (Acrylamide/bisacrylamide 19:1) under denaturing conditions (1 h, 50 °C, 1xTBE buffer). Samples after conjugation with Histones were analyzed by 17.5% SDS denaturing PAGE (acrylamide/methylenebisacrylamide 29:1; 1.92 M glycine, 0.25 M Tris, 0.1% SDS- 230 V/1 h). Other chemicals were of analytical grade.

2.1. Lists of sequences of primers, templates and oligonucleotides used in this study.

Table S1. List of sequences of primers and templates used in this study.

**Name**	**Sequence (5’→3’)**	**Length**
**Prim ^{A}**	5\`-CATGGGCGGCATGGG-3\`	15 nt
**Prim $^{A\ a}$**	5\`-CATGGGCGGCATGGG-3\`	15 nt
**Prim ^{B}**	5\`-TCAAGAGACATGCCT-3\`	15 nt
**Prim $^{B\ a}$**	5\`-TCAAGAGACATGCCT-3\`	15 nt
**Temp^{19_1T}**	5\`-CCCACCCATGCCGCCCATG-3\`	19 nt
**Temp$^{19_1T\ b}$**	5\`-CCCACCCATGCCGCCCATG-3\`	19 nt
**Temp^{30_1T}**	5\`-TTGTTGGGCATGTCTAGGCATGTCTCTTGA-3\`	30 nt
**Temp$^{30_1T\ b}$**	5\`-TTGTTGGGCATGTCTAGGCATGTCTCTTGA-3\`	30 nt
**Temp^{30_2T}**	5\`-TTTTTAGGCATGTCTAGGCATGTCTCTTGA-3\`	30 nt
**Temp$^{30_2T\ b}$**	5\`-TTTTTAGGCATGTCTAGGCATGTCTCTTGA-3\`	30 nt

**Temp^{31_4T}**	5`-CTAGCATGAGCTCAGTCCCATGCCGCCCATG-3`	31 nt
**Temp$^{31_4T\ b}$**	5`-CTAGCATGAGCTCAGTCCCATGCCGCCCATG-3`	31 nt

[a]5`-(6-FAM) labelled, [b] 5`-biotinylated

Table S2. List of oligonucleotides used in this study

**Name**	**Sequence (3→5`)**	**Length**
**19ON_T^{HDO}**	3`-GGG**T$^{HDO}$**GGGTACGGCGGGTAC-5`	19 nt
**19ON_T^{PDO}**	3`-GGG**T$^{PDO}$**GGGTACGGCGGGTAC-5`	19 nt
**30ON_T^{HDO}**	3`-AACAACCCG**T$^{HDO}$**ACAGATCCGTACAGAGAACT-5`	30 nt
**30ON_T^{PDO}**	3`-AACAACCCG**T$^{PDO}$**ACAGATCCGTACAGAGAACT-5`	30 nt
**19DNA_T$^{HDO\ a,b}$**	3`-GGG**T$^{HDO}$**GGGTACGGCGGGTAC-5`	19 nt
**25DNA_T$^{HDO\ a,b}$**	3`-CCGCCCG**T$^{HDO}$**ACAGACCTGTACAGAGT-5`	25 nt
**30DNA_T$^{HDO\ a,b}$**	3`-AACAACCCG**T$^{HDO}$**ACAGATCCGTACAGAGAACT-5`	30 nt
**30DNA_T$^{PDO\ a,b}$**	3`-AACAACCCG**T$^{PDO}$**ACAGATCCGTACAGAGAACT-5`	30 nt
**30DNA_2T$^{HDO\ a,b}$**	3`-AAAA**T^{HDO}**CCCG**T^{HDO}**ACAGATCCGTACAGAGAACT-5	30 nt

[a]5`-(6-FAM)-labelled, [b] 5`-biotinylated, ON^{X}-single stranded DNA, DNA^{X}- double stranded DNA.

2.2. Enzymatic synthesis of HDO- and PDO-modified DNA

2.2.1. Single incorporation of dT$^{HDO/PDO}$TP using 19-mer template-Analytical scale

The reaction mixture (20 μL) contained primer **Prim ^{Aa}** (4 μM, 1 μL), template **Temp^{19_1T}** (4 μM, 1.5 μL), KOD XL DNA polymerase (0.25 U/μL, 0.5 μL), natural dGTP (0.4 mM, 0.5 μL), either natural or modified dTTP (0.4 mM, 1 μL) in enzyme reaction buffer (10X, 2 μL). The reaction mixture was incubated for 30 min at 60 °C in a thermal cycler. The PEX reaction was stopped by addition of PAGE stop solution (20 μL) and heated for 5 min at 95 °C. Samples were separated with denaturing PAGE and visualized using fluorescence imaging (Figure S1A,E).

2.2.2. Single incorporation of dT$^{HDO/PDO}$TP using 30-mer template-Analytical scale

The reaction mixture (20 μL) contained primer **Prim ^{Ba}** (3 μM, 1 μL), template **Temp^{30_1T}** (3 μM, 1.5 μL), KOD XL DNA polymerase (0.25 U/μL, 0.5 μL), natural dNTPs (dGTP, dATP, dCTP;

4 mM, 0.5 µL), either natural or modified dTTP (0.4 mM, 1 µL) in enzyme reaction buffer (10 X, 2 µL). The reaction mixture was incubated for 30 min at 60°C in a thermal cycler. The PEX reaction was stopped by addition of PAGE stop solution (20 µL) and heated for 5 min at 95 °C. Sample were separated with denaturing 12.5% PAGE and visualized using fluorescence imaging (Figure S1B/F).

2.2.3. Single incorporation of two $dT^{HDO}TP$ using 30-mer template-Analytical scale

The reaction mixture (20 µL) contained primer **Prim ^{Ba}** (3 µM, 1 µL), template **Temp^{30_2T}** (3 µM, 1.5 µL), KOD XL DNA polymerase (0.25 U/µL, 0.25 µL), natural dNTPs (dGTP, dATP, dCTP; 4 mM, 0.5 µL), either natural or modified dTTP (0.4 mM, 1.5 µL) in enzyme reaction buffer (10X, 2 µL). The reaction mixture was incubated for 30 min at 60 °C in a thermal cycler. The PEX reaction was stopped by addition of PAGE stop solution (20 µL) and heated for 5 min at 95°C. Sample were separated with denaturing 12.5 % PAGE and visualized using fluorescence imaging (Figure S1C).

2.2.4. Single incorporation of four $dT^{HDO}TP$ using 31-mer template-Analytical scale

The reaction mixture (20 µL) contained **Prim^{Aa}** (4 µM, 1 µL), template **Temp^{31_4C}** (4 µM, 1.5 µL), KOD XL DNA polymerase (0.25 U/µL, 0.6 µL), natural dNTPs (dGTP, dATP, dCTP; 4 mM, 1µL), either natural or modified dTTP (4 mM, 0.6 µL) in enzyme reaction buffer (10X, 2 µL). The reaction mixture was incubated for 30 min at 60° C in a thermal cycler. The PEX reaction was stopped by addition of PAGE stop solution (20 µL) and heated for 5 min at 95°C. Sample were separated with denaturing 12.5 % PAGE and visualized using fluorescence imaging (Figure S1D).

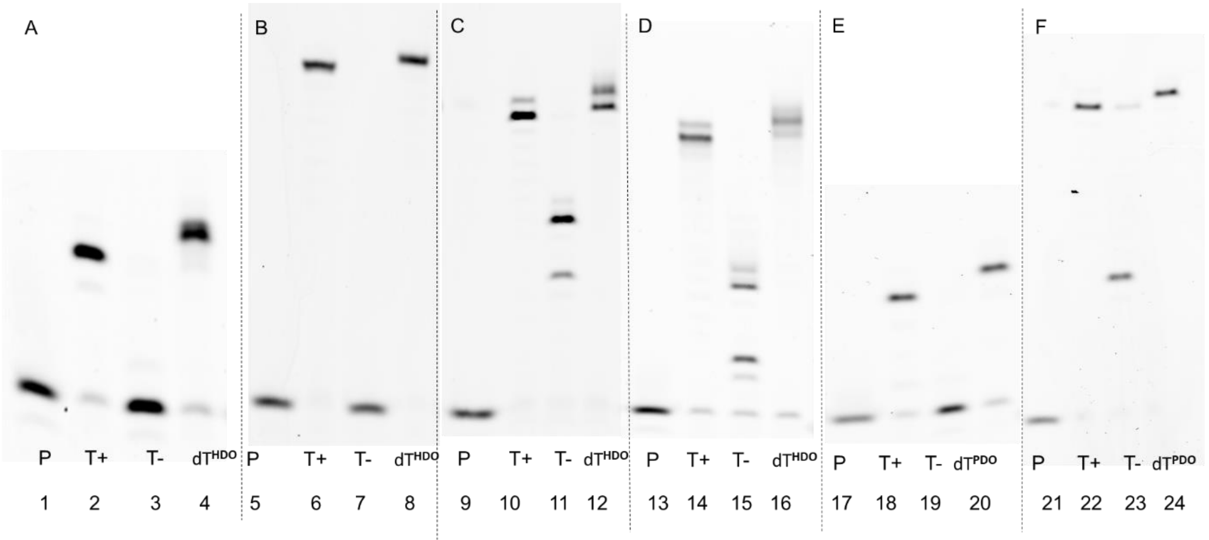

Figure S1: Denaturing PAGE analysis of PEX using KOD XL DNA polymerase and template **Temp**$^{\mathbf{19\_1T}}$ (lanes 2-4, 18-20), template **Temp**$^{\mathbf{30\_1T}}$ (lanes 6-8, 22-24), template **Temp**$^{\mathbf{30\_2T}}$ (lanes 10-12) and template **Temp**$^{\mathbf{30\_4T}}$ (lanes 14-16).

(P): primer **Prim**$^{\mathbf{Aa}}$, lane 1; (P): primer **Prim**$^{\mathbf{Ba}}$, lanes 5,9; (T+): natural dNTP, lanes 2,6,10,14.18,21; (T-): negative control without dTTP, lanes 3,7,11,15,19,23; (dT^{HDO}): modified DNA containing **dT**$^{\mathbf{HDO}}$**TP**, dGTP, dCTP, dATP, lanes 4,8,12,16; (dT^{PDO}): modified DNA containing **dT**$^{\mathbf{PDO}}$**TP**, dGTP, dCTP, dATP, lanes 20, 24.

2.2.5. Single incorporation of dT$^{HDO/PDO}$TP using 19-mer template-Semipreparative scale

The reaction mixture (50 μL) contained KOD XL DNA polymerase (0.25 U/μL, 0.85 μL), primer **Prim** $^{\mathbf{A}}$ or primer **Prim** $^{\mathbf{Aa}}$ (100 μM, 1.7 μL), template **Temp**$^{\mathbf{19\_1T}}$ or template **Temp**$^{\mathbf{19\_1T\ b}}$ (100 μM, 2.55 μL), dNTPs (either natural or modified; 4 mM, 2.38 μL each) in KOD XL reaction buffer (10X, 5 μL). The reaction mixture was incubated in a thermal cycler for 40 min at 60 °C and stopped by cooling to 4° C.

A) Biotinylated PEX products were purified using DB Streptavidin magneto-separation procedure and then analysed by MALDI-TOF mass spectrometry. (See Table S2 for copies of mass spectra see SI part 4).

B) 5`-(6-FAM)-labelled PEX products were purified using QIAquick nucleotide removal kit using the standard procedure.

2.2.6. Single incorporation of dT$^{HDO/PDO}$TP using 30-mer template-Semipreparative scale

The reaction mixture (50 μL) contained KOD XL DNA polymerase (0.25 U/μL, 0.3 μL), primer **Prim** ^{B} or primer **Prim** ^{Ba} (100 μM, 2 μL), template **Temp**$^{30\_1T}$ or template **Temp**$^{30\_1T\,b}$ (100 μM, 2 μL), dNTPs (either natural or modified; 4 mM, 0.375 μL each) in KOD XL reaction buffer (10X, 5 μL). The reaction mixture was incubated in a thermal cycler for 40 min at 60 °C and stopped by cooling to 4 °C.

A) Biotinylated PEX products were purified using DB Streptavidin magneto separation procedure and then analysed by MALDI-TOF mass spectrometry. (See Table S2 for copies of mass spectra see SI part 4).

B) 5`-(6-FAM)-labelled PEX products were purified using QIAquick Nucleotide Removal Kit Nucleotide removal kit using the standard procedure.

2.2.7. DB Streptavidin magneto-separation procedure:

Streptavidin particles (Roche; 50 μL) were washed with binding buffer (3 × 200 μL; 10 mM Tris, 1 mM EDTA, 100 mM NaCl, pH 7.5). The PEX solution (50 μL) was mixed with binding buffer (200 μL) and incubated for 30 min at 15°C and 1400 rpm. The magnetic beads were collected on a magnet (DynaMagTM-2, Invitrogen), and washed with wash buffer (3 × 300 μL; 10 mM Tris, 1 mM EDTA, 500 mM NaCl, pH 7.5) and water (4 × 300 μL). Then water (50 μL) was added and the sample was denatured for 2 min at 900 rpm and 75 °C. The beads were collected on a magnet and the solution was transferred into a clean vial. The product was evaporated to dryness, then dissolved in water and analysed by MALDI-TOF mass spectrometry.

2.2.8. Table S3. MALDI data of dT^{HDO}-modified oligonucleotides.

ON	Mw(calc.)/[Da]	Mw(found) /[Da] [M+H]$^+$	Δ
**19DNA_T$^{HDO\ b}$**	6146.0	6147.0	1.0
**19DNA_T$^{HDO\ a}$**	6682.0	6682.9	0.9
**19DNA_T$^{PDO\ b}$**	6146.0	6146.0	0
**30DNA_T$^{HDO\ b}$**	9342.9	9343.9	1.0
**30DNA_T$^{HDO\ a}$**	9878.1	9879.2	1.1
**30DNA_T$^{PDO\ b}$**	9342.9	9344.1	1.2
**30DNA_2T$^{HDO\ a}$**	10096.1	10098.4	2.3
**31DNA_4T$^{HDO\ a}$**	10870.6	10875.5	4.9

[a] 5`-(6-FAM)-labelled, [b] 5`-biotinylated, ON^X-single stranded DNA, DNA^X- double stranded DNA

2.3. Bio-conjugation reactions

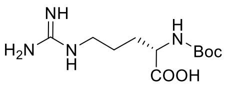

N-L-Boc-**Arg**-OH
N-L-Boc-**R**-OH
N-Boc**Arg**

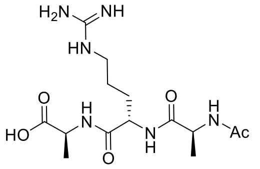

N-Ac-L-Ala**Arg**Ala-OH
N-Ac-L-A**R**A-OH
Ac-Ala**Arg**Ala-OH

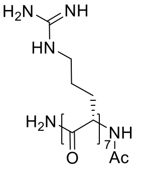

N-Ac-L-(**Arg**)$_7$-NH$_2$
N-Ac-L-(**R**)$_7$-NH$_2$
Ac-(**Arg**)$_7$ –NH$_2$

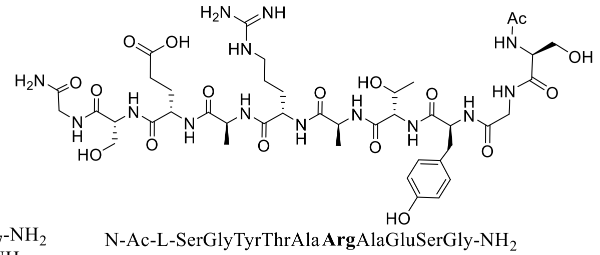

N-Ac-L-SerGlyTyrThrAla **Arg**AlaGluSerGly-NH$_2$
N-Ac-L-SGYTA **R**AESG-NH$_2$
Ac-SGYTA **Arg**AESG-NH$_2$

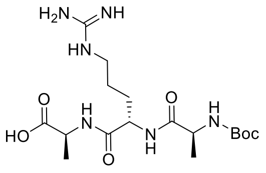

N-Boc-L-Ala**Arg**Ala-OH
N-Boc-L-A**R**A-OH
Boc-Ala**Arg**Ala-OH

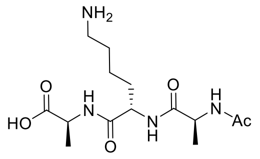

N-Ac-L-AlaLysAla-OH
N-Ac-L-AKA-OH
Ac-AlaLysAla-OH

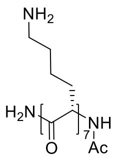

N-Ac-L-(**Lys**)$_7$-NH$_2$
N-Ac-L-(**K**)$_7$-NH$_2$

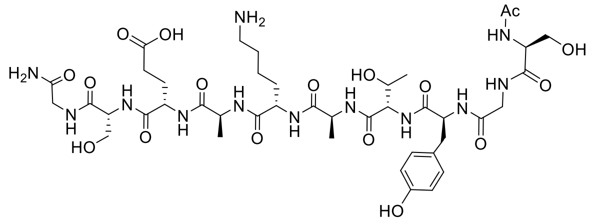

N-Ac-L-SerGlyTyrThrAla **Lys**AlaGluSerGly-NH$_2$
N-Ac-L-SGYTA **K**AESG-NH$_2$
Ac-SGYTA **Lys**AESG-NH$_2$

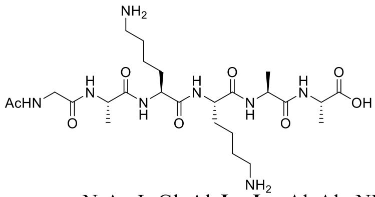

N-Ac-L-GlyAla**LysLys** AlaAla-NH$_2$
N-Ac-GlyAla**LysLys** AlaAla-NH$_2$
Ac-GA**KK**AA-NH$_2$

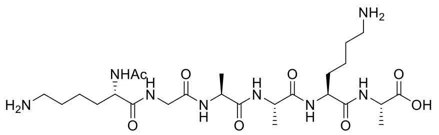

N-Ac-**Lys**GlyAlaAla**Lys**Ala-NH$_2$
N-Ac-L-**Lys**GlyAlaAla**Lys**Ala-NH$_2$
Ac-**K**GAA**K**A-NH$_2$

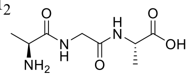

NH$_2$-Ala-Gly-Ala-OH
NH$_2$-L-Ala-Gly-Ala-OH
NH$_2$-AGA-OH

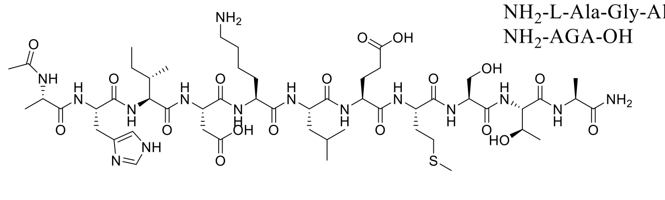

N-Ac-AlaHisIleAspLysLeuGluMetSerThrAla-NH$_2$
N-Ac-L-AlaHisIleAspLysLeuGluMetSerThrAla-NH$_2$
Ac-AHIDKLEMSTA-NH$_2$

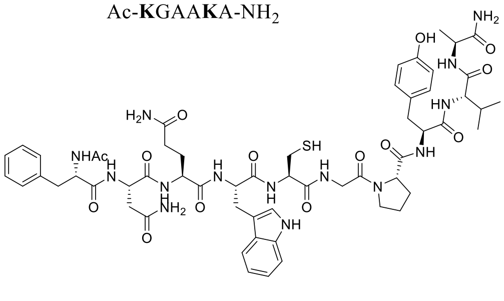

N-Ac-PheAsnGlnTrpCysGlyProTyrValAla-N H$_2$
N-Ac-L-PheAsnGlnTrpCysGlyProTyrValAla-N H$_2$
Ac-FNEWCGPYVA-NH$_2$

2.3.1. Scheme S4. Structures of peptides used in this study.

2.3.2. Reaction of 19DNA_T^{HDO} with N-Boc-L-Arginine-PAGE analysis.

19DNA_T$^{HDO\ a}$ was prepared by PEX in semi-preparative scale as described above (Section 2.2). The reaction mixture (20µL) containing **19DNA_T$^{HDO\ a}$** (4.3 µM) was incubated with N-Boc-L-Arg-OH (11mM, 22mM and 250 mM) in $NaHCO_3$ buffer (0.5 M, pH 10) for 18 h at 25°C or 37°C in a thermal cycler. Subsequently quenched by addition of PAGE stop solution (20 µL) and heated 5 min at 95 °C. Samples were analysed by 20 % denaturing PAGE and visualized using florescence imaging (Figure S2).

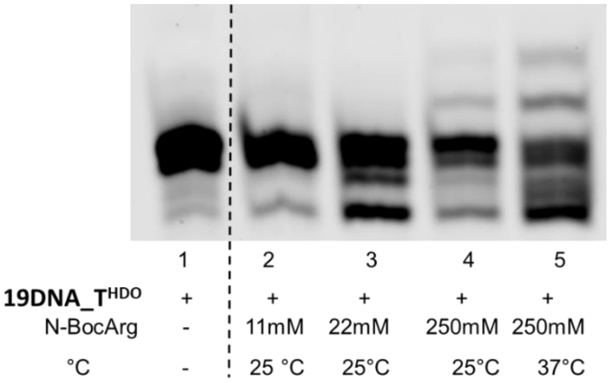

Figure S2. Denaturing PAGE analysis of conjugation of **19DNA_T^{HDO}** (4.3 µM, lane 1) with N-BocArg-OH (11 mM, lane 2; 22 mM, lane 3 and 250 mM lane 4,5) at 25°C (lane 2,3,4) and at 37°C (lane 5) in $NaHCO_3$ buffer (0.5 M, pH 10) for 18 h.

2.3.3. Reaction of 19DNA_T^{HDO} and 30DNA_T^{HDO} with N-Boc-L-Arginine and arginine containing peptides- PAGE analysis.

19DNA_T$^{HDO\ a}$ or **30DNA_T$^{HDO\ a}$** were prepared by PEX in semi-preparative scale as described above (Section 2.2). The reaction mixture (20 µL) containing **19DNA_T^{HDOa}** or **30DNA_T^{HDOa}** (0.83 µM) and N-Boc-L-Arg-OH, tripeptide or decapeptide (250 mM) each in $NaHCO_3$ buffer (0.5 M, pH 10) was incubated for 18 h at 37 °C in a thermal cycler and subsequently quenched by addition of PAGE stop solution (20 µL) and heated 5 min at 95 °C. Samples were analysed by 20 % denaturing PAGE and visualized using florescence imaging (Figure S3 A, B).

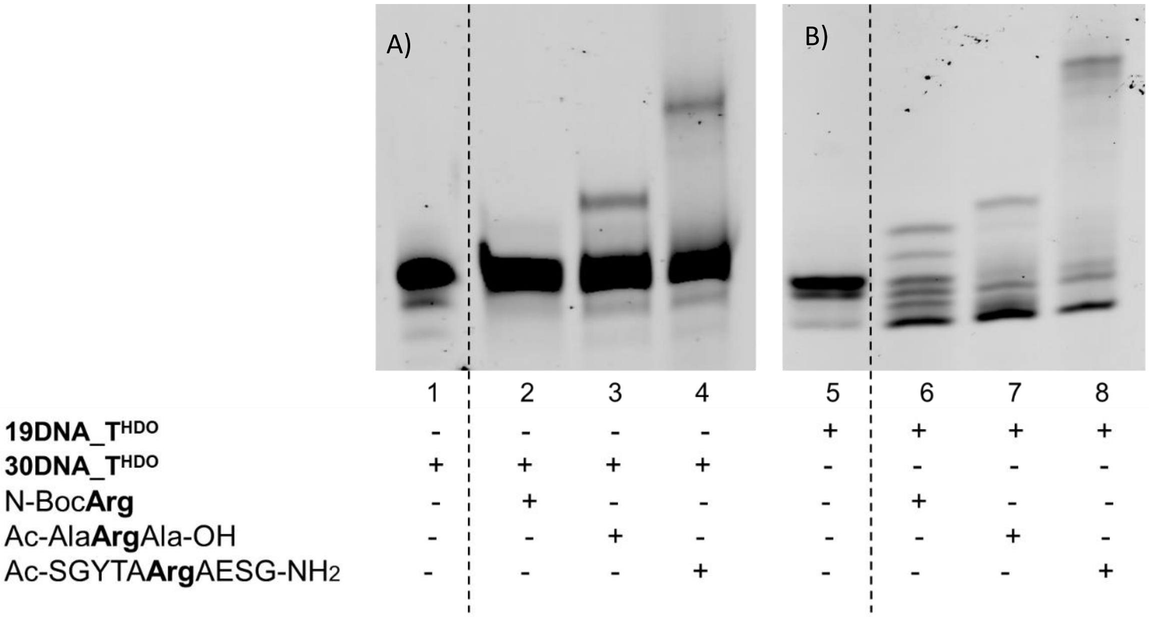

Figure S3. Denaturing PAGE analysis. A) Conjugation of **30DNA_T^{HDO}** [a] (0.83μM, lane 1) with N-Boc-Arginine (250 mM, lane 2), with tripeptide (250 mM, lane 3) and with decapeptide (250 mM, lane 4) in $NaHCO_3$ buffer (0.5 M, pH 10) at 37°C, for 18 h. B) Conjugation of **19DNA_T^{HDO}** [a] (0.83 μM, lane 5) with N-Boc-Arginine (250 mM, lane 6), with tripeptide (250 mM, lane 7) and with decapeptide (250 mM, lane 8) in $NaHCO_3$ buffer (0.5 M, pH 10) at 37° C for 18 h.

2.3.4 19DNA_T^{HDO} and 30DNA_T^{HDO} with N-Boc-L-Arginine and arginine containing peptides- MALDI-TOF analysis.

19DNA_T^{HDO} and **30DNA_T^{HDO}** were prepared by PEX in semi preparative scale as described above (Section 2.2). The product was purified on QIAquick Nucletide Removal Kit according the standard protocol. The reaction mixture (20 μL), containing **19DNA_T^{HDO}** [b]**, 30DNA_T^{HDO}** [b] (3.50 μM) and N-Boc-L-Arg-OH, tripeptide or decapeptide (250 mM) each in $NaHCO_3$ buffer (pH 10, 0.5 M), was heated at 37 °C for 18 h. Subsequently was purified by magneto separation as described above and analysed by MALDI-TOF mass spectrometry (See Table.S4 and for copies of mass spectra see SI part 4).

2.3.5. Table S4 MALDI data of arginine-modified oligonucleotides adducts.

ON	Mw(calc.)/[Da]	Mw(found)/[Da] $[M+H]^+$	Δ
**19ON_T^{HDOArg}**	6383.1	6386.7	3.6
**19ON_T$^{HDO\ ARA}$**	6467.1	6473.6	6.5
**19ON_T$^{HDO\ 10pept}$**	7147.4	7154.6	7.2
**30ON_T^{HDOArg}**	9580.2	9583.6	3.4
**30ON_T$^{HDO\ 10pept}$**	10344.5	10347.4	2.9

, ON^X-single stranded DNA

2.4. Reactions of 30DNA_T$^{HDO/PDO}$ with lysine and arginine containing peptides- PAGE analysis

2.4.1. Reaction with heptapeptides as Ac-(Lys)$_7$-NH$_2$ and Ac-(Arg)$_7$- NH$_2$.

30DNA_$^{Natural\ a}$, 30DNA_T$^{HDO\ a}$ and **30DNA_T$^{PDO\ a}$** were prepared by PEX in semi-preparative scale as described above (Section 2.2). The product was purified on QIAquick Nucleotide Removal Kit eluted with water and lyophilized. The reaction mixture (20 μL) contained **30DNA_$^{Natural\ a}$** or **30DNA_T$^{HDO\ a}$** or **30DNA_T$^{PDO\ a}$** (0.83 μM) and Ac- (Lys)$_7$- NH$_2$ (250 mM) or Ac-(Arg)$_7$-NH$_2$ (250 mM) in NaHCO$_3$ buffer (0.5 M, pH 10) and was incubated for18 h at 37 °C in a thermal cycler and subsequently quenched by addition of PAGE stop solution (20 μL) and heated 5 min at 95 °C. Samples were separated by denaturing 20 % PAGE (TBE 1X, 30 mA/42 mA, 1.5 h) and visualized with florescence imaging. (Figure S4).

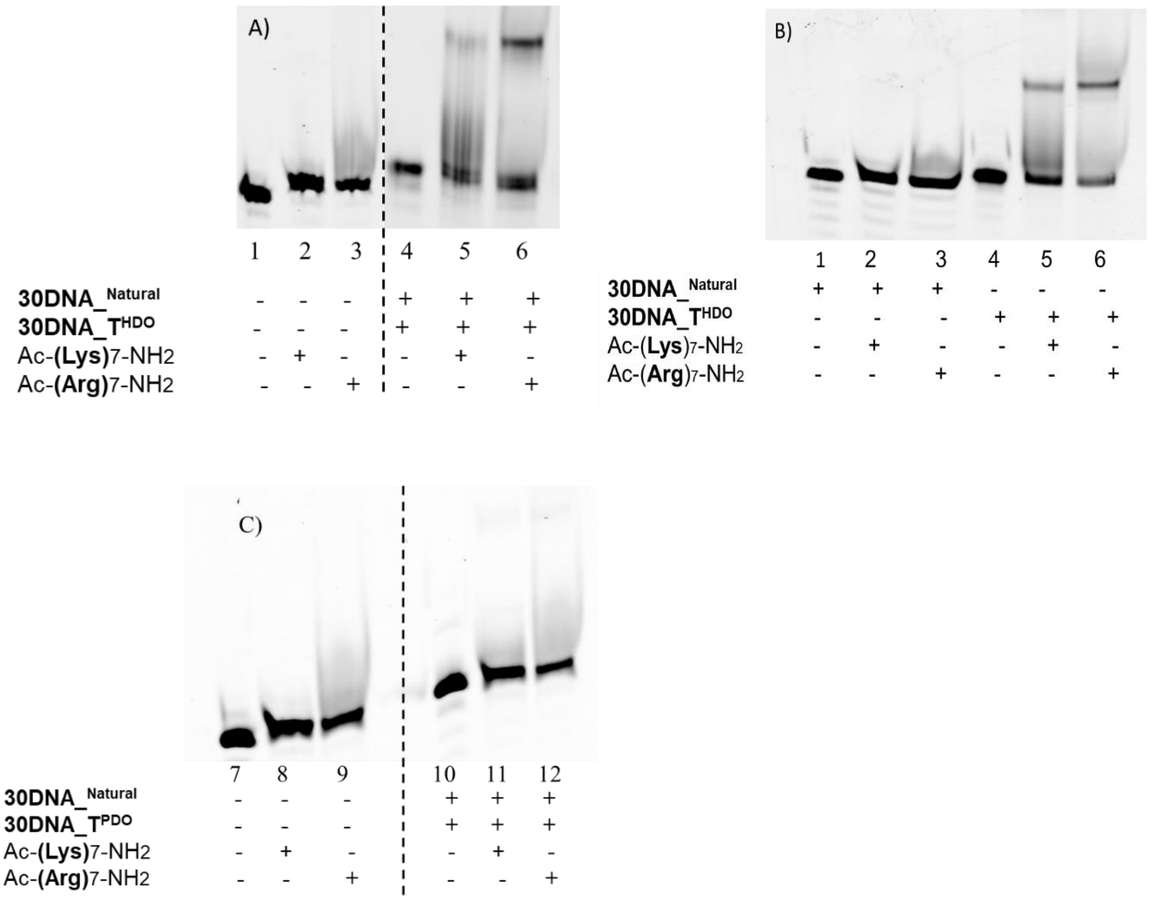

Figure S4. Denaturing PAGE gel analysis comparing conjugations of A) **30DNA_$^{\mathbf{Natural}}$** (0.83 μM, lanes 1,2,3) or **30DNA_T$^{\mathbf{HDO\ a}}$** (0.83 μM, lanes 4,5,6) with heptapeptide Ac-(Lys)$_7$-NH$_2$ (250 mM; lanes 2,5) and with heptapeptide Ac-(Arg)$_7$-NH$_2$ (250 mM, lanes 3,6) in NaHCO$_3$ buffer (0.5 M, pH 10) at 37 °C for 18 h (Run 42 mA). B) **30DNA_$^{\mathbf{Natural}}$** (0.83 μM, lanes 1,2,3) or **30DNA_T$^{\mathbf{HDO}}$** (0.83 μM, lanes 4,5,6) with heptapeptide Ac-(Lys)$_7$-NH$_2$ (250 mM, lanes 2,5) and with heptapeptide Ac-(Arg)$_7$-NH$_2$ (250 mM, lane 3,6) in NaHCO$_3$ buffer (0.5 M pH 10) at 37 °C for 18 h (Run 30 mA) and C) **30DNA_$^{\mathbf{Natural}}$** (0.83 μM, lanes 7,8,9) or **30DNA_T$^{\mathbf{PDO}}$** (0.83 μM; lanes 10,11,12) with heptapeptide Ac-(Lys)$_7$-NH$_2$ (250 mM, lanes 8,11) and with heptapeptide Ac-(Arg)$_7$-NH$_2$ (250 mM, lanes 9,12) in NaHCO$_3$ buffer (0.5 M, pH 10) at 37 °C for 18 h (Run 42 mA).

2.4.2. Reaction with tripeptides as Ac-L-AlaLysAla-OH and Ac-L-AlaArgAla-OH.

30DNA_T$^{\mathbf{HDO\ a}}$ or **30DNA_T$^{\mathbf{PDOa}}$** were prepared by PEX in semi-preparative scale as described above (Section 2.2). The product was purified on QIAquick Nucleotide Removal Kit eluted with water and lyophilized. The reaction mixture (20 μL) contained **30DNA_T$^{\mathbf{HDO\ a}}$** or **30DNA_T$^{\mathbf{PDOa}}$** (0.83 μM) and Ac-L-AlaLysAla-OH (250 mM) or Ac-L-AlaArgAla-OH (250 mM) in NaHCO$_3$ buffer (0.5 M, pH 10) and was incubated for18 h at 37 °C in a thermal cycler and subsequently quenched by addition of PAGE stop solution (20 μL) and heated 5 min at 95 °C. Samples were separated by denaturing 20 % PAGE (TBE 1X, 42 mA, 1.5 h) and visualized with florescence imaging. (Figure S5).

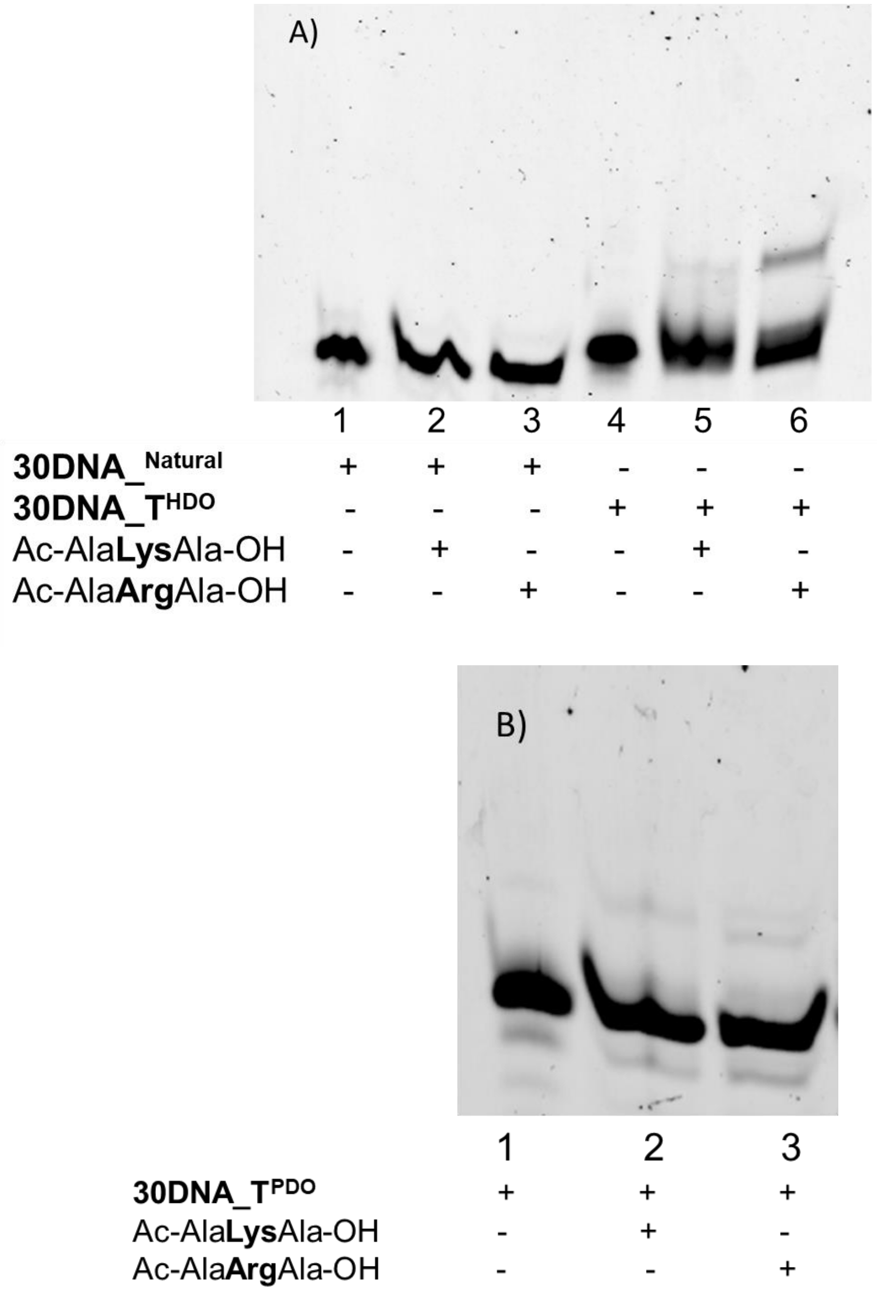

Figure S5.: Denaturing PAGE analysis comparing conjugations of A) **30DNA_**$^{\textbf{Natural}}$ (0,83 µM lanes 1,2,3) **30DNA_T**$^{\textbf{HDO}}$ (0.83 µM lanes 4,5,6) with tripeptide Ac-Ala**Lys**Ala-OH (250 mM, lanes 2,5) and with tripeptide Ac-Ala**Arg**Ala-OH (250 mM, lanes 3,6) in $NaHCO_3$ buffer (0.5 M, pH 10) at 37 °C for 18 h. B) **30DNA_T**$^{\textbf{PDO}}$ (0.83 µM lanes 1,2,3) with tripeptide Ac-Ala**Lys**Ala-OH (250 mM, lane 2) and with tripeptide Ac-Ala**Arg**Ala-OH (250 mM, lane 3) in $NaHCO_3$ buffer (0.5 M, pH 10) at 37 °C for 18 h.

2.4.3. Reaction with hexapeptide Ac-GAKKAA-NH_2 and Ac-KGAAKA-NH_2.

30DNA_T$^{\mathbf{Natural\ a}}$ or **30DNA_T**$^{\mathbf{PDOa}}$ were prepared by PEX in semi-preparative scale as described above (Section 2.2). The product was purified on QIAquick Nucleotide Removal Kit eluted with water and lyophilized. The reaction mixture (20 µL) contained **30DNA_T**$^{\mathbf{Natural\ a}}$ or **30DNA_T**$^{\mathbf{HDOa}}$ (0.83 µM) and Ac-GAKKAA-NH_2 or Ac-KGAAKA-NH_2 (250 mM) in $NaHCO_3$ buffer (0.5 M, pH 10) and was incubated for 18 h at 37 °C in a thermal cycler and subsequently quenched by addition of PAGE stop solution (20 µL) and heated 5 min at 95 °C. Samples were separated by denaturing 20 % PAGE (TBE 1X, 42 mA, 1.5 h) and visualized with florescence imaging. (Figure S6).

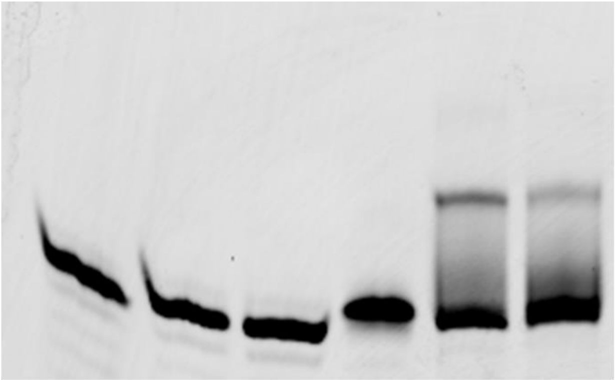

	1	2	3	4	5	6
**30DNA_**^{Natural}	+	+	+	-	-	-
**30DNA_T**^{HDO}	-	-	-	+	+	+
Ac-GlyAla**LysLys**AlaAla-NH_2	-	+	-	-	+	-
Ac-**Lys**GlyAlaAla**Lys**Ala-NH_2	-	-	+	-	-	+

Figure S6: Denaturing PAGE analysis comparing conjugations of **30DNA_**$^{\mathbf{Natural}}$ (0.83 µM lanes 1,2,3) or **30DNA_T**$^{\mathbf{HDO}}$ (0.83 µM lanes 4,5,6) with hexapeptide Ac-GlyAlaLysLysAlaAla-NH_2 (250 mM, lanes 2,5) or Ac-LysGlyAlaAlaLysAla-NH_2 (250 mM, lanes 3,6) in $NaHCO_3$ buffer (0.5 M, pH 10) at 37 °C for 18 h.

2.4.4. Reaction with decapeptides as Ac-L-SGYTAArgAESG-NH_2 and Ac-L-SGYTALysAESG-NH_2

30DNA_T$^{HDO\ a}$ and **30DNA_T^{PDOa}** was prepared by PEX in semi-preparative scale as described above (Section 2.2). The product was purified on QIAquick Nucleotide Removal Kit eluted with water and lyophilized. The reaction mixture (20 µL) contained **30DNA_T$^{HDO\ a}$** or **30DNA_T^{PDOa}** (0.83 µM) and Ac-L-SGYTA**R**AESG-NH_2 and Ac-L-SGYTA**K**AESG-NH_2 in $NaHCO_3$ buffer (0.5 M, pH 10) and was incubated for18 h at 37 °C in a thermal cycler and subsequently quenched by addition of PAGE stop solution (20 µL) and heated 5 min at 95 °C. Samples were separated by denaturing 20 % PAGE (TBE 1X, 42 mA, 1.5 h) and visualized with florescence imaging. (Figure S7).

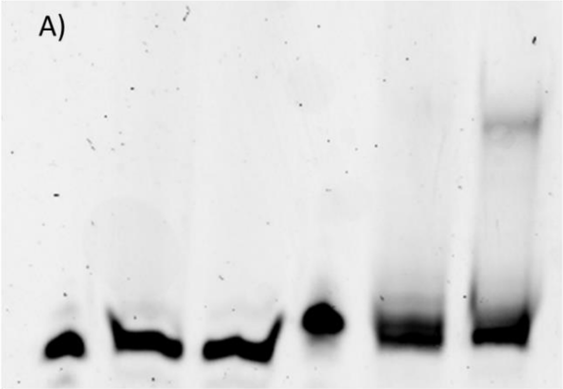

	1	2	3	4	5	6
**30DNA_^{Natural}**	+	+	+	-	-	-
**30DNA_T^{HDO}**	-	-	-	+	+	+
Ac-SGYTA**Lys**AESG-NH_2	-	+	-	-	+	-
Ac-SGYTA**Arg**AESG-NH_2	-	-	+	-	-	+

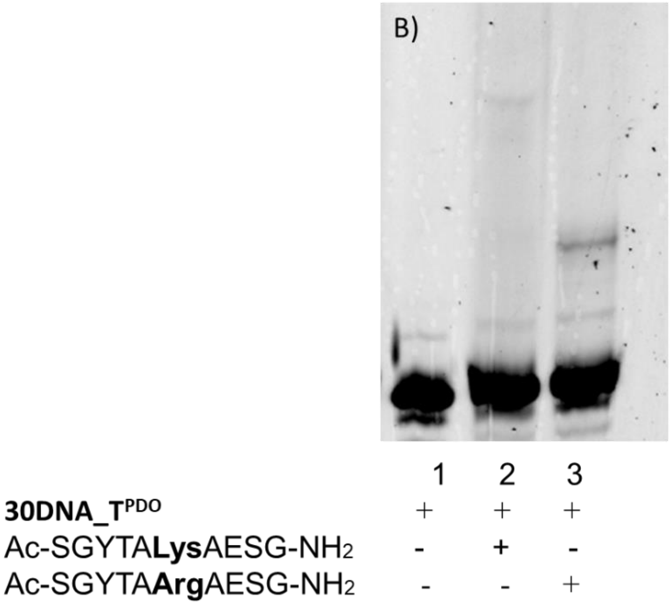

Figure S7 Denaturing PAGE analysis comparing conjugations of A) **30DNA_$^{\mathbf{Natural}}$** (0,83 µM, lanes 1,2,3) **30DNA_T$^{\mathbf{HDO}}$** (0.83 µM, lanes 4,5,6) with Ac-L-SGYTA**K**AESG-NH_2 (250 mM, lanes 2,5) or Ac-L-SGYTA**R**AESG-NH_2 (250 mM, lanes 3,6) (0.5 M, pH 10) in $NaHCO_3$ buffer at 37 °C for 18 h. B) **30DNA_T$^{\mathbf{PDO}}$** (0.83 µM, lanes 1,2,3) with Ac-L-SGYTA**K**AESG-NH_2 (250 mM, lane 2) and Ac-L-SGYTA**R**AESG-NH_2 (250 mM, lane 3) in $NaHCO_3$ buffer (0.5 M, pH 10) at 37 °C for 18 h.

2.4.5. Reaction with decapeptides as Ac-L-SGYTALysAQSG-NH_2 and further reductive amination- PAGE analysis.

30DNA_T$^{\mathbf{HDO\ a}}$ and **30DNA_T$^{\mathbf{PDO\ a}}$** were prepared by PEX in semi-preparative scale as described above (Section 2.2). The product was purified on QIAquick Nucleotide Removal Kit eluted with water and lyophilized. The reaction mixture (20 µL) contained **30DNA_T$^{\mathbf{HDO\ a}}$** or **30DNA_T$^{\mathbf{PDO\ a}}$** (0.83 µM) and Ac-L-SGYTA**K**AESG-NH_2 in $NaHCO_3$ buffer (0.5 M, pH 10) and was incubated for 1 h at 37°C in a thermal cycler and subsequently a solution containing $NaBH_3CN$ (1 M, 2.16 µL) was added and the reaction was incubated for 18 h at 37 °C. The reaction was quenched by addition of PAGE stop solution (20 µL) and heated 5 min at 95 °C. Samples were separated by denaturing 20 % PAGE (TBE 1X, 42 mA, 1.5 h) and visualized with florescence imaging. (Figure S8).

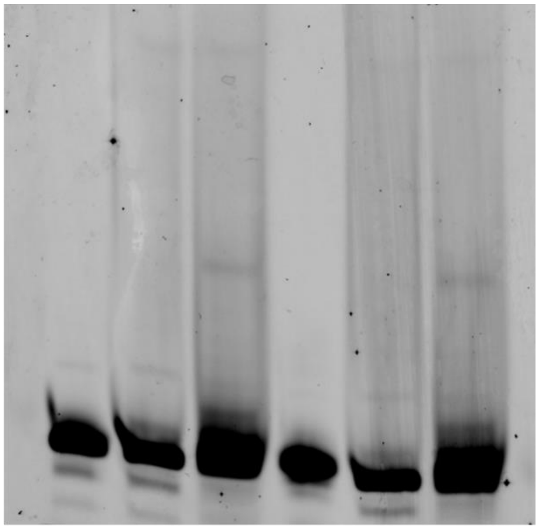

	1	2	3	4	5	6
**30DNA_T^{PDO}**	+	+	+	-	-	-
**30DNA_T^{HDO}**	-	-	-	+	+	+
Ac-SGYTA**Lys**AESG-NH_2	-	+	+	-	+	+
$NaBH_3CN$	-	-	+	-	-	+

Figure S8 Denaturing PAGE analysis comparing conjugations of **30DNA_T^{PDO}** (0,83 µM, lanes, 1,2,3) and **30DNA_T^{HDO}** (0.83 µM lanes, 4,5,6) with Ac-L-SGYTA**K**AESG-NH_2 (250 mM, lanes 2,5), samples treated with $NaBH_3CN$ for 1 h (1 M solution, lanes 3,6) and without (lanes 2,5) in $NaHCO_3$ buffer (0.5 M, pH 10) at 37 °C for 18 h.

2.4.6. Reaction with tripeptide NH_2-AlaGlyAla-OH

30DNA_T$^{Natural\ a}$ or **30DNA_T^{HDOa}** were prepared by PEX in semi-preparative scale as described above (Section 2.2). The product was purified on QIAquick Nucleotide Removal Kit eluted with water and lyophilized. The reaction mixture (20 µL) contained **30DNA_T$^{Natural\ a}$** or **30DNA_T^{HDOa}** (0.83 µM) and NH_2-AlaGlyAla-OH (250 mM) in $NaHCO_3$ buffer (0.5 M, pH 10) and was incubated for18 h at 37 °C in a thermal cycler. Subsequently was quenched by addition of PAGE stop solution (20 µL) and heated 5 min at 95 °C. Samples were separated by denaturing 20 % PAGE (TBE 1X, 42 mA, 1.5 h) and visualized with florescence imaging. (Figure S9).

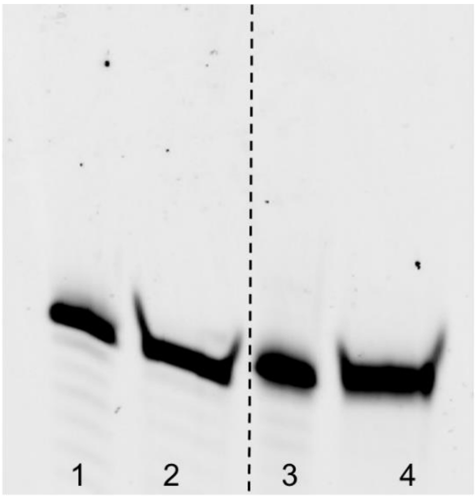

	1	2	3	4
**30DNA_^{Natural}**	+	+	-	-
**30DNA_T^{HDO}**	-	-	+	+
NH_2-AlaGlyAla-OH	-	+	-	+

Figure S9.: Denaturing PAGE analysis comparing conjugations of **30DNA_^{Natural}** (0.83 µM lanes 1,2) or **30DNA_T^{HDO}** (0.83 µM lanes 3,4) with tripeptide NH_2-AlaGlyAla-OH (250 mM, lanes 2,4) in $NaHCO_3$ buffer (0.5 M, pH 10) at 37 °C for 18 h.

2.4.7 Reaction with undecapeptide Ac-L-AHIDKLEMSTA-NH_2 and decapeptide Ac-L-FNEWCGPYVA-NH_2

30DNA_T$^{Natural\ a}$ and **30DNA_T^{HDOa}** was prepared by PEX in semi-preparative scale as described above (Section 2.2). The product was purified on QIAquick Nucleotide Removal Kit eluted with water and lyophilized. The reaction mixture (20 µL) contained **30DNA_T$^{Natural\ a}$** or **30DNA_T^{HDOa}** (0.83 µM) and Ac-L-AHIDKLEMSTA-NH_2 and Ac-L-FNEWCGPYVA-NH_2 in $NaHCO_3$ buffer (0.5 M, pH 10) and was incubated for18 h at 37 °C in a thermal cycler and subsequently quenched by addition of PAGE stop solution (20 µL) and heated 5 min at 95 °C. Samples were separated by denaturing 20 % PAGE (TBE 1X, 42 mA, 1.5 h) and visualized with florescence imaging. (Figure S10).

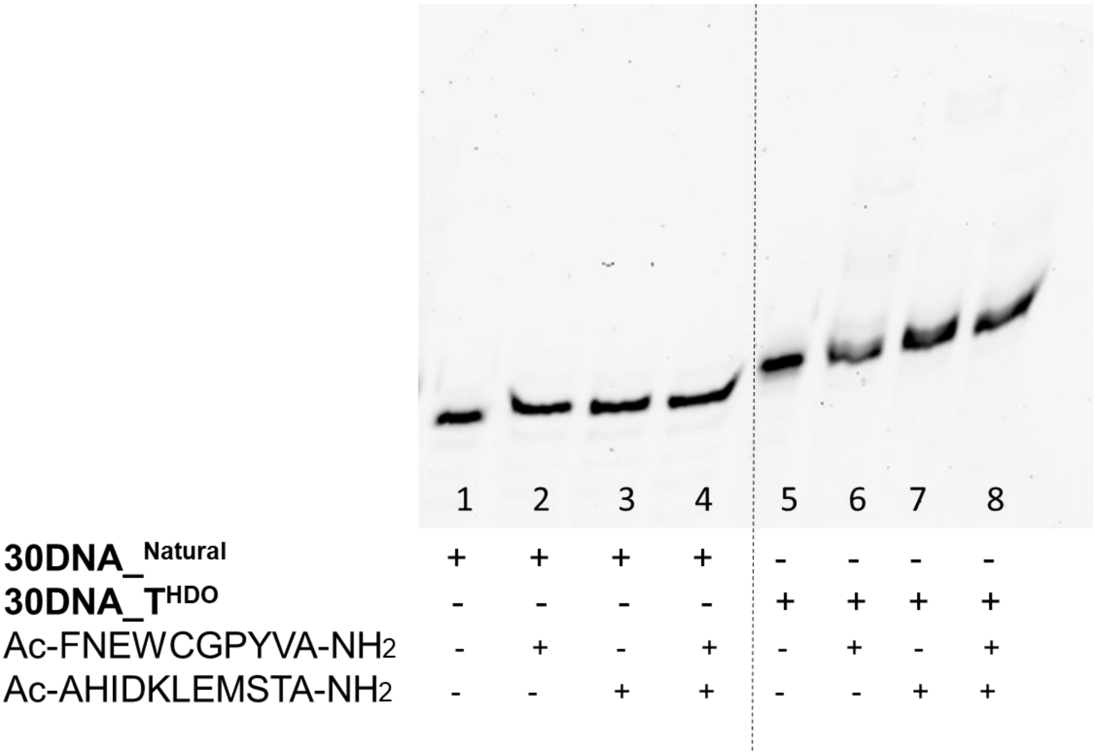

Figure S10 Denaturing PAGE analysis comparing conjugations of **30DNA_$^{\mathbf{Natural}}$** (0.83 µM, lanes 1,2,3,4) or **30DNA_T$^{\mathbf{HDO}}$** (0.83 µM, lanes 5,6,7,8) with Ac-L-FNEWCGPYVA-NH_2 (250 mM, lanes 2,4,6,8) and Ac-L-AHIDKLEMSTA-NH_2 (250 mM, lanes 3,4,7,8) in $NaHCO_3$ buffer (0.5 M, pH 10) at 37 °C for 18 h.

2.4.8. Table S5. List of amino acids and synthetic peptides used in cross-linking reactions with 19/30DNA_T^{HDO} and 30DNA_T^{PDO} and conversions of cross-linking reactions.

	Peptide sequence	Mw [Da]	Conversion [%] **19DNA_T^{HDO}**	Conversion [%] **30DNA_T^{HDO}**	Conversion [%] **30DNA_T^{PDO}**
	N-Boc-**Arg**-OH	274.32	10	-	-
Tripeptides	Ac-A**R**A-OH	358.2	10	13	-
	Ac-A**K**A-OH	330.2	-	0.001	-
	NH_2-AGA-OH	217.1	-	0.03	-
Decapeptides	Ac-SGYTA**R**AESG-NH_2	1038.5	35	16	-
	Ac-SGYTA**K**AESG-NH_2	1011.4	-	0.03	-
	Ac-AHIDKLEMSTA-NH_2	1255.6	-	0.03	-
Undecapeptide	Ac-FNEWCGPYVA-NH_2	1224.5	-	0.03	-
Heptapeptides	Ac-(**R**)$_7$-NH_2	1152.7	-	45	1.3
	Ac-(**K**)$_7$-NH_2	956.7	-	9	1
Hexapeptides	Ac-GA**KK**AA-NH_2	586.3	-	11	-
	Ac-**K**GAA**K**A-NH_2	586.3	-	9	-

2.5. Cross-linking of 19/30DNA_T^{HDO} and individual recombinant proteins (BSA, H2A, H2B, H3.1 and H4)-(SDS analysis)

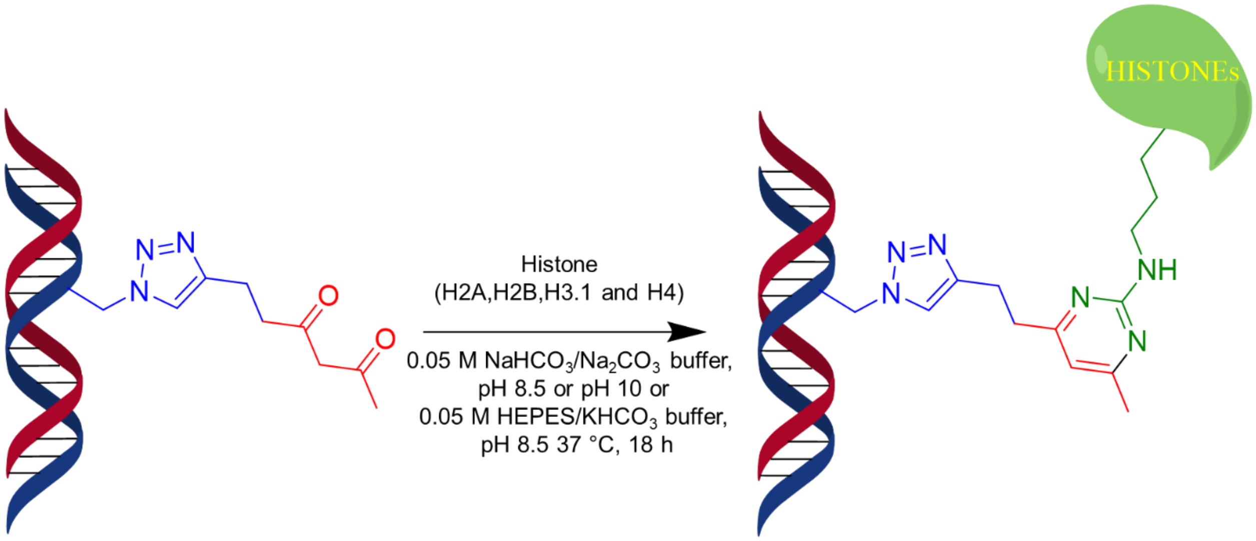

2.5.1. Scheme S5. Cross-linking of modified **19/30DNA_T^{HDO}** with histone recombinant proteins.

2.5.2. Reaction of 30DNA_T^{HDO} with individual recombinant proteins

30DNA_T^{HDO} [a] and **30DNA_^{Natural}** were prepared by PEX in semi-preparative scale as described above (Section 2.2). **30DNA_^{Natural}** or **30DNA_T^{HDO}** (2.5 µM) was incubated with individual proteins (10 µM) either in 10 µL of $NaHCO_3$ buffer (0.05 M, pH 10), $NaHCO_3$ buffer (0.05 M, pH 8.5) or HEPES/$KHCO_3$ buffer (0.05 M, pH 8.5) at 37° C for 18 h. The reaction was diluted in 2X VPS loading buffer, denatured for 2 min at 100 °C and analysed by 17.5 % SDS denaturing PAGE at room temperature (200 V, 50 min). Visualization was performed by fluorescence imaging (Figure S11 A, Figure S12 A,B,C) and subsequently stained with PageBlue ™ protein staining solution (Figure S11 B).

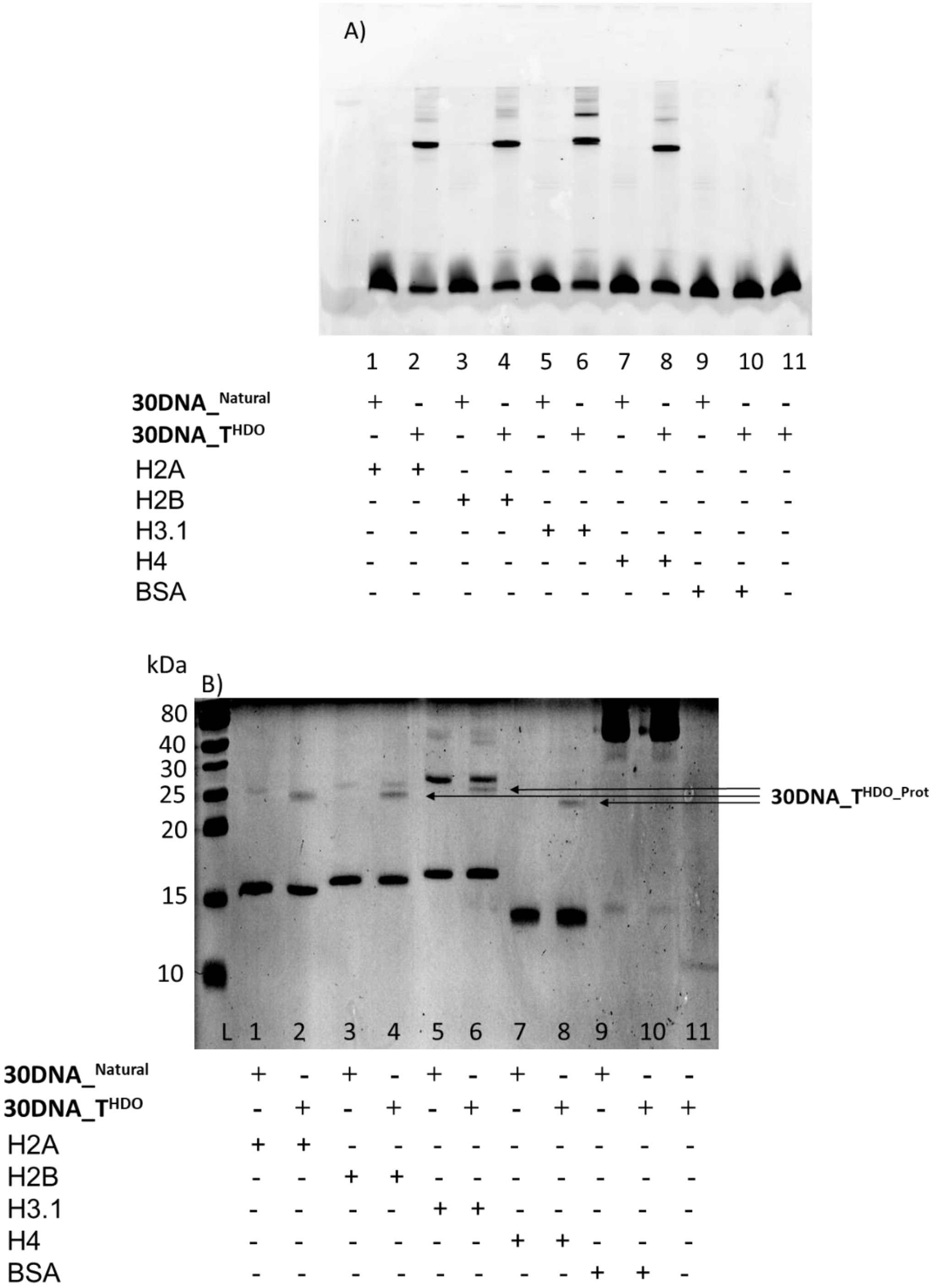

Figure S11. A) 17.5 % SDS-PAGE analysis of DNA-protein cross-linking of **30DNA_^{Natural}** (lanes 1,3,5,7,9,) or **30DNA_T^{HDO}** (lanes 2,4,6,8,10,11) with recombinant protein such as H2A (lanes 1,2), H2B (lanes 3,4), H3.1 (lanes 5,6), H4 (lanes 7,8) and BSA (lanes 9,10) in $NaHCO_3$ buffer (0.05 M, pH 10) at 37 °C for 18 h. B) Post-staining using PageBlue ™ protein staining solution[5].

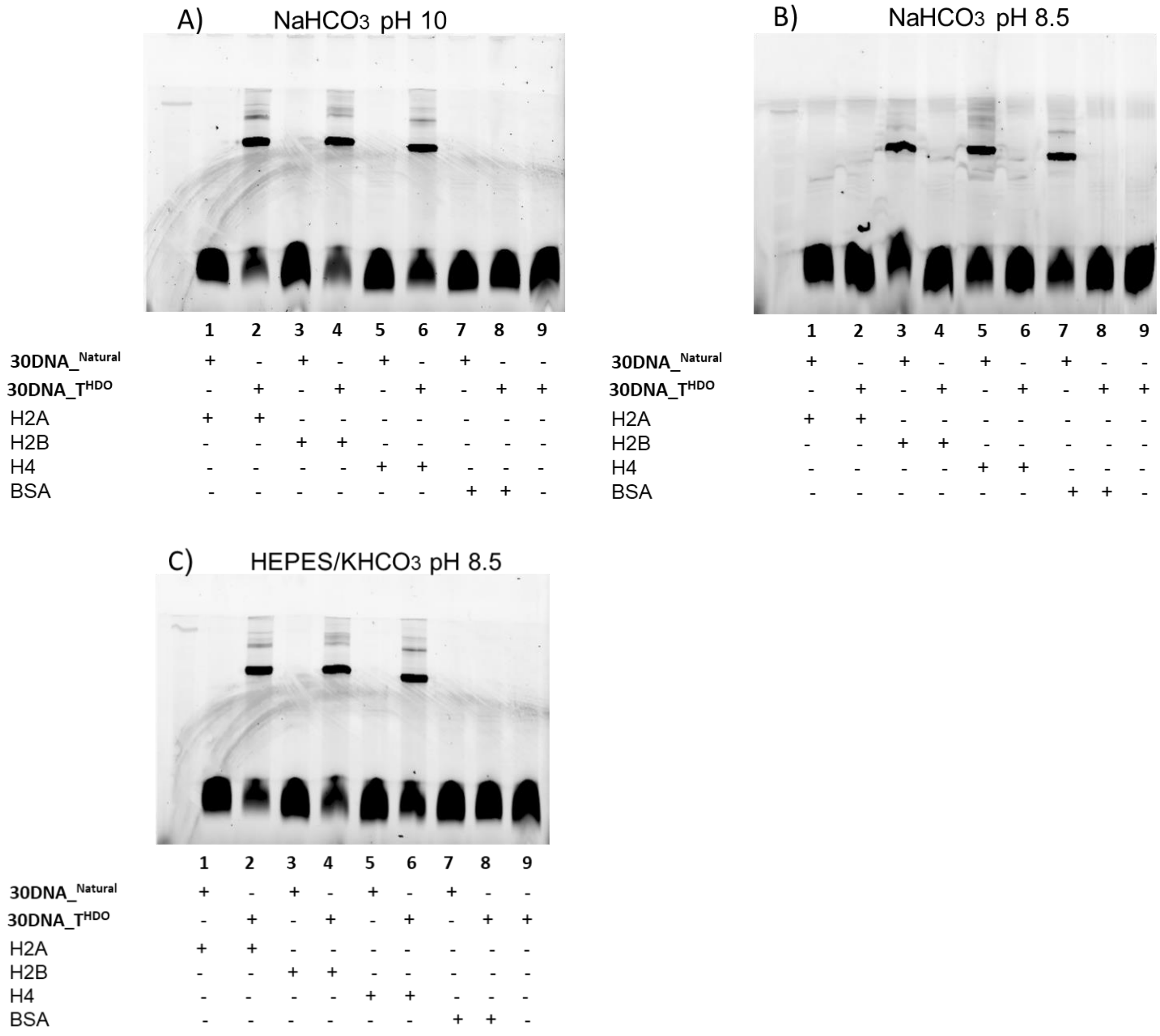

Figure S12. 17.5 % SDS-PAGE analysis of DNA-protein cross-linking of **30DNA_$^{\textbf{Natural}}$** (lane 1,3,5,7) or **30DNA_T$^{\textbf{HDO}}$** (lanes 2,4,6,8,9) with recombinant protein such as H2A (lanes 1,2), H2B (lanes 3,4), H4 (lanes 5,6) and BSA (lanes 7,8) in A) $NaHCO_3$ buffer (0.05 M, pH 10) at 37 °C for 18 h. B) $NaHCO_3$ buffer (0.05 M, pH 8.5) at 37 °C for 18 h. C) HEPES/$KHCO_3$ buffer (0.05 M, pH 8.5) at 37 °C for 18 h.

2.5.3. Reaction of 30DNA_T^{HDO} with individual recombinant proteins with further treatment with NH_2OH

30DNA_T^{HDO} ^{a} and **30DNA_^{Natural}** were prepared by PEX in semi-preparative scale as described above (Section 2.2). **30DNA_^{Natural}** or **30DNA_T^{HDO}** (2.5 µM) was incubated with individual proteins (10 µM) in 10 µL of $NaHCO_3$ buffer (0.05 M, pH 10) at 37° C for 18 h.
After the reaction was treated with 2M NH_2OH (1 µL) at 37° C for 15 minutes [6]. The reaction was diluted in 2X VPS loading buffer, denatured for 2 min at 100 °C and analysed by 17.5 % SDS denaturing PAGE at room temperature (200 V, 50 min). Visualization was performed by fluorescence imaging (Figure S13).

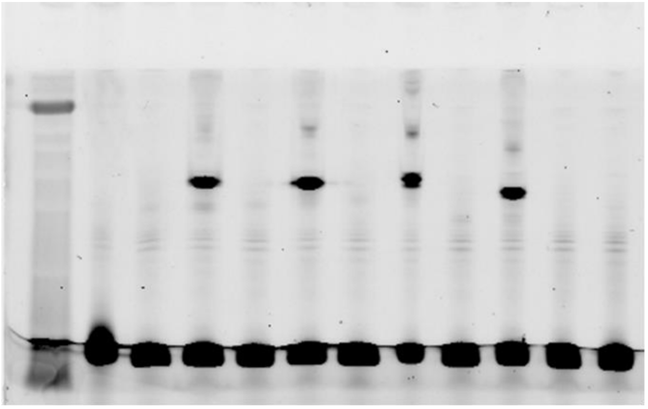

	1	2	3	4	5	6	7	8	9	10	11
**DNA_^{Natural}**	-	+	-	+	-	+	-	+	-	+	-
**30DNA_T^{HDO}**	+	-	+	-	+	-	+	-	+	-	+
H2A	-	+	+	-	-	-	-	-	-	-	-
H2B	-	-	-	+	+	-	-	-	-	-	-
H3.1	-	-	-	-	-	+	+	-	-	-	-
H4	-	-	-	-	-	-	-	+	+	-	-
BSA	-	-	-	-	-	-	-	-	-	+	+
2M NH_2OH	-	+	+	+	+	+	+	+	+	+	+

Figure S13. 17.5 % SDS-PAGE analysis of DNA-protein cross-linking of **30DNA_^{Natural}** (lanes 2,4,6,8,10) or **30DNA_T^{HDO}** (lanes 1,3,5,7,9,11) with recombinant protein such as H2A (lanes 2,3), H2B (lanes 4,5), H3.1 (lanes 6,7), H4 (lanes 8,9) and BSA (lanes 10,11) in $NaHCO_3$ buffer (0.05 M, pH 10) at 37 °C for 18 h and subsequently with 2 M NH_2OH at 37 °C for 15 min.

2.5.4. Reaction of 30DNA_T^{HDO} with individual recombinant proteins (ratio 1:1)

30DNA_T$^{HDO\ a}$ and **30DNA_^{Natural}** were prepared by PEX in semi-preparative scale as described above (Section 2.2). **30DNA_^{Natural}** or **30DNA_T^{HDO}** (2.5 μM) was incubated with individual proteins (2.5 μM) in 10 μL of $NaHCO_3$ buffer (0.05 M, pH 10) at 37° C for 18 h. The reaction was diluted in 2X VPS loading buffer, denatured for 2 min at 100 °C and analysed by 17.5 % SDS denaturing PAGE at room temperature (200 V, 50 min). Visualization was performed by fluorescence imaging (Figure S14).

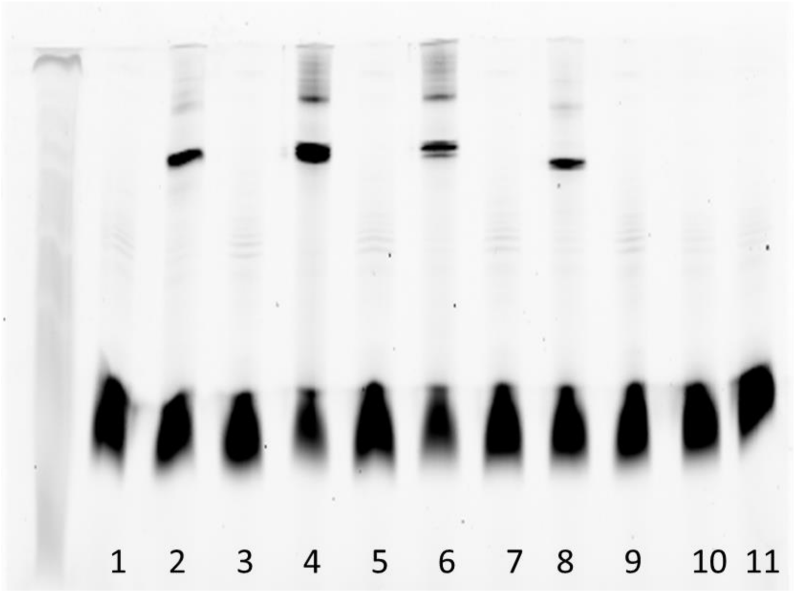

	1	2	3	4	5	6	7	8	9	10	11
30DNA_^{Natural}	+	-	+	-	+	-	+	-	+	-	-
30DNA_T^{HDO}	-	+	-	+	-	+	-	+	-	+	+
H2A	+	+	-	-	-	-	-	-	-	-	-
H2B	-	-	+	+	-	-	-	-	-	-	-
H3.1	-	-	-	-	+	+	-	-	-	-	-
H4	-	-	-	-	-	-	+	+	-	-	-
BSA	-	-	-	-	-	-	-	-	+	+	-

Figure S14. 17.5% SDS-PAGE analysis of DNA-protein cross-linking of **30DNA_^{Natural}** (lanes 1,3,5,7,9,) or **30DNA_T$^{HDO\ a}$** (lanes 2,4,6,8,10,11) with recombinant protein such as H2A (lanes 1,2), H2B (lanes 3,4), H3.1 (lanes 5,6) , H4 (lanes7,8) and BSA (9,10) in , $NaHCO_3$ buffer (0.05 M, pH 10) at 37 °C for 18 h.

2.5.5. Reaction of 19DNA_T^{HDO} with individual recombinant proteins

19DNA_T^{HDOa} and **19DNA_^{Natural}** were prepared by PEX in semi-preparative scale as described above. **19DNA_^{Natural}** or **19DNA_T^{HDOa}** (2.5 μM) was incubated with individual proteins (10 μM) either in 10 μL of $NaHCO_3$ buffer (0.05 M, pH 10) at 37° C for 18 h. The reaction was diluted in 2X VPS loading buffer, denatured for 2 min at 100 °C and analysed by 17.5 % SDS denaturing PAGE at room temperature (200 V, 50 min). Visualization was performed by fluorescence imaging (Figure S15 A) and subsequently stained with PageBlue ™ protein staining solution (Figure S15 B) [5].

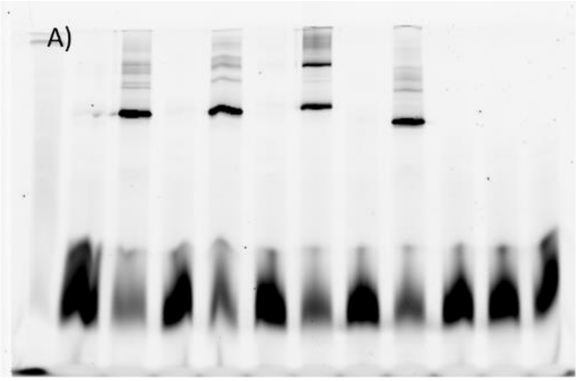

	1	2	3	4	5	6	7	8	9	10	11
**19DNA_^{Natural}**	+	-	+	-	+	-	+	-	+	-	-
**19DNA_T^{HDO}**	-	+	-	+	-	+	-	+	-	+	+
H2A	+	+	-	-	-	-	-	-	-	-	-
H2B	-	-	+	+	-	-	-	-	-	-	-
H3.1	-	-	-	-	+	+	-	-	-	-	-
H4	-	-	-	-	-	-	+	+	-	-	-
BSA	-	-	-	-	-	-	-	-	+	+	-

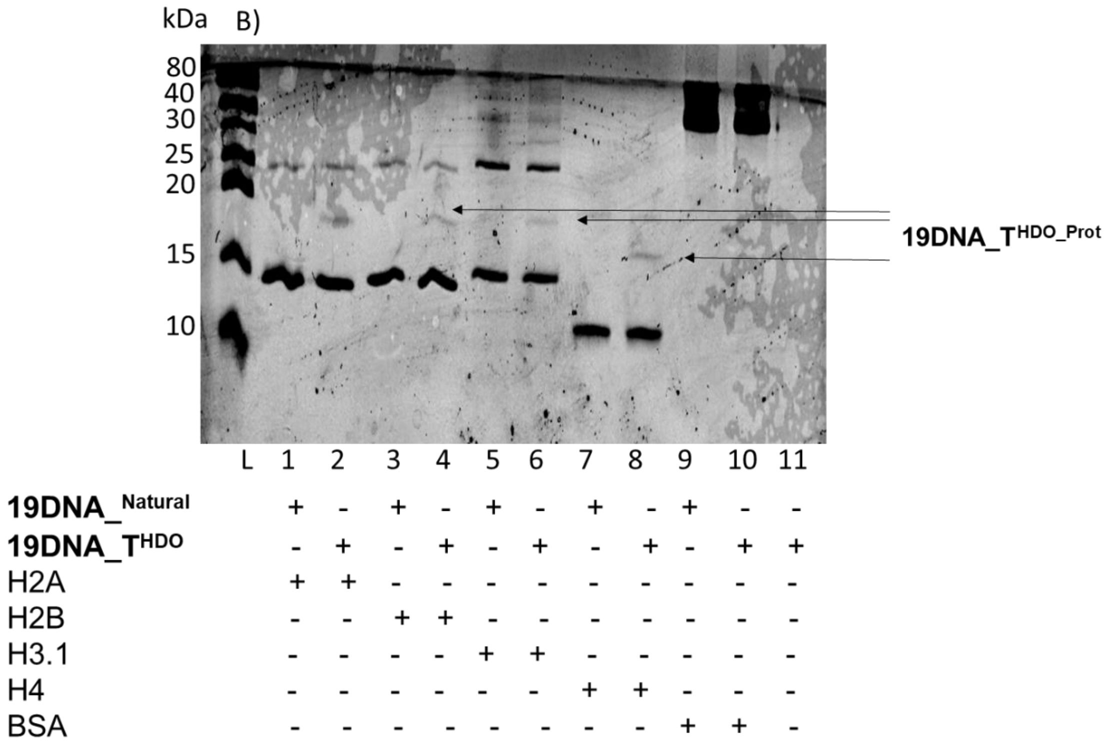

Figure S15. A) 17.5 % SDS-PAGE analysis of DNA-protein cross-linking of **19DNA_$^{\mathbf{Natural}}$** (lanes 1,3,5,7,9,) or **19DNA_T$^{\mathbf{HDO}}$** (lanes 2,4,6,8,10,11) with recombinant protein such as H2A (lanes 1,2), H2B (lanes 3,4), H3.1 (lanes 5,6) , H4 (lanes 7,8) and BSA (lanes 9,10) in $NaHCO_3$ buffer (0.05 M, pH 10) at 37 °C for 18 h. B) Post-staining using PageBlue ™ protein staining solution[5].

2.5.6 Comparison of cross-linking of 30DNA_T$^{\mathbf{HDO}}$ and 30DNA_T$^{\mathbf{PDO}}$ with H4 protein.

30DNA_T$^{\mathbf{HDO\ a}}$ and **30DNA_T$^{\mathbf{PDO\ a}}$** were prepared by PEX in semi-preparative scale as described above (Section 2.2). **30DNA_$^{\mathbf{Natural\ a}}$**, **30DNA_T$^{\mathbf{HDO\ a}}$** and **30DNA_T$^{\mathbf{PDO\ a}}$**(2.5 µM) was incubated with H4 protein (10 µ M) in 10 µL of either $NaHCO_3$ buffer (0.05 M, pH 10) or HEPES/$KHCO_3$ buffer (0.05 M, pH 8.5) at 37 °C for 18 h. The reaction was diluted in 2X VPS loading buffer, denatured for 2 min at 100 °C and analysed by 17.5 % SDS denaturing PAGE. Visualization was performed by fluorescence imaging (Figure S16 A) and subsequently stained by PageBlue ™ protein staining solution (Figure S16 B).

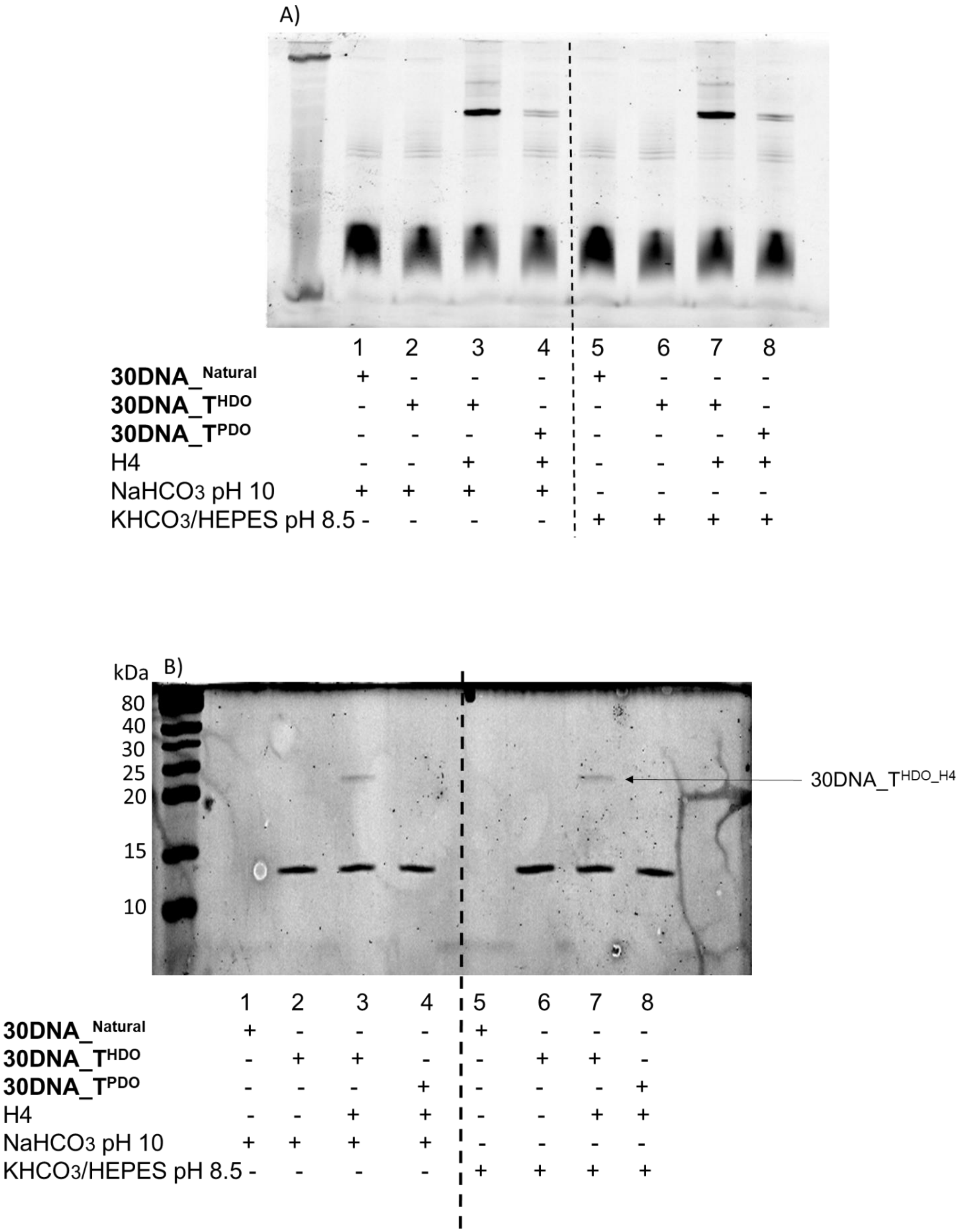
A)
1 2 3 4 5 6 7 8
30DNA_Natural + - - - + - - -
30DNA_THDO - + + - - + + -
30DNA_TPDO - - - + - - - +
H4 - - + + - - + +
NaHCO3 pH 10 + + + + - - - -
KHCO3/HEPES pH 8.5 - - - - + + + +
kDa
B)
80
40
30
25
20
15
10
30DNA_THDO_H4
1 2 3 4 5 6 7 8
30DNA_Natural + - - - + - - -
30DNA_THDO - + + - - + + -
30DNA_TPDO - - - + - - - +
H4 - - + + - - + +
NaHCO3 pH 10 + + + + - - - -
KHCO3/HEPES pH 8.5 - - - - + + + +

Figure S16. A) 17.5 % SDS-PAGE analysis of DNA-protein cross-linking of **30DNA_**[**Natural a**] (lanes 1,5) or **30DNA_T**[**HDO a**] (lanes 2,3,6,7) and **30DNA_T**[**PDO a**] (lanes 4,8) with recombinant protein H4 (lanes 2,3,4,6,7,8), in either $NaHCO_3$ buffer (0.05 M, pH 10) at 37 °C for 18 h (lanes 1,2,3,4) or $KHCO_3$/ HEPES buffer (0.05 M, pH 8.5) (Lanes 5,6,7,8) at 37 °C for 18 h. B) Post-staining using PageBlue ™ protein staining solution.

2.6. Kinetic studies of cross-linking of 30DNA_T^{HDO} with H4 protein.

30DNA_T[**HDOa**] was prepared by PEX in semi-preparative scale as described above. **30DNA_T**[**HDOa**] (2.5 µM) was incubated with H4 protein (10 µM) in 10 µL of $NaHCO_3$ buffer (0.05 M, pH 10) at 37 °C. Aliquots were taken from the reaction mixture at different times (30 min, 1 h, 7 h, 23 h, 30 h, 48 h 51 h). The samples were diluted in 2X VPS loading buffer, denatured for 2 min at 100 °C and analysed by 17.5 % SDS denaturing PAGE at room temperature (200 V, 50 min). Visualization was performed by fluorescence imaging (Figure S17 A) and subsequently staining with PageBlue ™ protein staining solution (Figure S17 B).

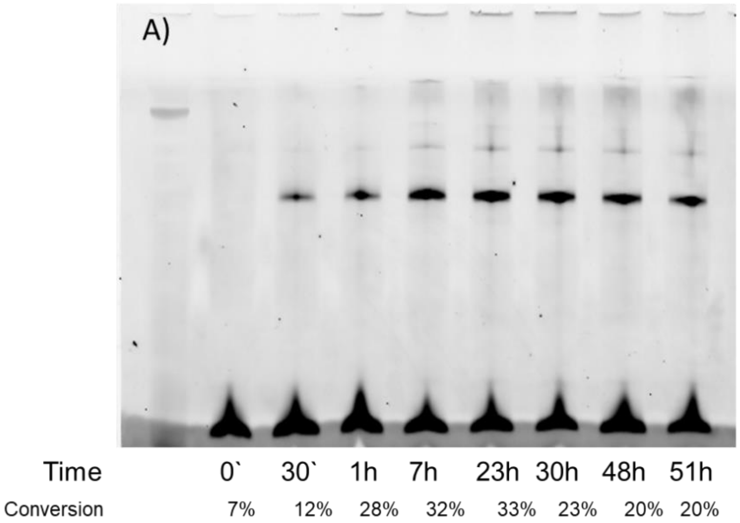

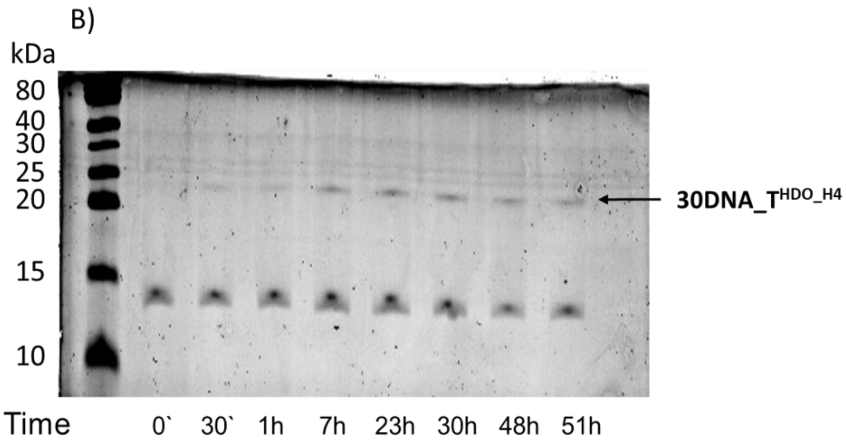

Figure S17. A) 17.5 % SDS PAGE analysis of reaction times about the formation of cross-linking between **30DNA_T^{HDO}** and histone H4. B) Post staining using PageBlue ^{TM} gel. Aliquots were taken from the reaction mixture at different times (30 min - 51h).

2.7. Stability studies of 30DNA_T^{HDO} in different buffers-PAGE analysis.

30DNA_T$^{HDO\ a}$ and **30DNA_T$^{PDO\ a}$** were prepared by PEX in semi-preparative scale as described above. **30DNA_T$^{HDO\ a}$** or **30DNA_T$^{PDO\ a}$** (2.5 μM) was incubated in 10 μL of either $NaHCO_3$ buffer (0.05 M, pH 10) or $KHCO_3$/HEPES (0.05 M, pH 8.5) at 37 °C in a thermal cycler for 18 h and for 168 h (1 week). Subsequently the reactions were quenched by addition of PAGE stop solution (20 μL) and heated 5 min at 95 °C. Samples were separated by denaturing 20 % PAGE (TBE1X, 42 mA, 1.5 h) and visualized with florescence imaging. (Figure S18).

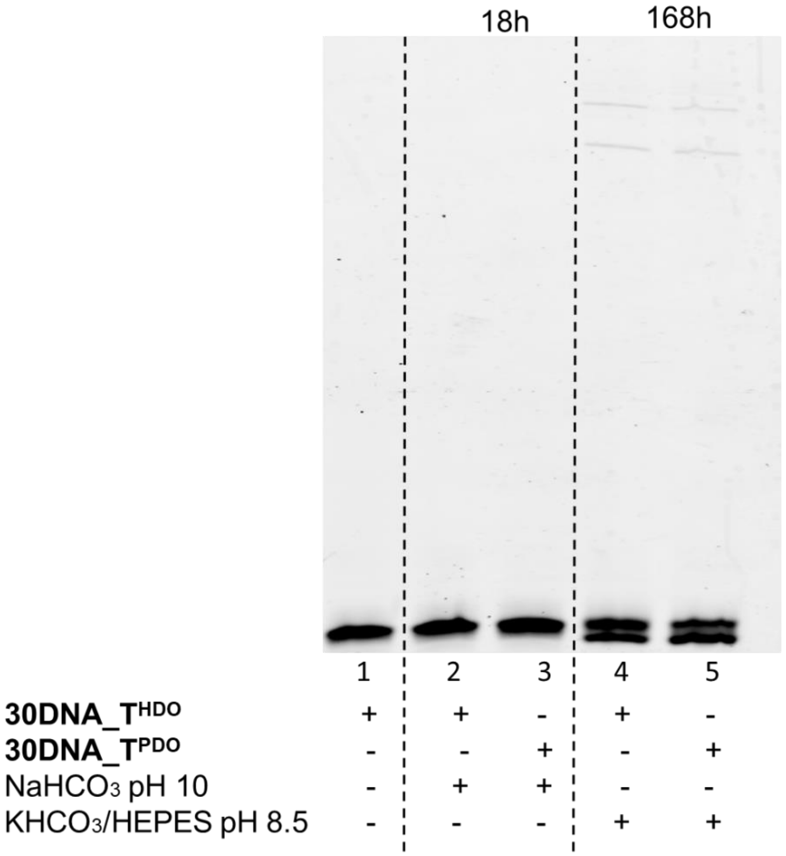

Figure S18. Denaturing PAGE analysis of stabilities studies of **30DNA_T$^{HDO\ a}$** (lanes 1,2,4) and **30DNA_T$^{PDO\ a}$** (lanes 3,5) in either $NaHCO_3$ buffer (0.05 M, pH 10), (lanes 2,3) or $KHCO_3$/HEPES (0.05 M, pH 8.5) (lanes 4,5) at 37 °C. Reactions kept for 18 h (lanes 2,3) and for 168 h (1 week), (lanes 4,5).

2.8. Cross-linking of 25DNA_T^{HDO} and GSTp53CD

25DNA_T$^{HDO\ a}$ and **25DNA_$^{Natural\ a}$** were prepared by PEX in semi-preparative scale with the same procedure described above for **30DNA_T^{HDO}**. The reaction mixtures (20 μL) contained **25DNA_T$^{HDO\ a}$** (40 ng/μL, 3 μL), KCl (500 mM, 4.8 μL), TCEP (2 mM, 4.8 μL), VP buffer (50 mM Tris, 0.1 % Triton-X100, pH 9; 4.8 μL) and GSTp53CD stock solution (400 ng/μL in 25 mM HEPES pH 7.6, 200 mM KCl, 10 % glycerol, 1 mM DTT, 1 mM benzamidine; 2.6 μL). Control samples were containing **25DNA_$^{Natural\ a}$** were prepared as described above without GSTp53CD. Samples were incubated for 18 h at 37 °C. 2X VPS loading buffer was added and the mixture was denatured 10 min at 65 °C and analysed by SDS-PAGE 10 % at room temperature (120 V/60 min). Visualization was performed by fluorescence imaging (Figure S19).

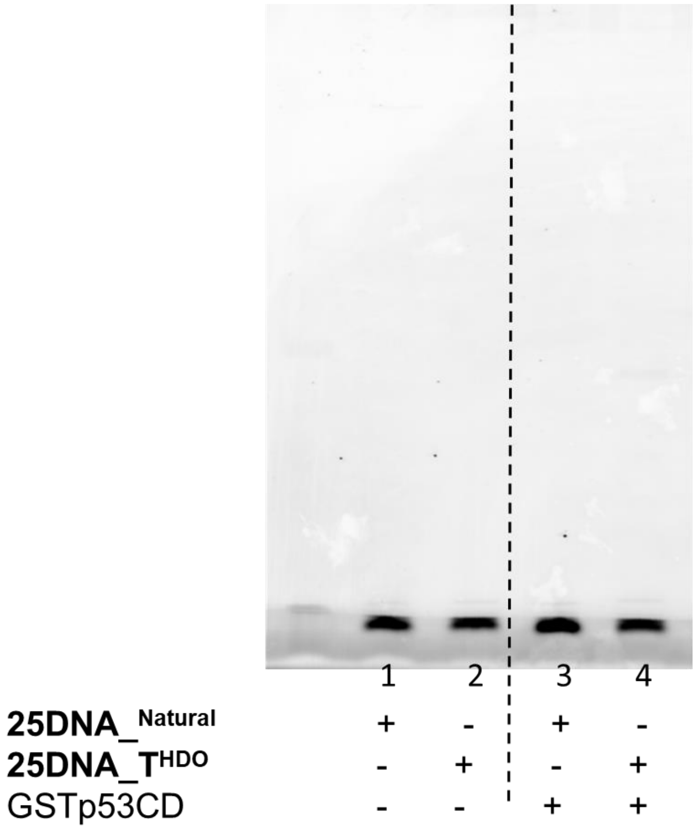

Figure S19. 10 % SDS-PAGE analysis of DNA-protein cross-link between **25DNA_^{Natural}** (lane 3) or **25DNA_T^{HDO}** (lane 4) and p53GSTCD (2 equivalents of protein to DNA), control **25DNA_$^{Natura\ l}$** (lane 1) and control **25DNA_T^{HDO}** (lane 2) are without addition of GSTp53CD.

2.9. Characterization of cross-links between 30DNA_T^{HDO} and individual recombinant proteins (H2A, H2B, H3.1, H4) by mass spectrometry

The Histone-conjugates of **30DNA_T^{HDO} [a]** and recombinant proteins (H2A, H2B, H3.1 and H4) were prepared as described above (Section 2.2). 5 μL of the reaction mixture was injected desalting column (MassPREP desalting, Waters) and desalted by fast gradient (4 min) of acetonitrile in water with 0.1% Formic acid. The separation was carried out by LC system (I-class, Waters) coupled to mass Spectrometer (Synapt G2, Waters) to acquire m/z by positive electrospray ionization. Raw mass spectra of chromatographic peak containing conjugate was combined, subtracted and deconvoluted by MaxEnt algorithm [7] (see Table S7 for spectrum see SI part 4).

2.9.1. Table S6. Overview of DNA-protein cross-linking reactions and their conversions.

Protein	Mw / [kDa]	DNA binding	N° Arg	**19/30DNA_T^{HDO}** (1:5) Conversion [%]	**30DNA_T^{HDO}** (1:1) Conversion [%]	**30DNA_T^{HDO} NH_2OH 2M** Conversion [%]
BSA	69.3	no	26	0	0	0
GST_p53 CD	78.5	yes	28	0	-	-
H2A	13.99	yes	11	32 / 35	16	24
H2B	13.79	yes	6	32 / 24	27	29
H3.1	15.27	yes	18	34 / 35	17	27
H4	11.24	yes	14	31 / 34	12	23

2.9.2. Table S7. Overview of DNA protein conjugates and MS analysis results.

	Mw(calc.) / [Da]	Mw(found) / [Da]
**30ON_T^{HDO_H2A}**	23297.3	23313.72
**30ON_T^{HDO_H2B}**	23097.0	23112.48
**30ON_T^{HDO_H4}**	20547.0	20560.51

3. Copies of mass spectra

3.1. MALDI-TOF spectra

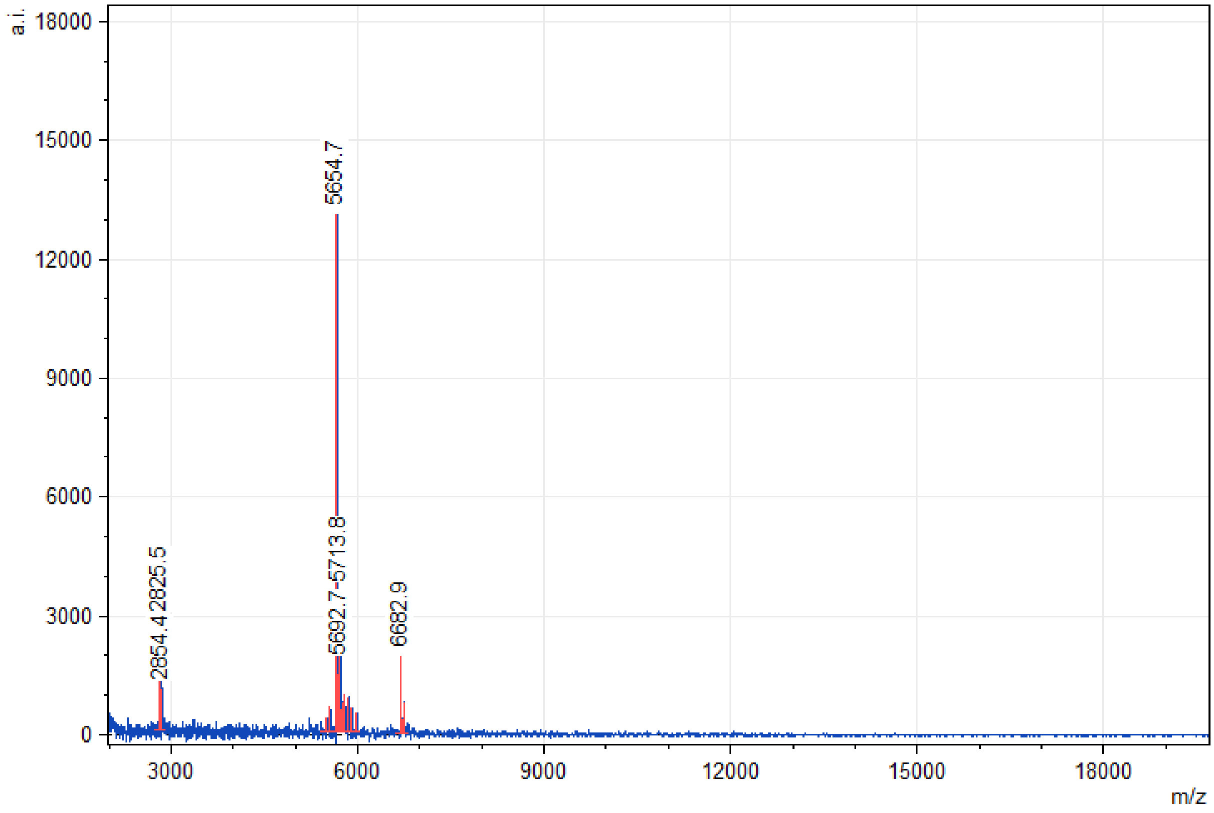

Figure S20. MALDI-TOF MS spectrum of **19DNA_T$^{HDO\ a}$**; Mass calculated for **19ON_T$^{HDO\ a}$** [M] 6681.98 Da; found 6682.9 Da; the peak at m/z=5654.7 Da is assigned to the template **Temp^{19_1T}**.

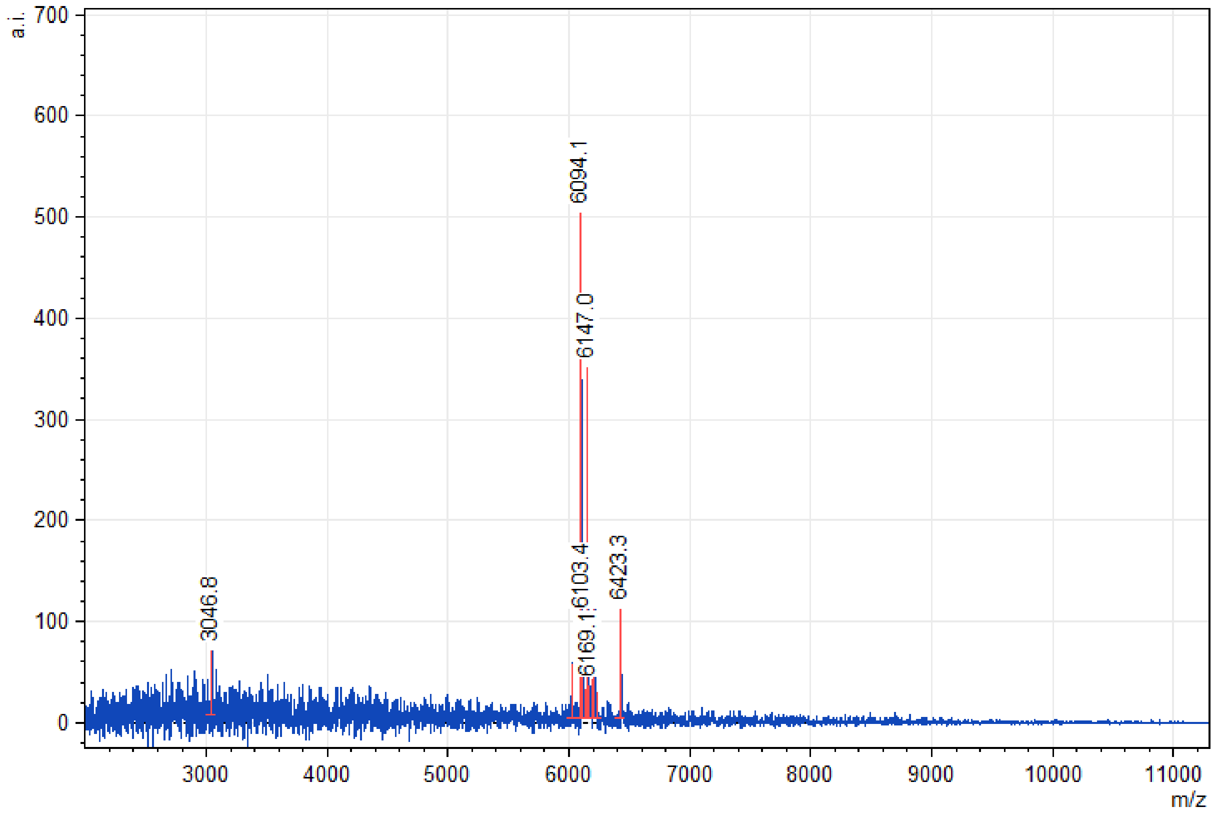

Figure S21. MALDI-TOF MS spectrum of **19DNA_T$^{HDO\ b}$**; Mass calculated for **19ON_T^{HDO}** [M] is 6145.98 Da; Mass found is 6147.0 Da; the peak at m/z=6094 Da is assigned to the template **Temp$^{19_1T\ b}$**.

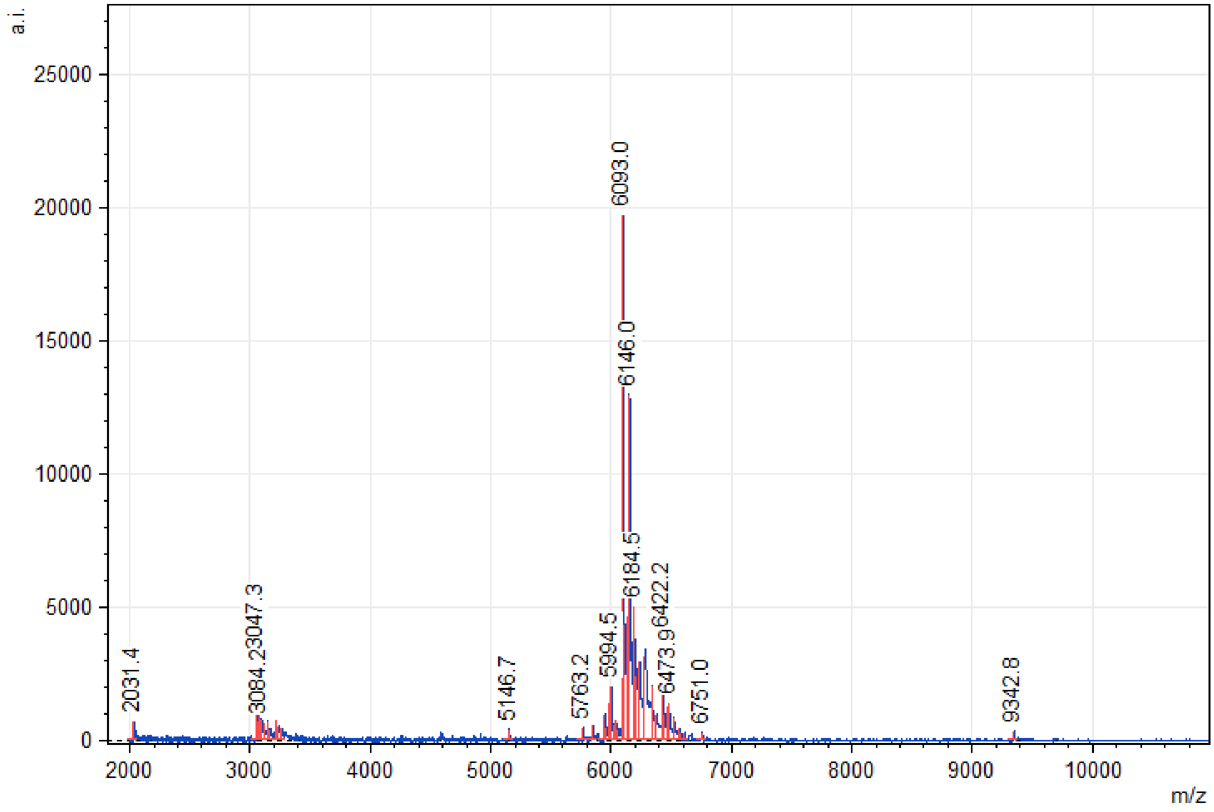

Figure S22. MALDI-TOF MS spectrum of **19DNA_T**$^{\mathbf{PDO}\ b}$; Mass calculated for **19DNA_T**$^{\mathbf{PDO}}$ [M] 6145.98 Da; Mass found 6146.0 Da; the peak at m/z=6093.0 Da is assigned to the template **Temp**$^{\mathbf{19\_1T}\ b}$.

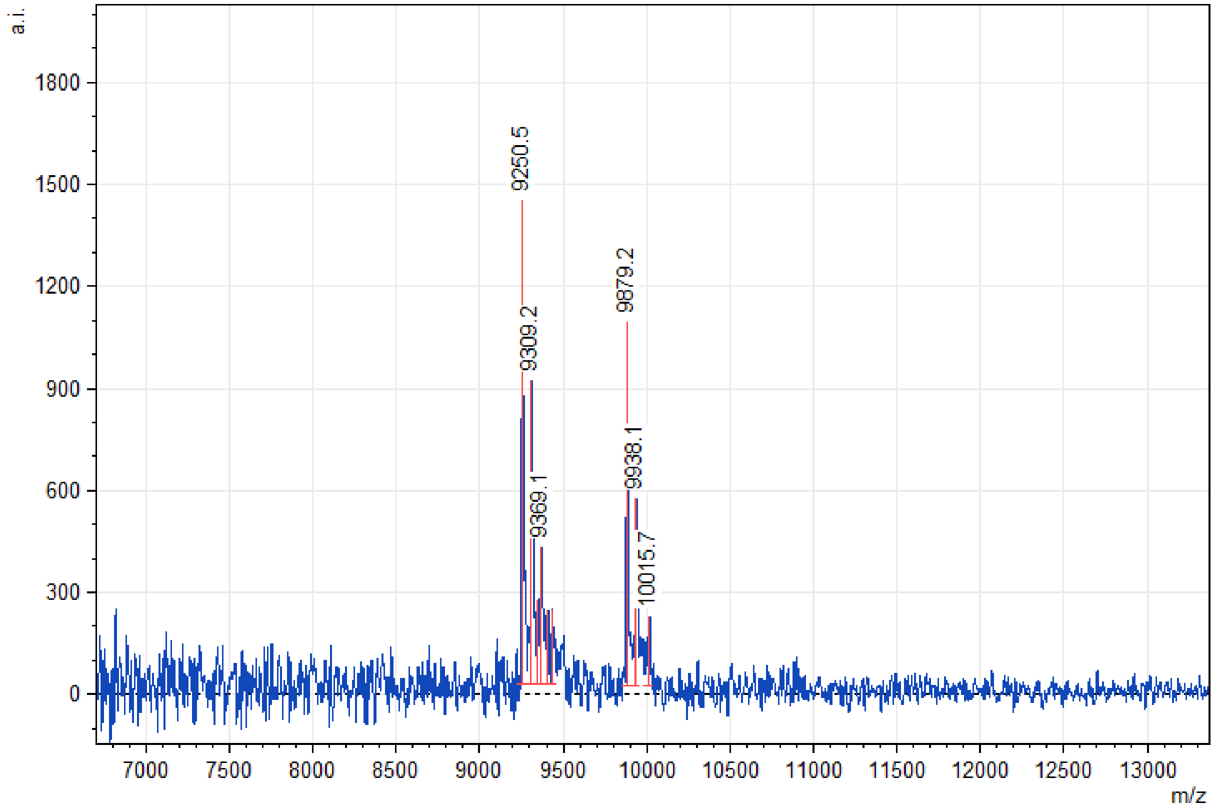

Figure S23. MALDI-TOF MS spectrum of **30DNA_T$^{HDO\ a}$**; Mass calculated for **30ON_T$^{HDO\ a}$** [M]: 9878.08 Da; Mass found 9879.2 Da; ; the peak at m/z=9250.5 Da is assigned to the template **Temp^{19_1T}** .

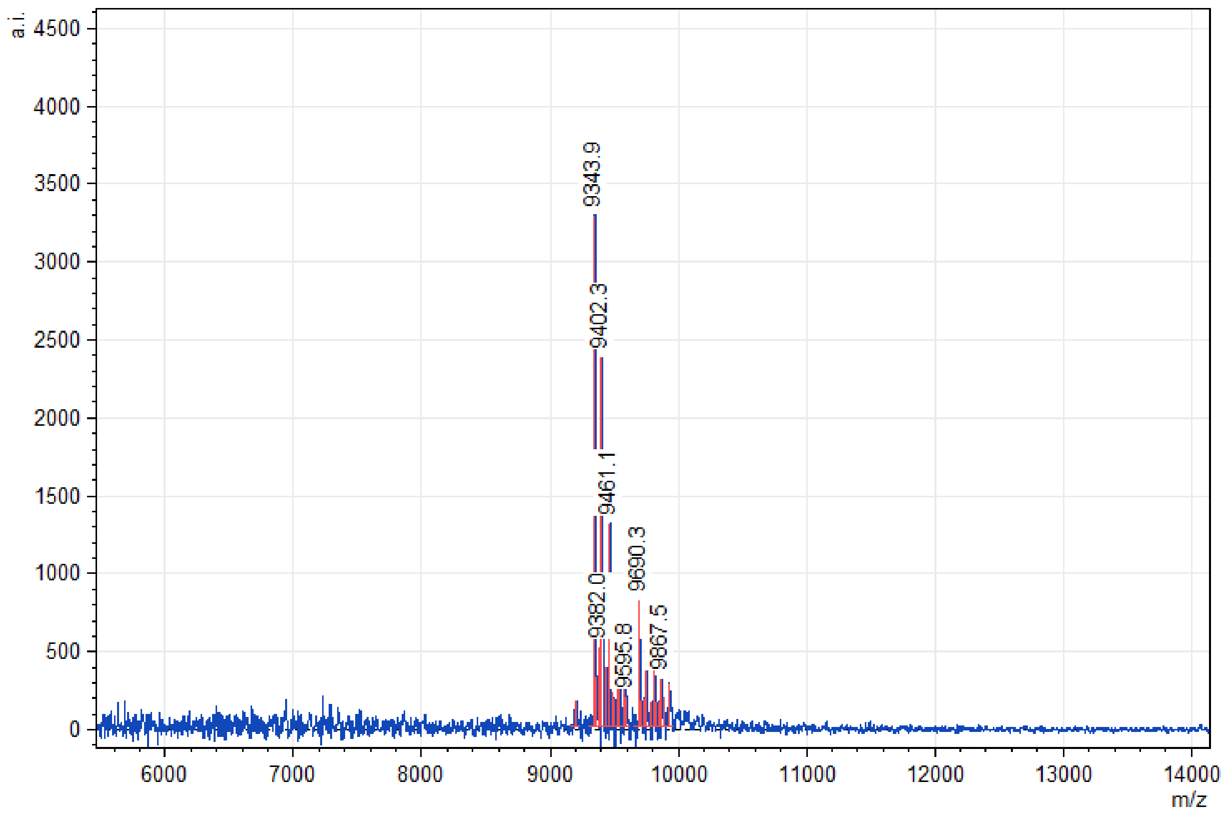

Figure S24. MALDI-TOF MS spectrum of **30DNA_T$^{\mathbf{HDO}}$ $^{\mathbf{b}}$**; Mass calculated for **30ON_T$^{\mathbf{HDO}}$** [M]: 9342.91 Da; Mass found 9343.9 Da; at m/z=9690.3 Da is assigned to the template **Temp$^{\mathbf{19_1T}}$ $^{\mathbf{b}}$**.

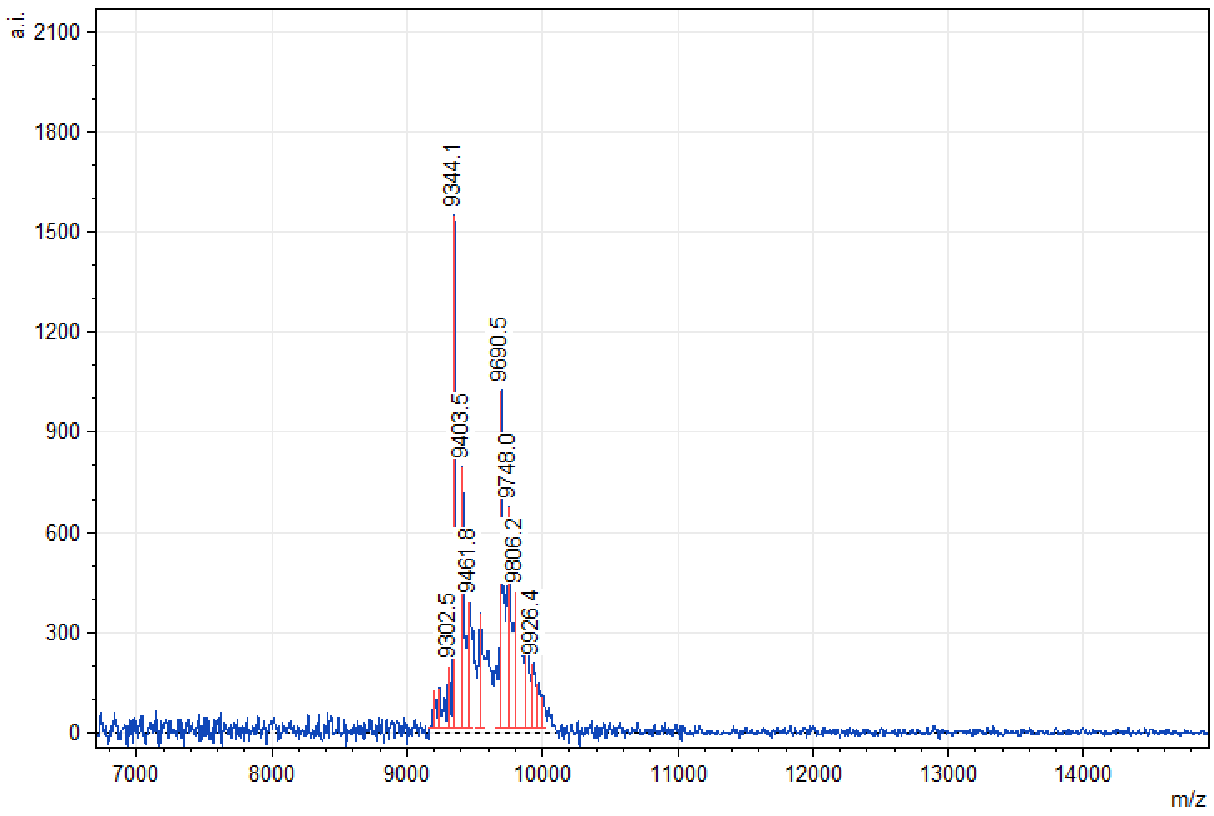

Figure S25. MALDI-TOF MS spectrum of **30DNA_T$^{PDO\ b}$**; Mass calculated for **30ON_T^{HDO}** [M]: 9342.91 Da; Mass found 9344.1 Da; at m/z=9690.3 Da is assigned to the template **Temp^{30_1T}.**

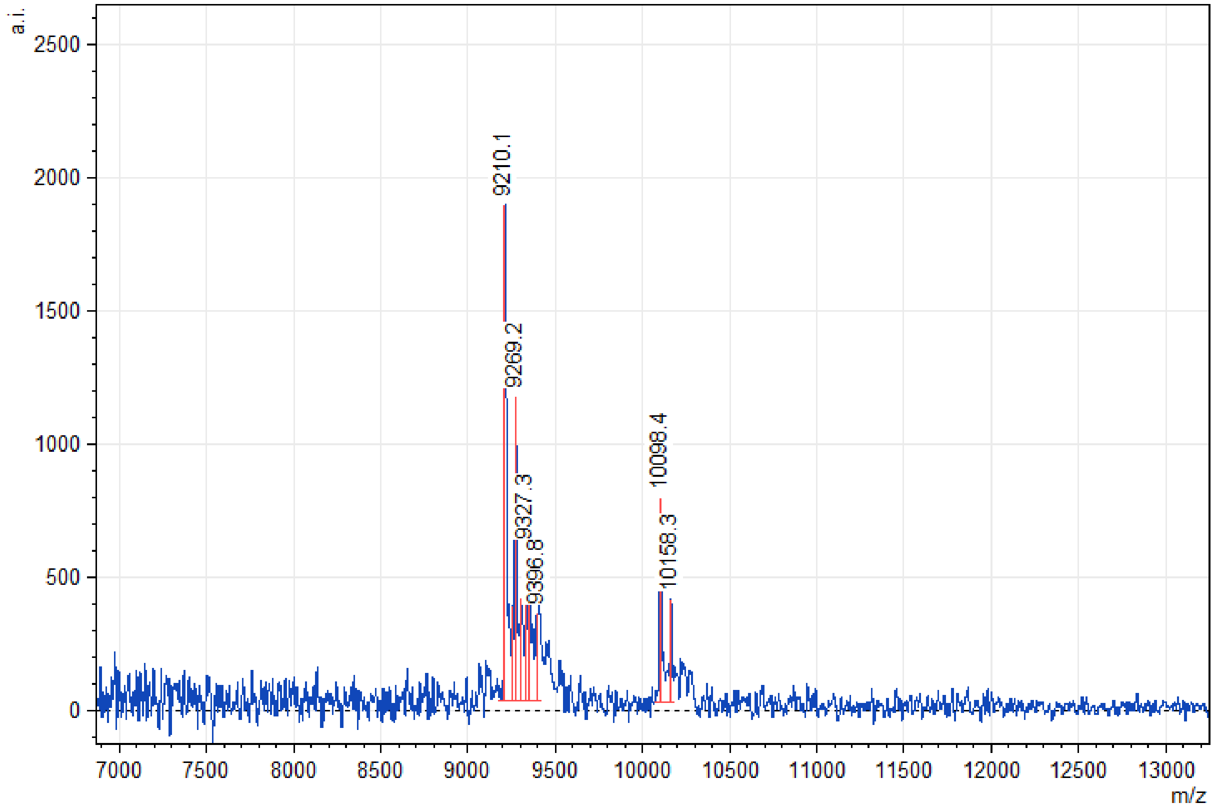

Figure S26. MALDI-TOF MS spectrum of **30DNA_2T^{HDO} [a]**; Mass calculated for **30ON_2T^{HDO} [a]** [M]: 10096.15 Da ; Mass found 10098.4 Da; The peak at m/z=9210.1 Da is assigned to the template **Temp^{30_2T}** ; The peak at m/z= 10158.3 Da is the adduct [M-2+Na+K].

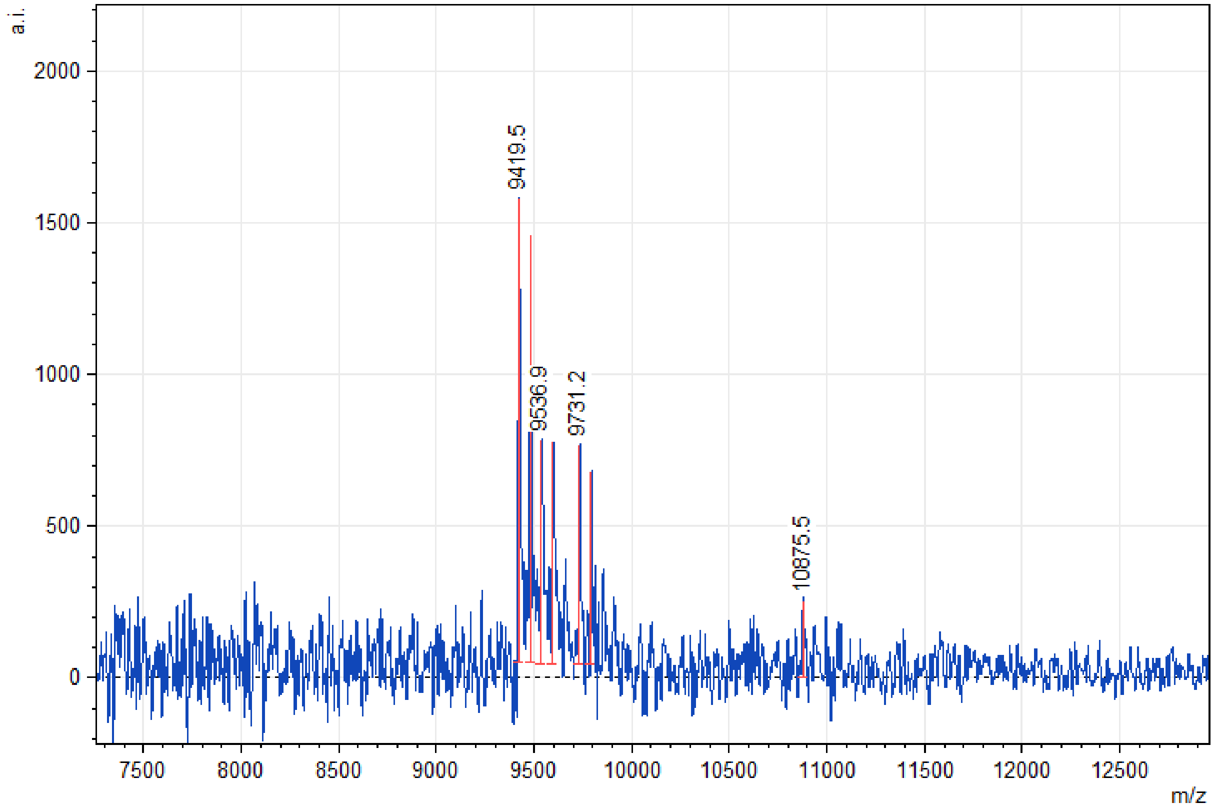

Figure S27. MALDI-TOF MS spectrum of **31DNA_4T**$^{\mathbf{HDO}}$ [a]; Mass calculated for **31ON_4T**$^{\mathbf{HDO}}$ [a] [M]: 10870.61 Da; Mass found 10875.5 Da; The peak at m/z=9419.5 Da is assigned to the template **Temp**$^{\mathbf{31\_4T}}$.

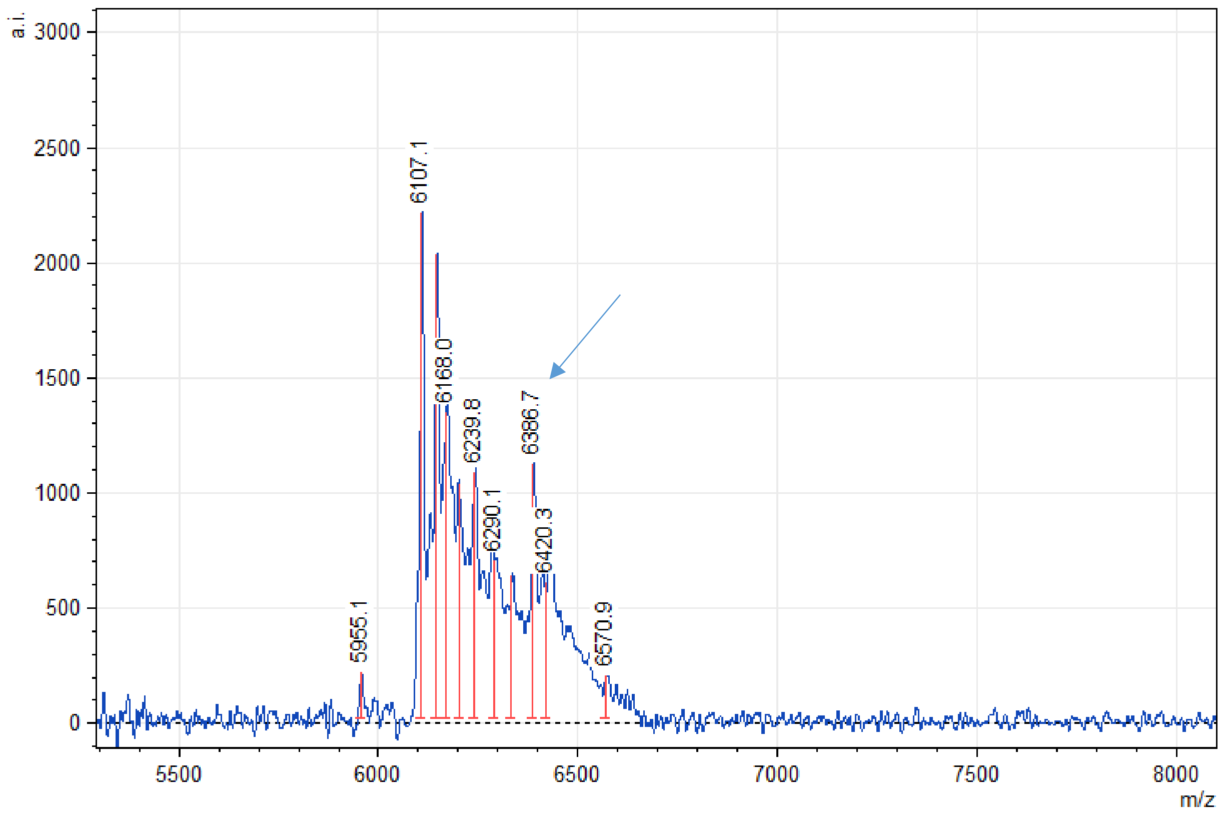

Figure S28. MALDI-TOF MS spectrum of **19DNA_T$^{\mathbf{HDOArg\ b}}$**; Mass calculated for **19ON_T$^{\mathbf{HDOArg\ b}}$** [M]: 6383.12 Da; Mass found 6386.7 Da; The peak at m/z=6107.1 Da is assigned to the template **Temp$^{\mathbf{19_1T\ b}}$** .

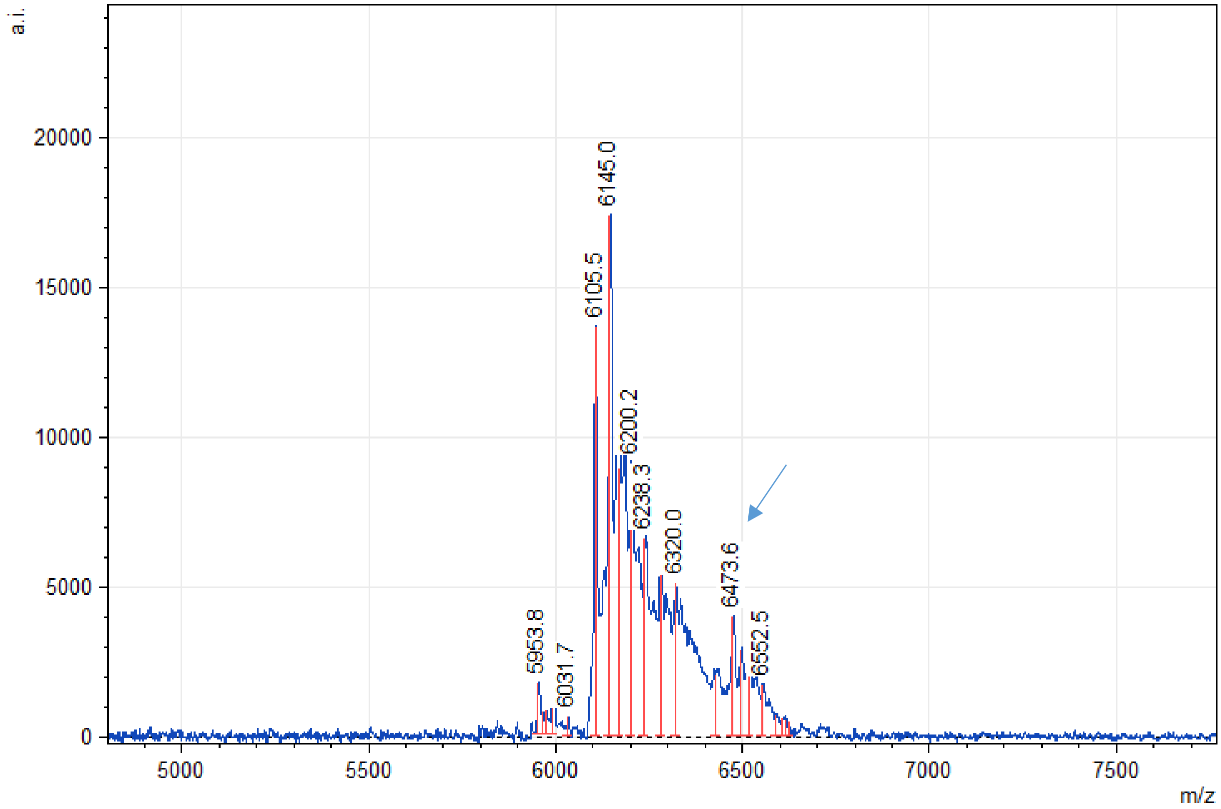

Figure S29. MALDI-TOF MS spectrum of **19DNA_T^{HDOARA} ^{b}**; Mass calculated for **19ON_T^{HDOARA}** [M]: 6467.15 Da; Mass found 6473.6 Da; The peak at m/z=6105.5 Da is assigned to the template **Temp$^{19_1T\ b}$** .

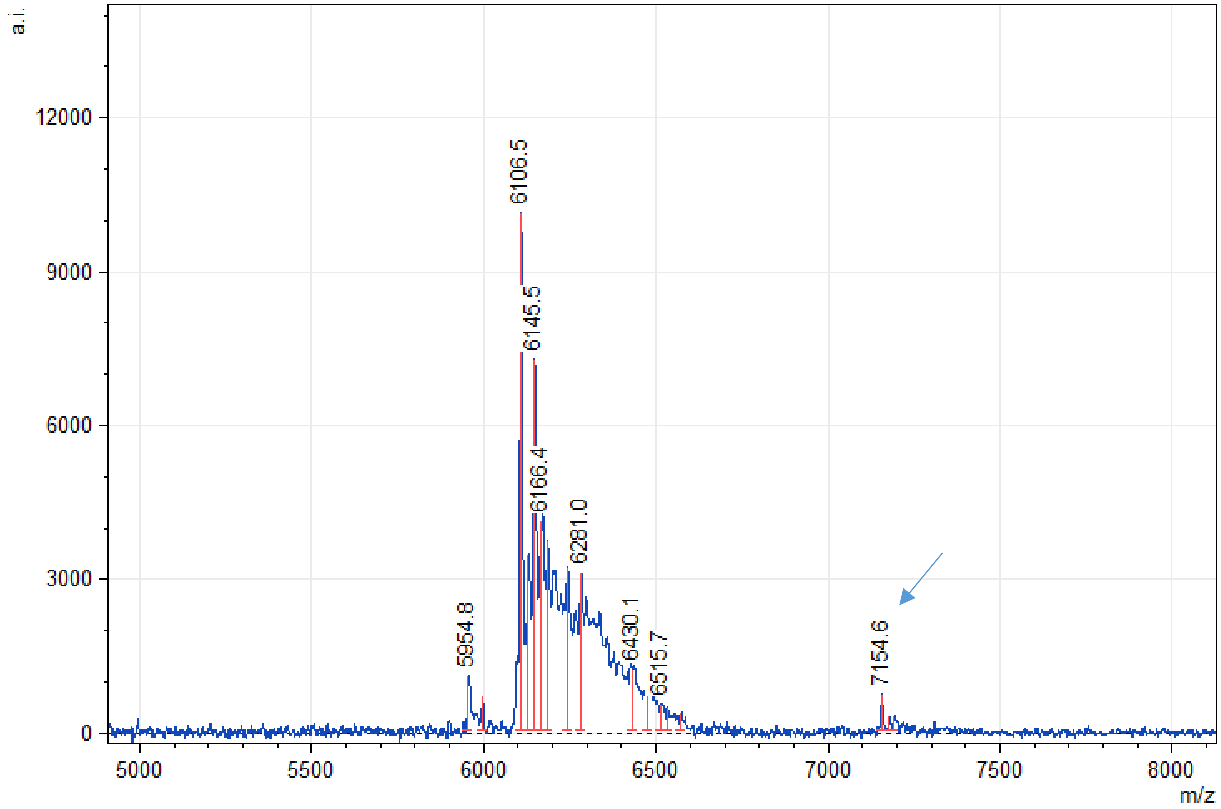

Figure S30. MALDI-TOF MS spectrum of **19DNA_T$^{HDO10pept\ b}$**; Mass calculated for **30ON_T^{HDO10pept}** [M]: 7147.44 Da; Mass found 7154.6 Da; The peak at m/z=6106.5 Da is assigned to the template **Temp$^{19_1T\ b}$**.

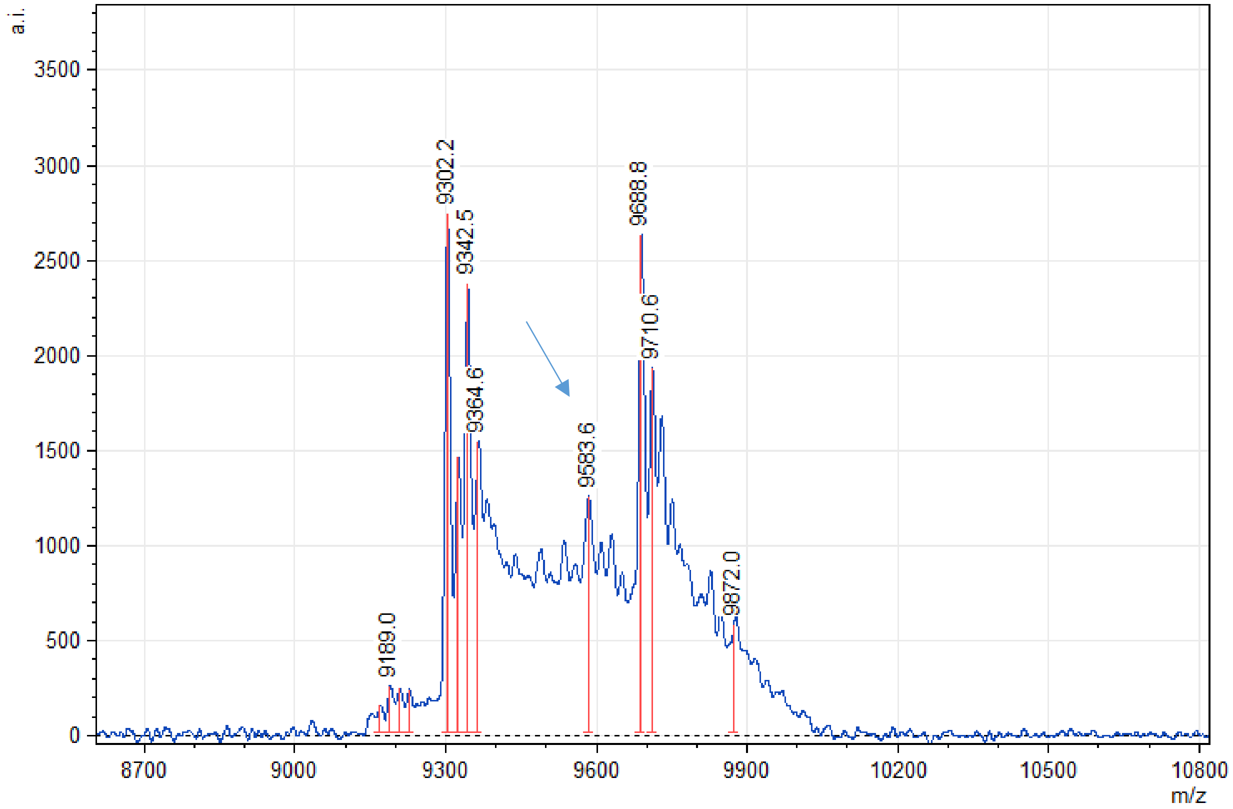

Figure S31. MALDI-TOF MS spectrum of **30DNA_T$^{HDO^{Arg}}$ b**; Mass calculated for **30ON_T$^{HDO^{Arg}}$ b** [M]: 9580.22 Da; Mass found 9583.6 Da; The peak at m/z=9688.8 Da is assigned to the template **Temp^{30_1T} b**.

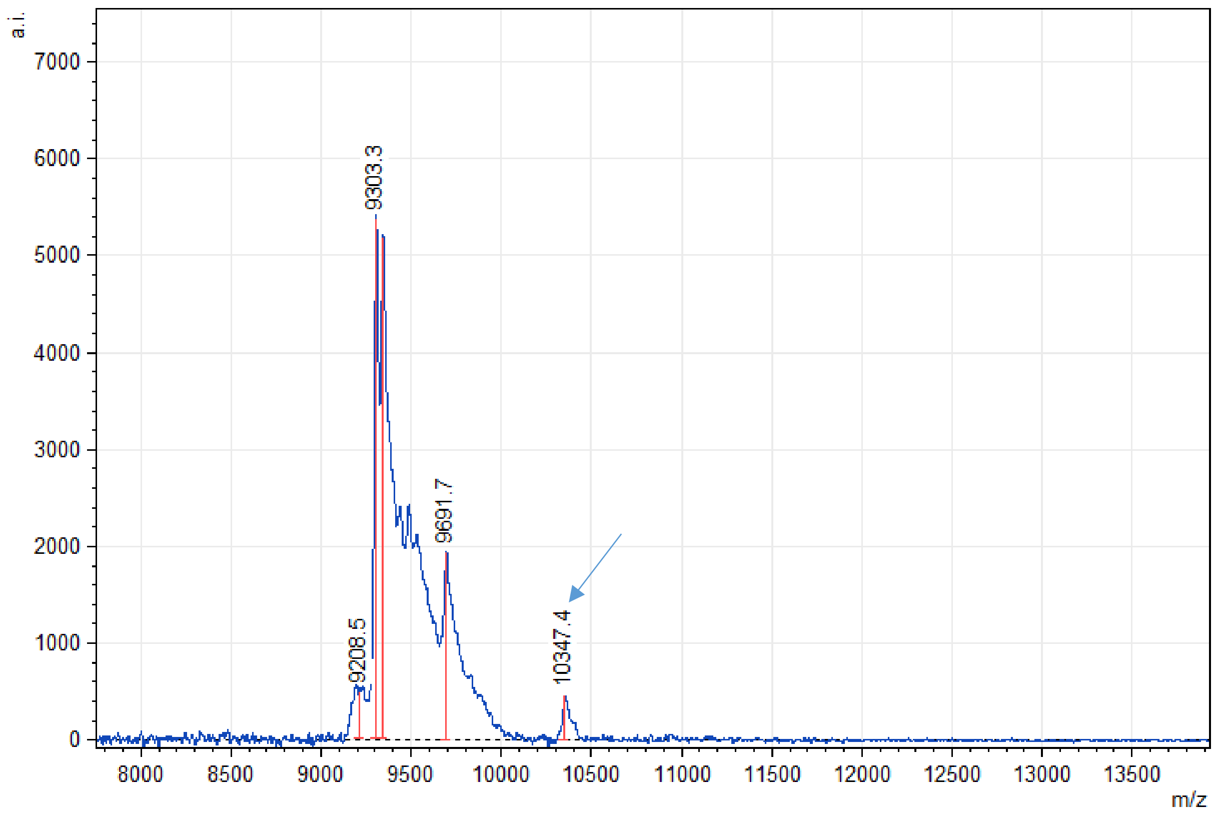

Figure S32. MALDI-TOF MS spectrum of **30DNA_T$^{HDO10pept\ b}$**; Mass calculated for **30ON_T^{HDO10pept}** [M]: 10344.48 Da; Mass found 10347.4 Da; The peak at m/z=9691.7 Da is assigned to the template **Temp$^{30_1T\ b}$**.

a. ESI-spectra

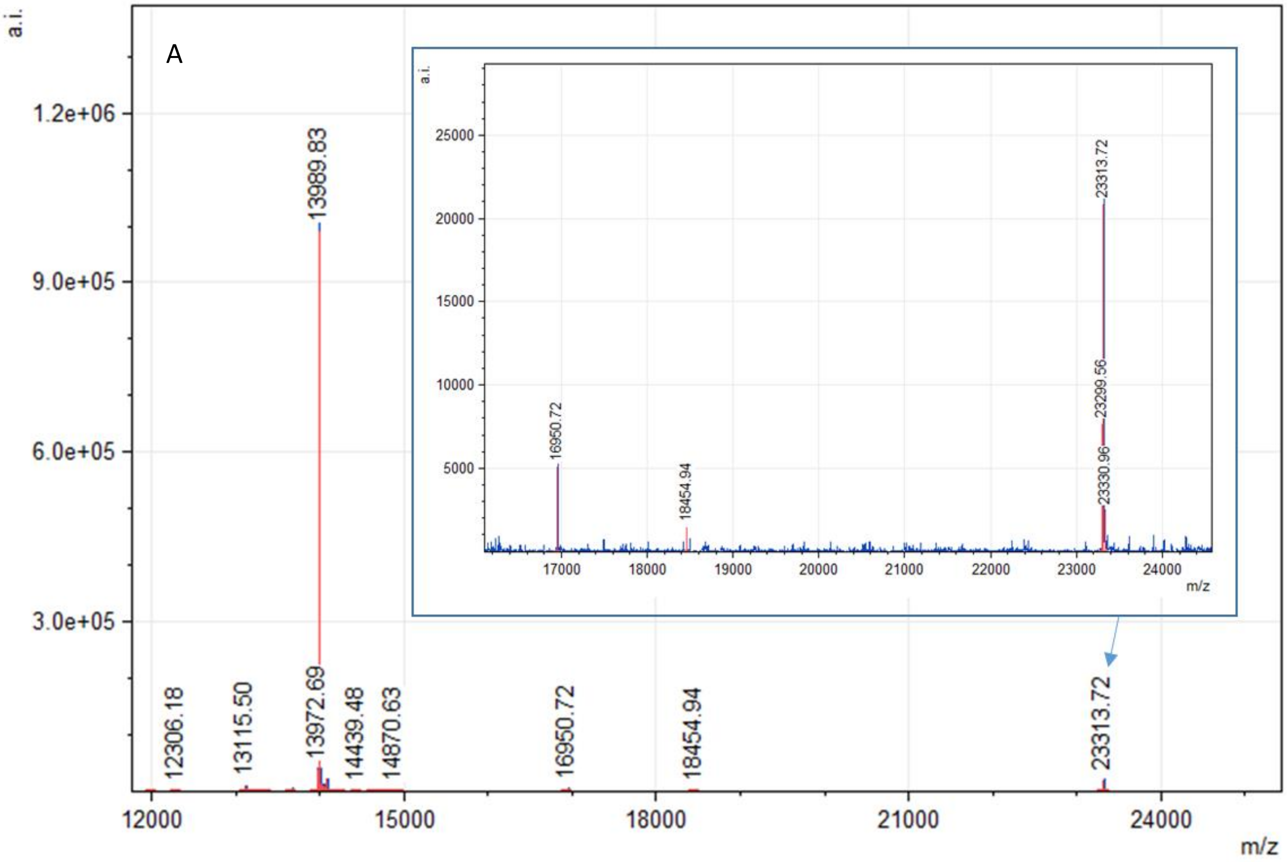

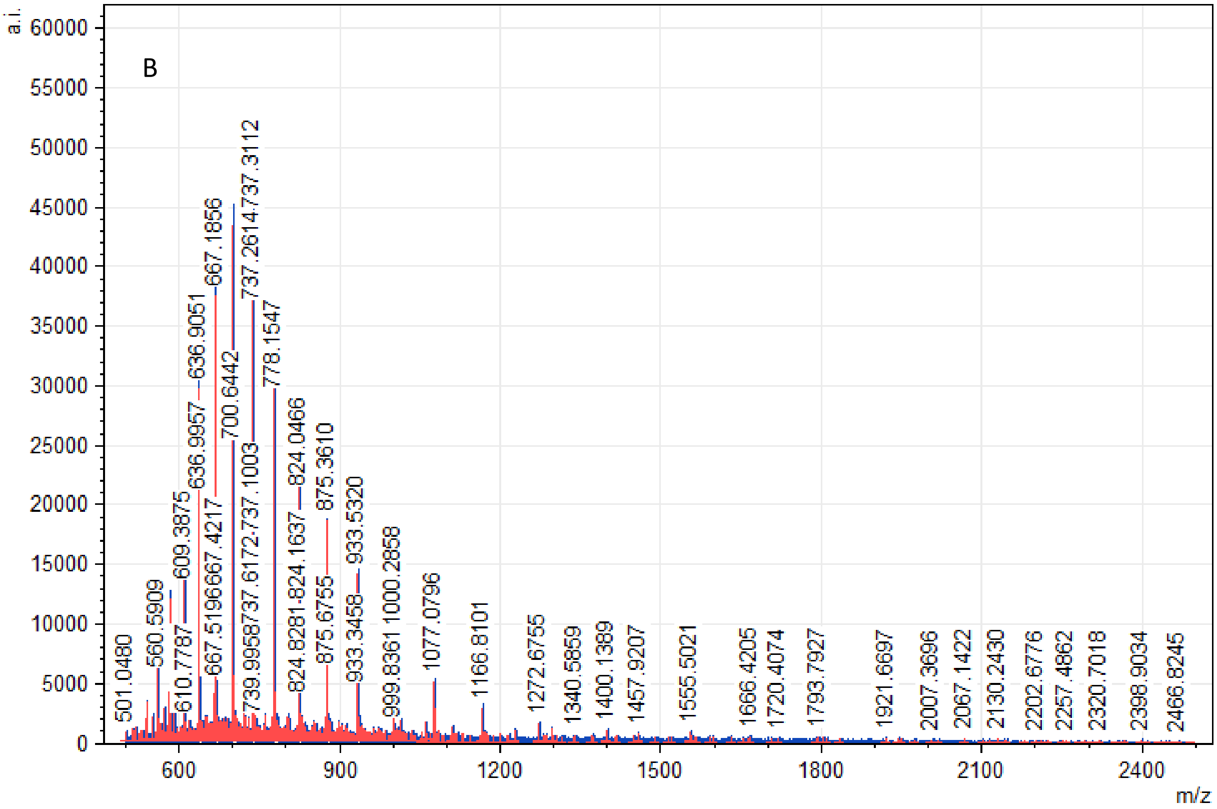

Figure S33. A) Deconvoluted Mass spectrum of **30DNA_T**$^{\mathbf{HDO\_H2A\ b}}$; Mass calculated for **30ON_T**$^{\mathbf{HDO\ \_H2A}}$ [M]: 23297.3 Da; Mass found: 23313.72 Da; the peak at m/z= 13989.83 Da is assigned to histone H2A. B) Raw spectrum.

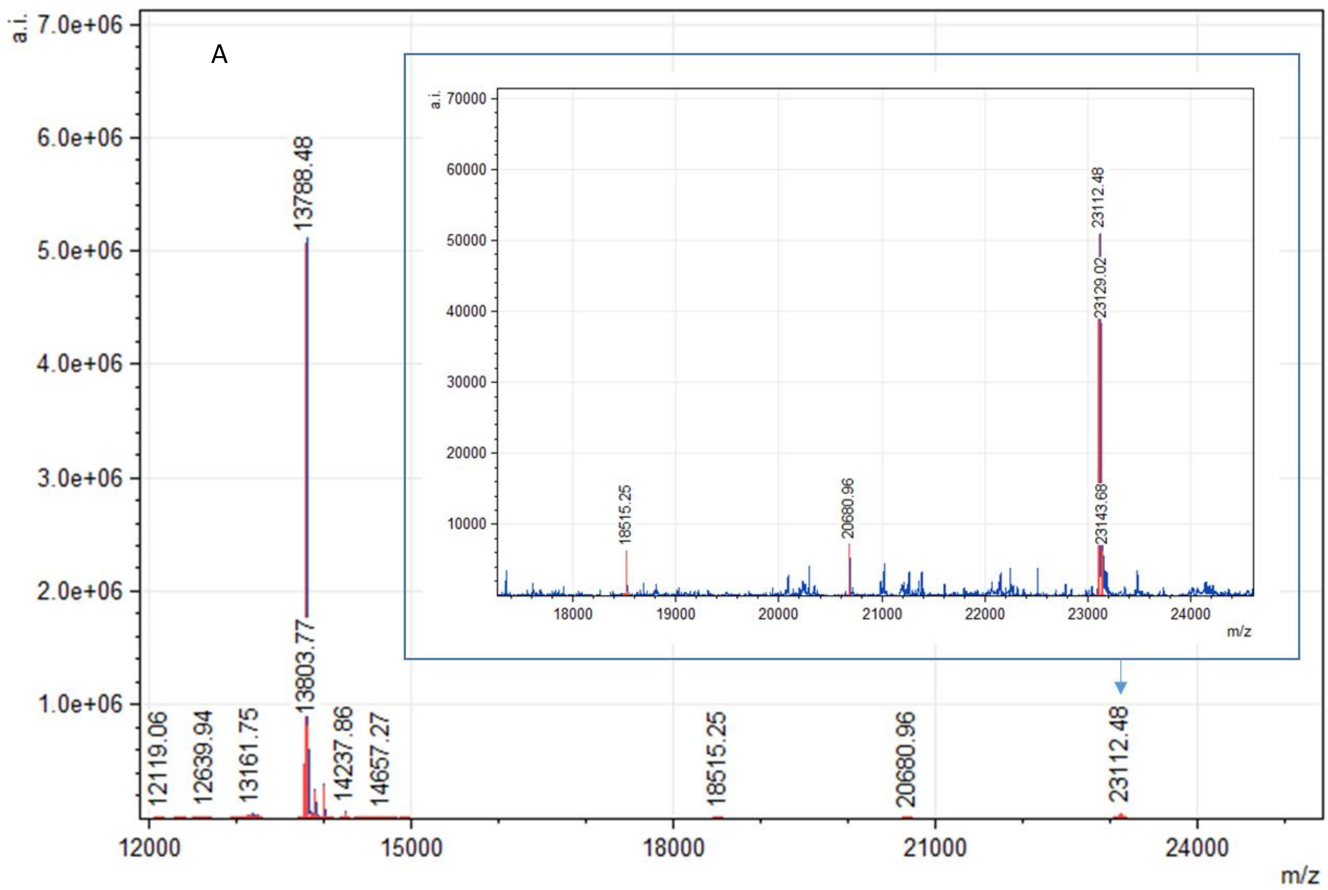
A
a.i.
7.0e+06
6.0e+06
5.0e+06
4.0e+06
3.0e+06
2.0e+06
1.0e+06
13788.48
13803.77
12119.06
12639.94
13161.75
14237.86
14657.27
18515.25
20680.96
23112.48
12000
15000
18000
21000
24000
m/z
70000
60000
50000
40000
30000
20000
10000
23129.02
23143.68
19000
20000
22000
23000

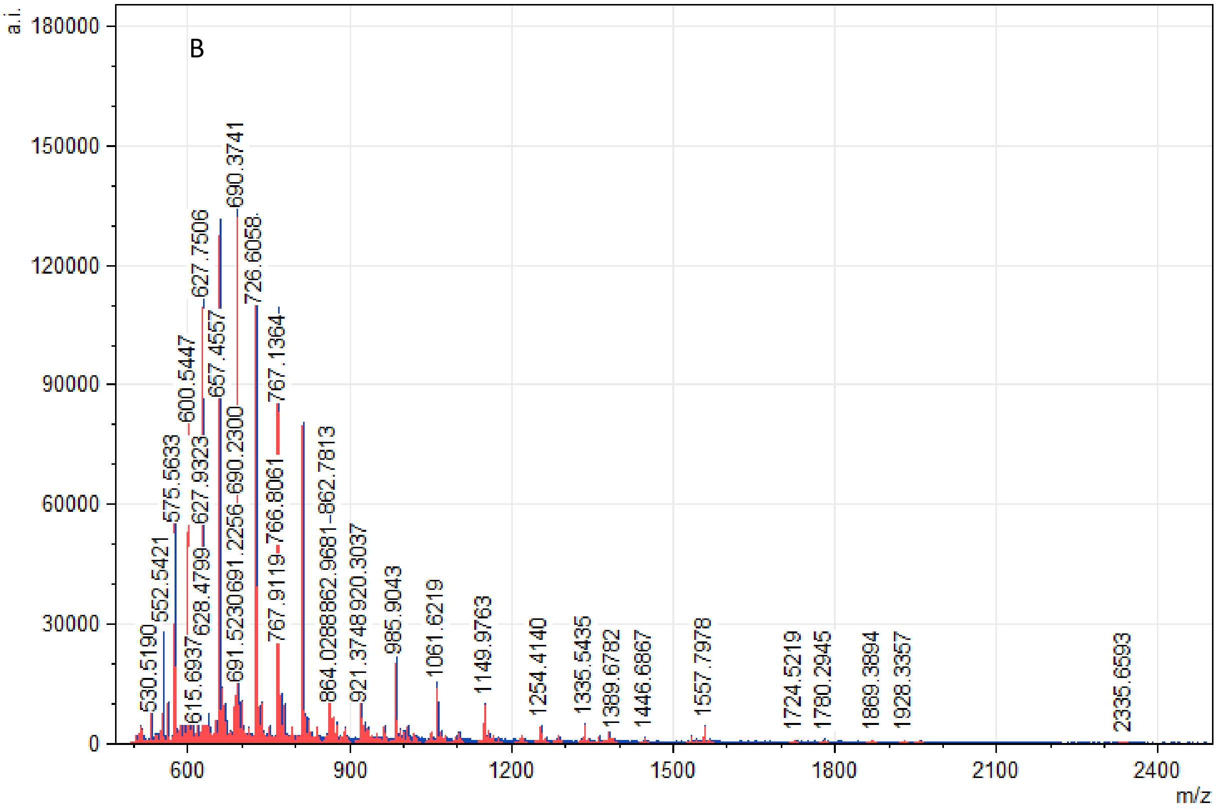

Figure S34. A) Deconvoluted Mass spectrum of **30DNA_T**$^{\mathbf{HDO\_H2B}\ \mathbf{b}}$; Mass calculated for **30ON_T**$^{\mathbf{HDO\ \_H2B}}$ [M]: 23097.0 Da; Mass found : 23112.48 Da; the peak at m/z= 13788.48 is assigned to histone H2B. B) Raw spectrum.

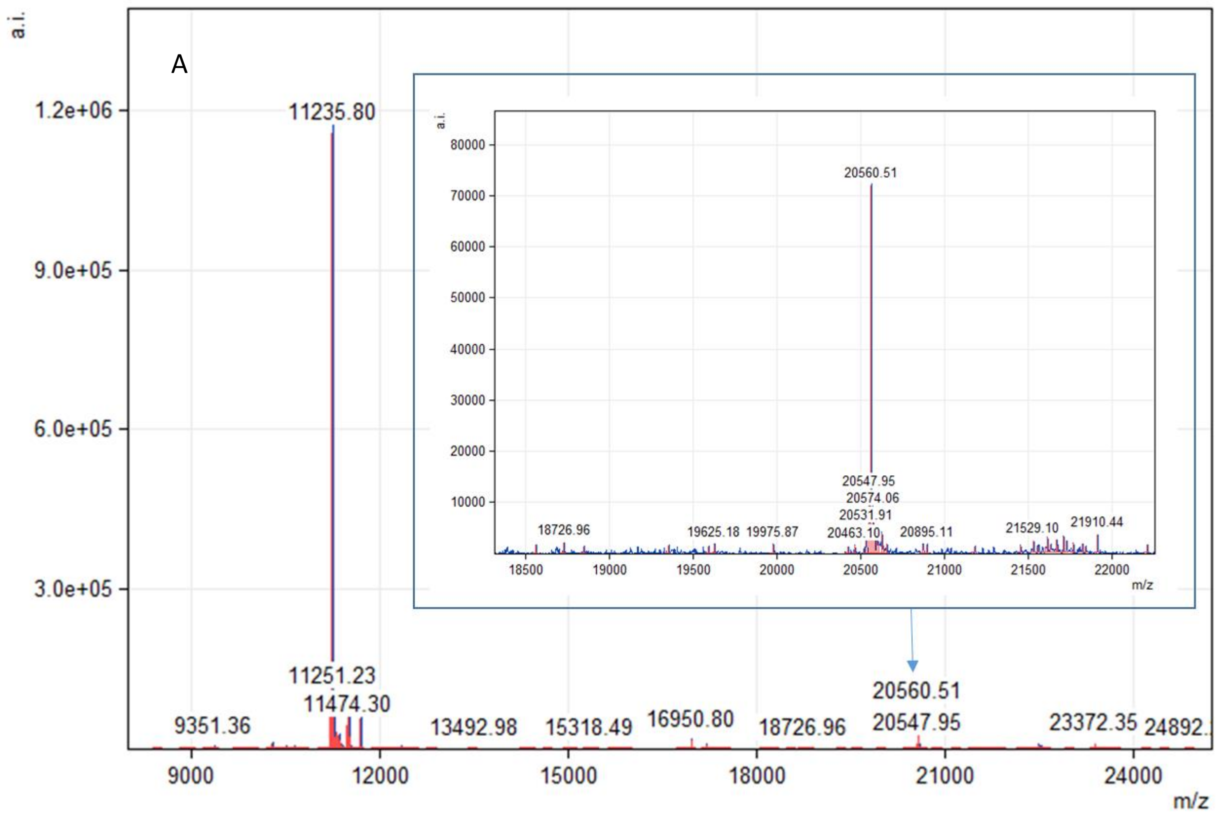

A
a.i.
1.2e+06
9.0e+05
6.0e+05
3.0e+05
11235.80
11251.23
11474.30
9351.36
13492.98
15318.49
16950.80
18726.96
20560.51
20547.95
23372.35
9000
12000
15000
18000
21000
24000
m/z
80000
70000
60000
50000
40000
30000
20000
10000
20560.51
20547.95
20574.06
20531.91
20463.10
18726.96
19625.18
19975.87
20895.11
21529.10
21910.44
18500
19000
19500
20000
20500
21000
21500
22000

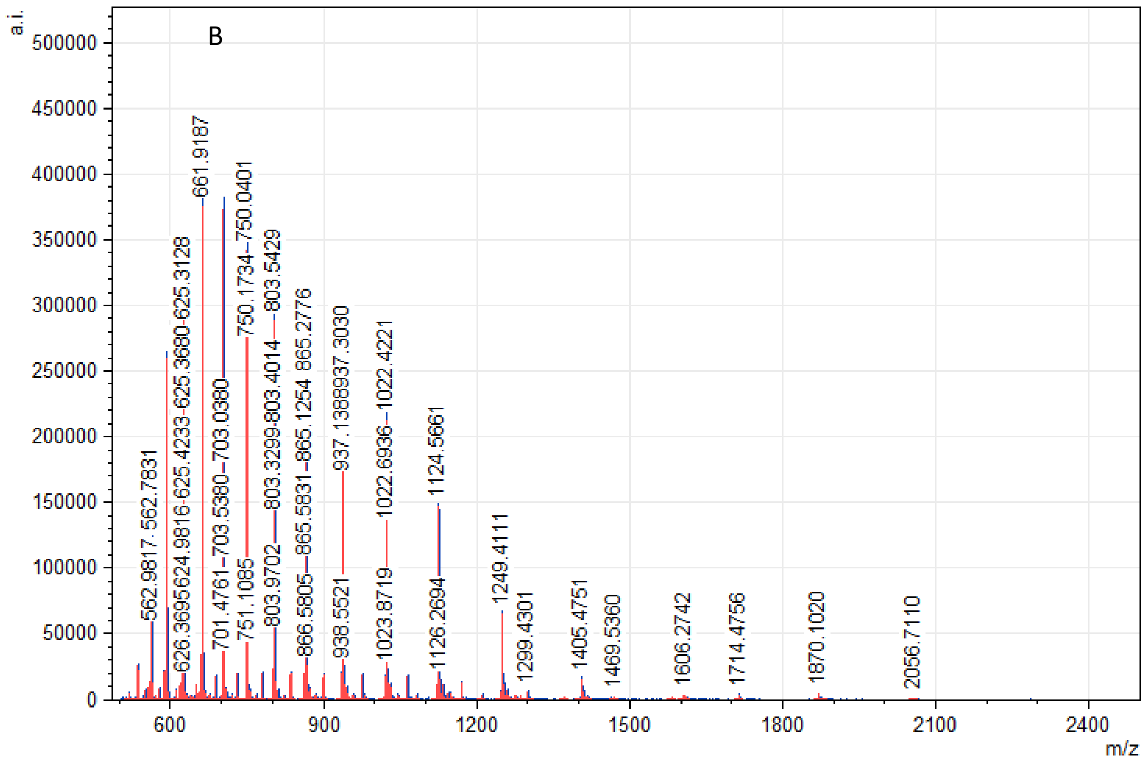

Figure S35. A) Deconvoluted Mass spectrum of **30DNA_T$^{HDO_H4\,b}$**; Mass calculated for **30ON_T^{HDO_H4}** [M]: 20547.0 Da; Mass found: 20560.51 Da; the peak at m/z= 11235.8 Da is assigned to histone H4. B) Raw spectrum.

4. Copies of HPLC chromatograms

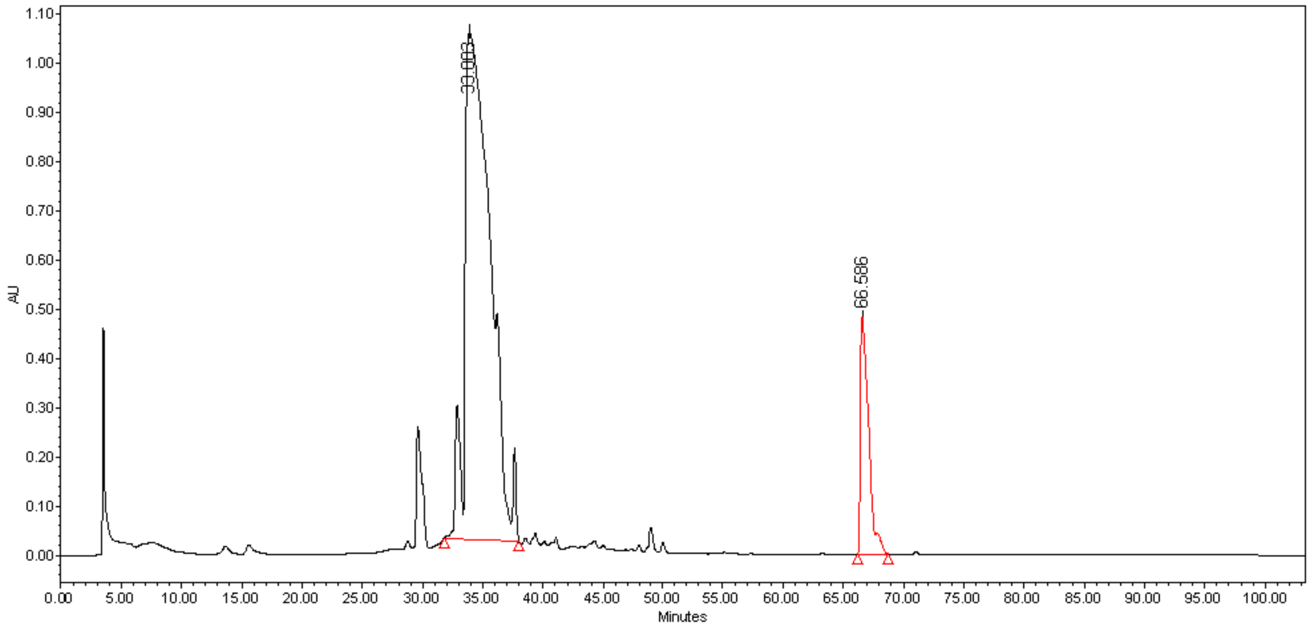

Figure S36. HPLC chromatogram of cross-linking reaction of **dT^{HDO}MP** with N-BocArg-OH (3 equiv) in $NaHCO_3$ buffer pH 10 (18 h). **dT^{HDO}MP** Rt = 33.8 min., **dT^{HDOArg}MP** Rt = 66.5 min. Column Luna Omega, 5µm Polar Column 150x 21.2 mm. Gradient form 0.1% TEAB to 100% MeOH.

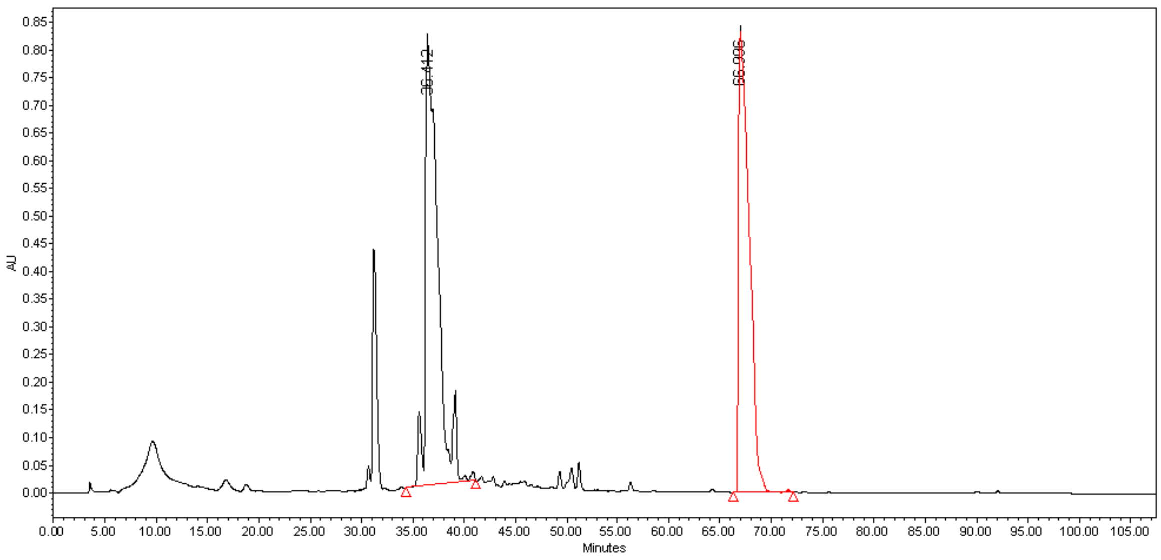

Figure S37. HPLC chromatogram of cross-linking reaction of $\mathbf{dT^{HDO}MP}$ with N-BocArg-OH (3 equiv.) in $NaHCO_3$ buffer pH 10 (89 h). $\mathbf{dT^{HDO}MP}$ Rt = 36.4 min, $\mathbf{dT^{HDOArg}MP}$ Rt = 66.5 min. Column Luna Omega, 5µm Polar Column 150x 21.2 mm. Gradient form 0.1% TEAB to 100% MeOH.

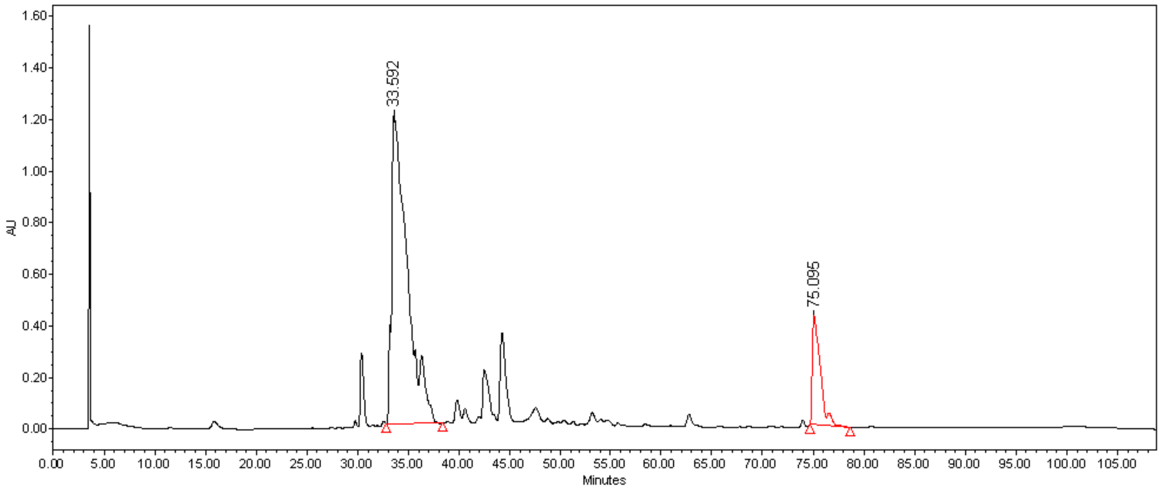

Figure S38. HPLC chromatogram of cross-linking reaction of $\mathbf{dT^{HDO}MP}$ with N-BocAlaArgAla-OH (3 equiv.) in NaHCO3 buffer pH 10 (89 h). $\mathbf{dT^{HDO}MP}$ Rt = 33.6 min, $\mathbf{dT^{HDOARA}}$MP Rt = 75.0 min. Column Luna Omega, 5µm Polar Column 150x 21.2 mm. Gradient from 0.1% TEAB to 100% MeOH.

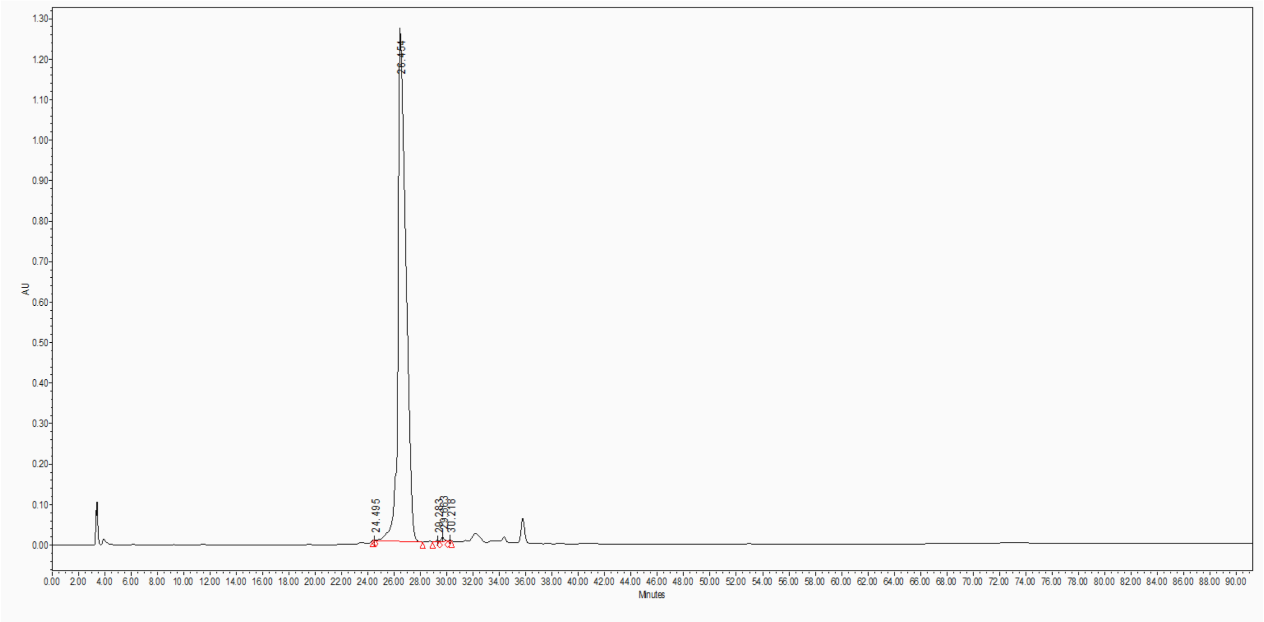

Figure S39 HPLC chromatogram of standard **dT^HDO^MP** Rt=26.4 min. Column X-Bridge Prep. RP 18, 5 μm DBD, 19x150 mm. Gradient from 0.1% TEAB to 100% MeOH.

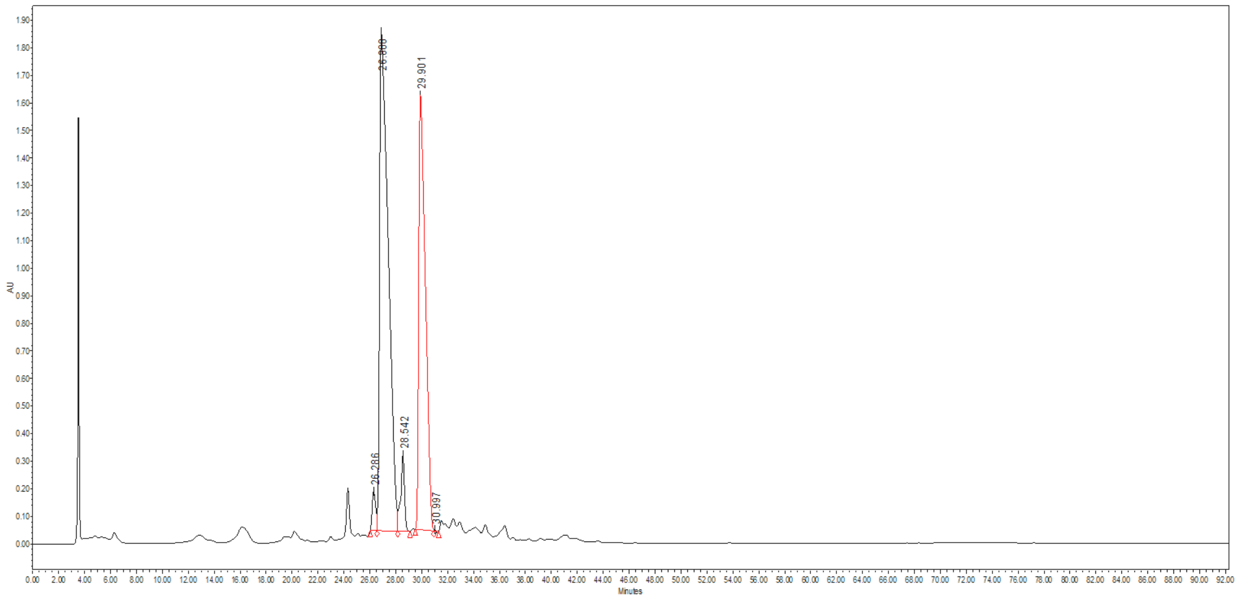

Figure S40 HPLC chromatogram of reaction of **dT^HDO^MP** with NH_2-AlaGlyAla-OH (3 equiv.) in $NaHCO_3$ buffer pH 10. **dT^HDO^MP** Rt = 26.00 min. and **dT^HDOAGA^MP** Rt = 29.00 min. Column X-Bridge Prep. RP 18.5μm DBD, 19x150 mm. Gradient from 0.1% TEAB to 100% MeOH.

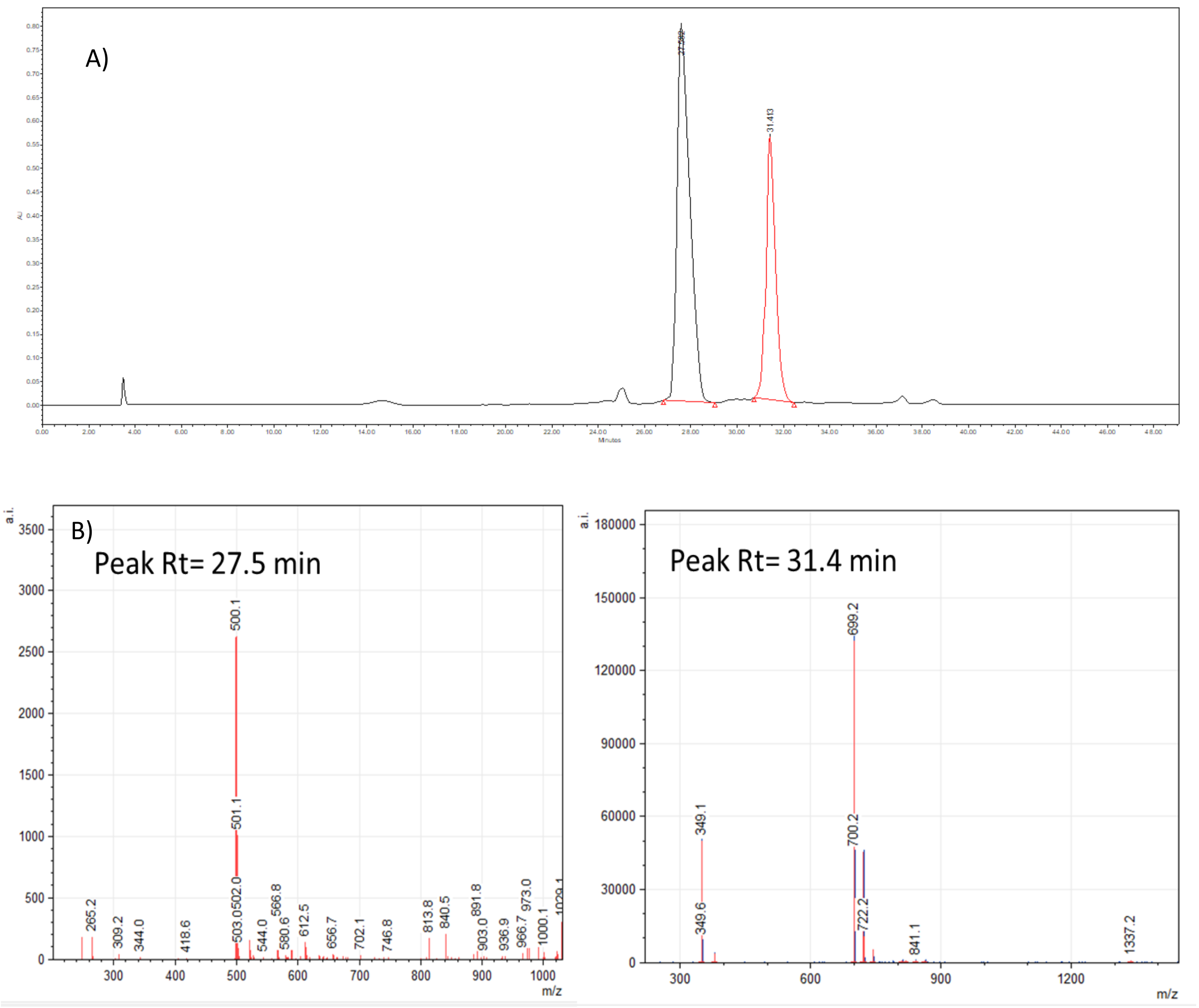

Figure S41 A) HPLC chromatogram of stability study on **dT^{HDOAGA}MP** in H_2O at 25°C for 20 h**.** **dT^{HDO}MP** Rt = 27.5 min, **dT^{HDOAGA}MP** Rt = 31.4 min (23% isolated). Column X-Bridge Prep. RP 18, 5µm DBD, 19x150 mm. Gradient from 0.1% TEAB to 100% MeOH. B) ESI-spectra of the peak Rt = 27.5 min and at Rt = 31.4 min.

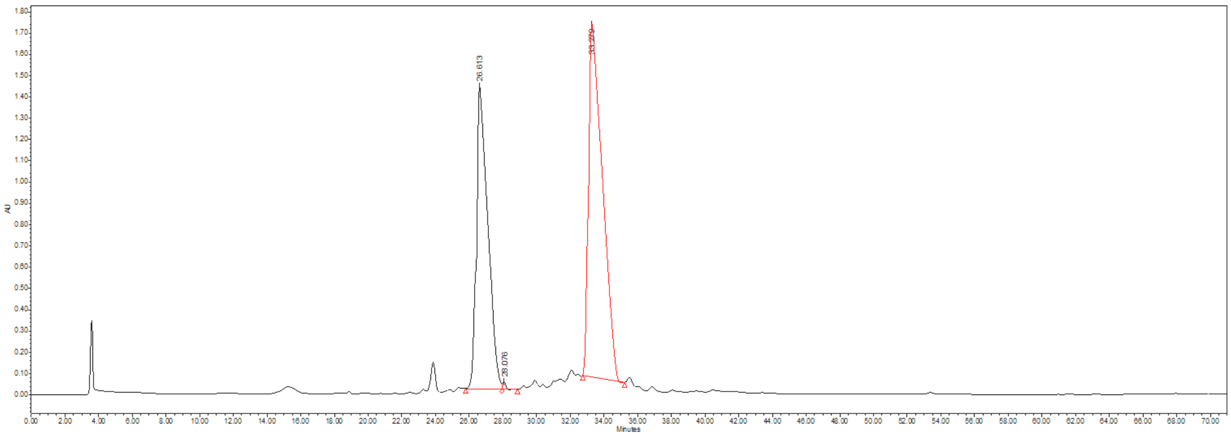

Figure S42. HPLC chromatogram of the reaction of **dT^HDO^MP** with tripeptide Ac-AlaLysAla-OH (3equiv.) in $NaHCO_3$ buffer pH 10. **dT^{HDO}MP** Rt = 26.6 min and **dT^{HDOAKA}MP** Rt = 33.2 min. Column X-Bridge Prep. RP 18.5µm DBD, 19x150 mm. Gradient from 0.1% TEAB to 100% MeOH.

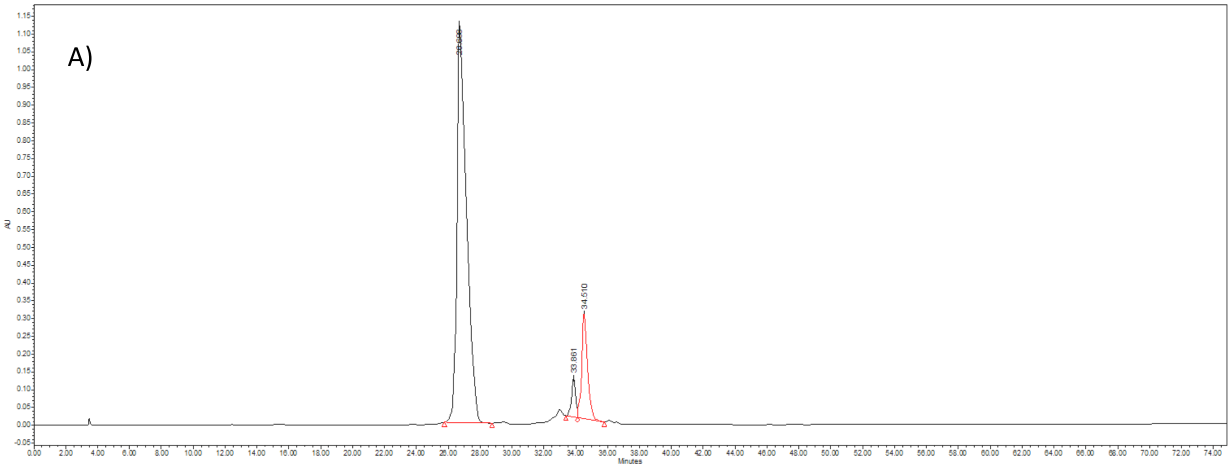

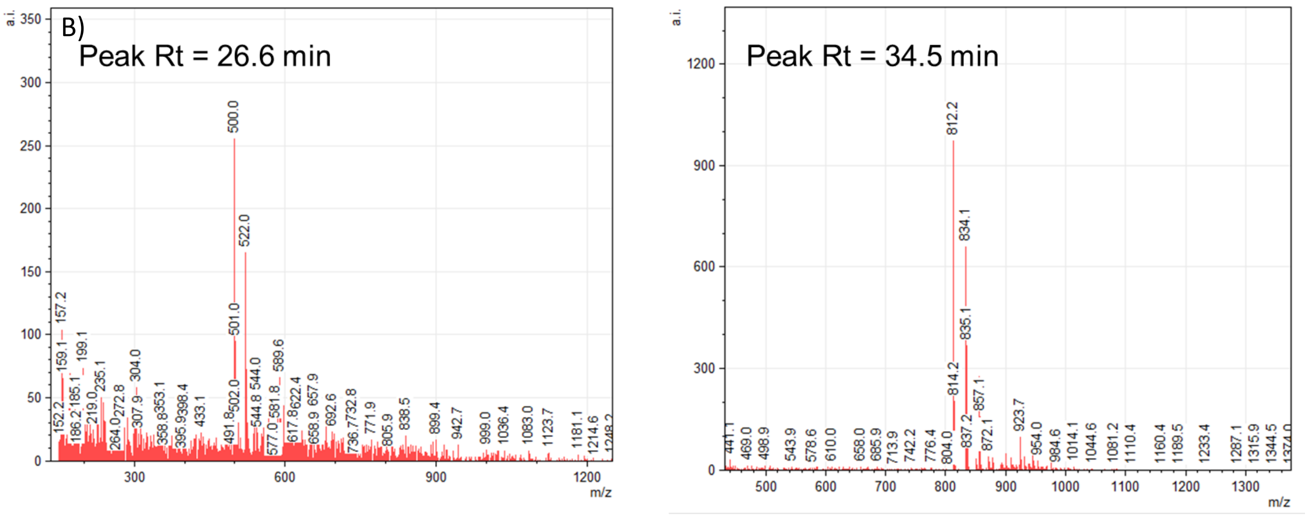

Figure S43. A) HPLC chromatogram of stability study on **dT^{HDOAKA}MP** in H_2O at 25°C for 20 h**.** **dT^{HDO}MP** Rt = 26.6 min**, dT^{HDOAKA}MP** Rt = 34.5 min (13% isolated). Column X-Bridge Prep. RP 18.5µm DBD, 19x150 mm. Gradient from 0.1% TEAB to 100% MeOH. B) ESI- spectra of the peak at 26.6 min and for the peak Rt = 34.5 min.

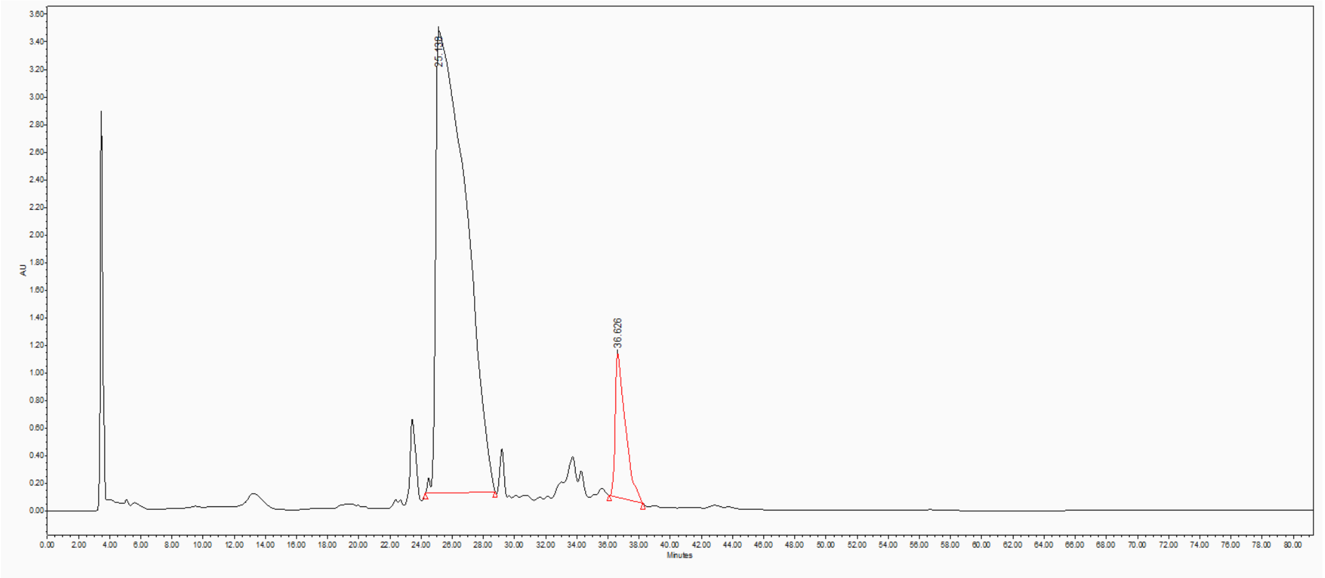

Figure S44. HPLC chromatogram of the reaction of **dT^{HDO}MP** with tripeptide Ac-AlaArgAla-OH (3 equiv.) in $NaHCO_3$ buffer pH 10. **dT^{HDO}MP** Rt = 25.1 min and **dT^{HDOAGA}MP** Rt = 36.6 min. Column X-Bridge Prep. RP 18.5µm DBD, 19x150 mm. Gradient from 0.1% TEAB to 100% MeOH.

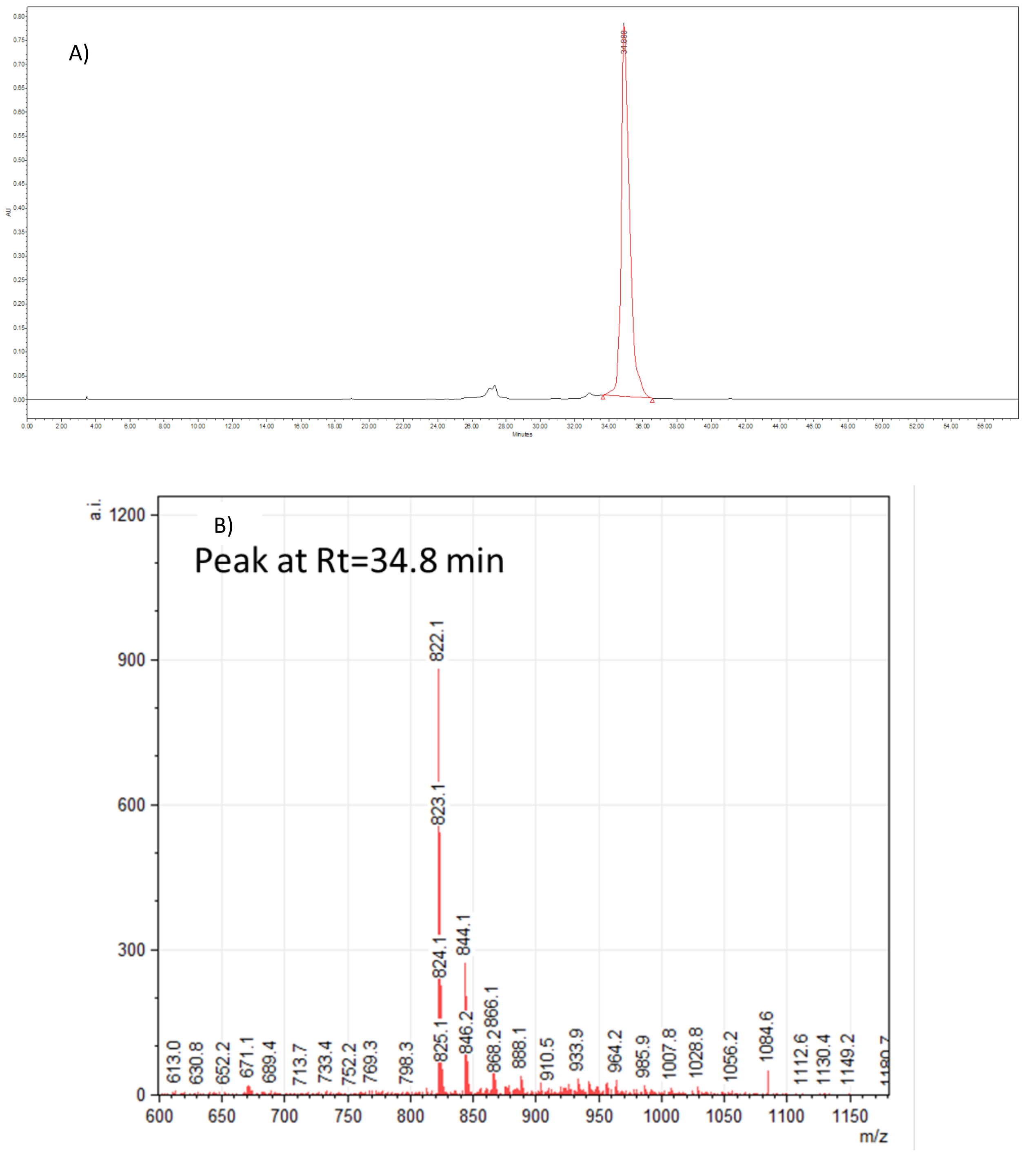

Figure S45. A) HPLC chromatogram of stability study of **dT^{HDOARA}MP** in H_2O at 25°C for 20h**.** **dT^{HDOARA}MP** Rt = 34.8 min (95% isolated). Column X-Bridge Prep. RP 18.5µm DBD, 19x150 mm. Gradient from 0.1% TEAB to 100% MeOH. B) ESI- spectra of the peak Rt = 34.8 min.

5. Copies of NMR Spectra:

^{1}H, ^{13}C, ^{31}P Spectra of 3-(prop-2-yn-1-yl) pentane-2, 4-dione (PDO):

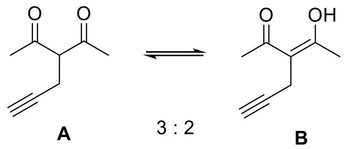

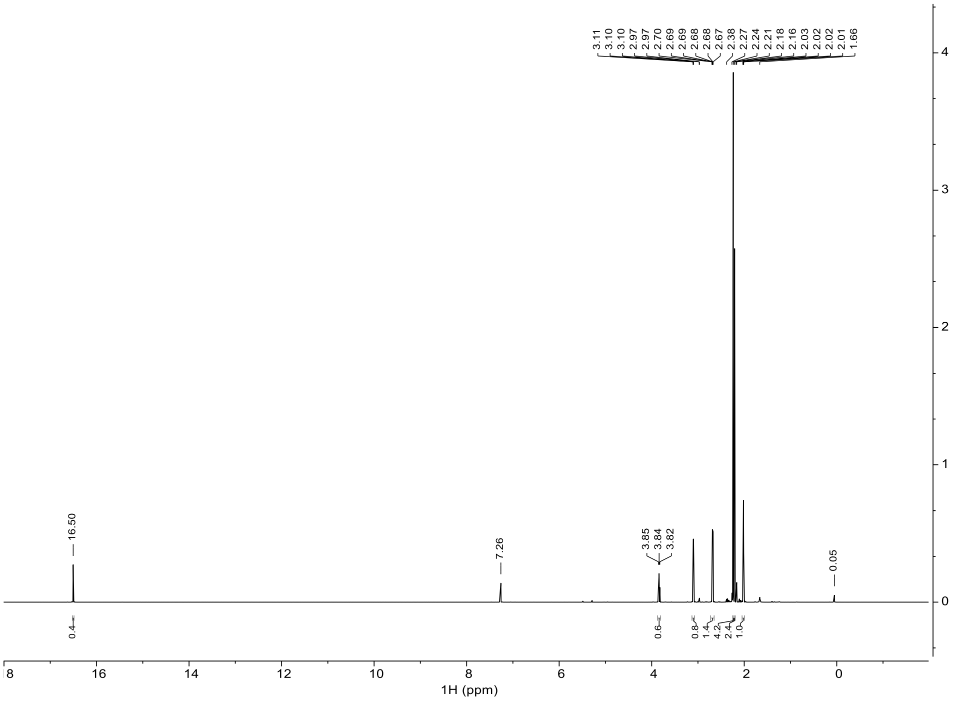

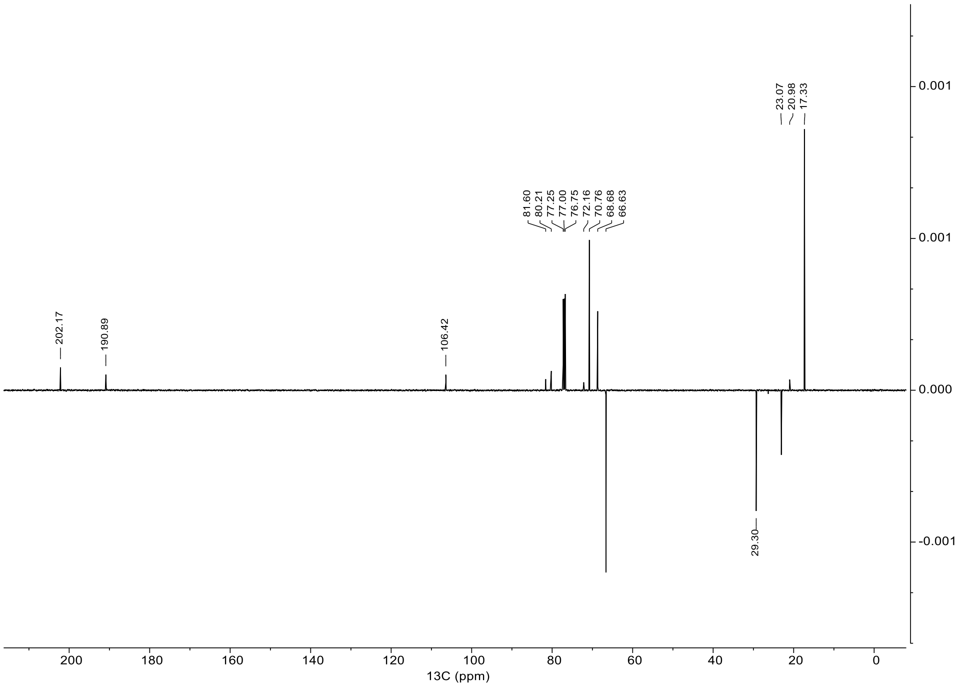
202.17
190.89
106.42
81.60
80.21
77.25
77.00
76.75
72.16
70.76
68.68
66.63
29.30
23.07
20.98
17.33
0.001
0.001
0.000
-0.001
200
180
160
140
120
100
80
60
40
20
0
13C (ppm)

^{1}H, ^{13}C, ^{31}P Spectra of dT^{HDO}MP

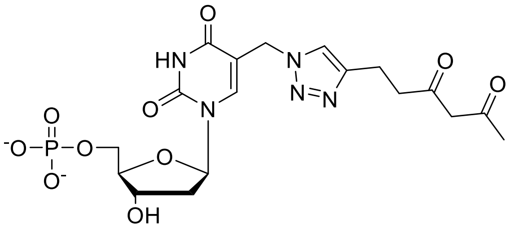

2Et_3NH^+

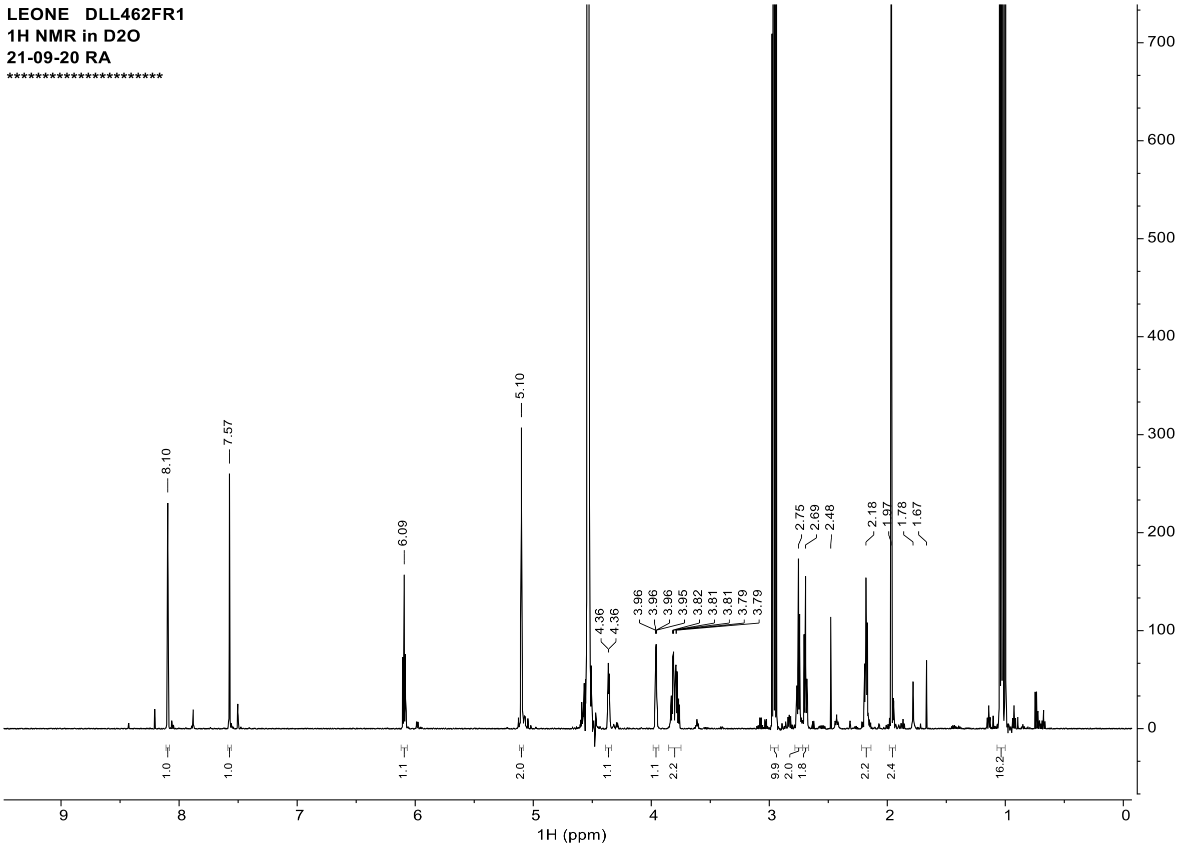

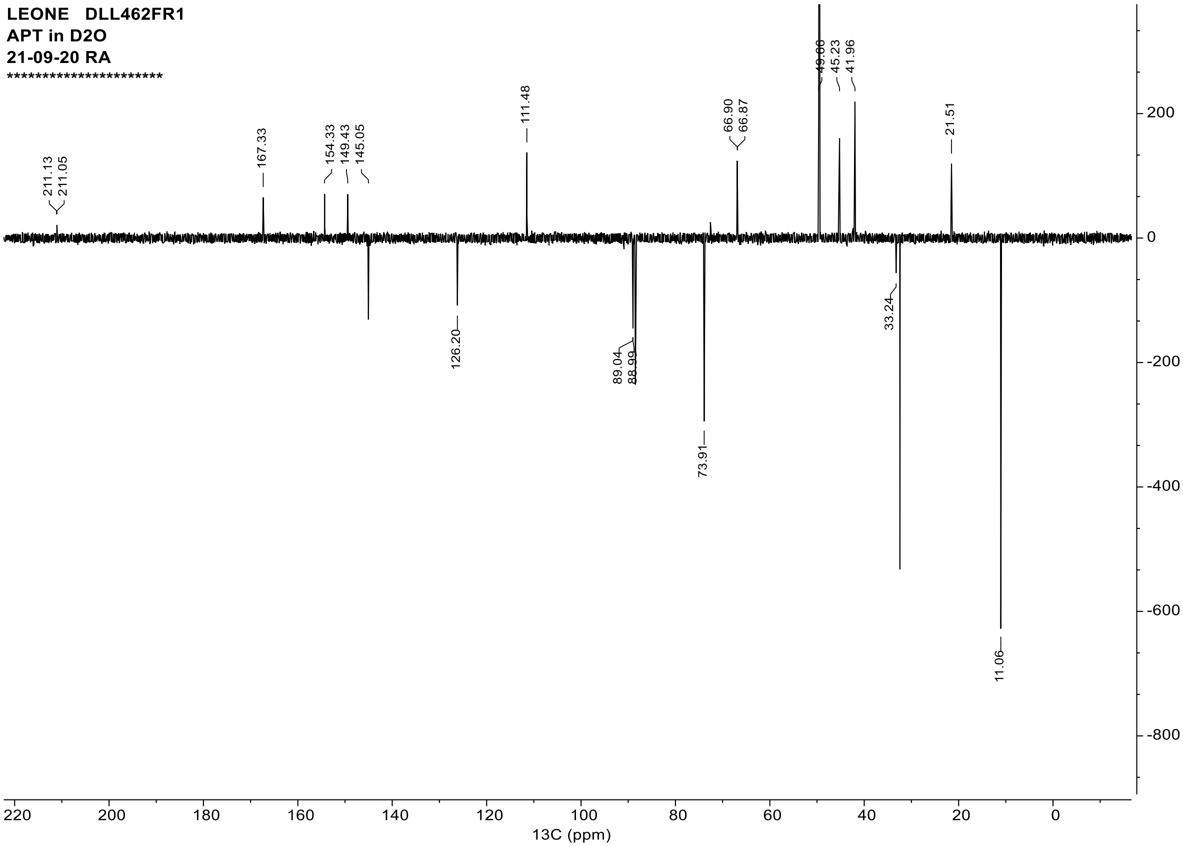
LEONE DLL462FR1
APT in D2O
21-09-20 RA
****
211.13
211.05
167.33
154.33
149.43
145.05
111.48
126.20
89.04
88.99
73.91
66.90
66.87
45.23
41.96
33.24
21.51
11.06
13C (ppm)

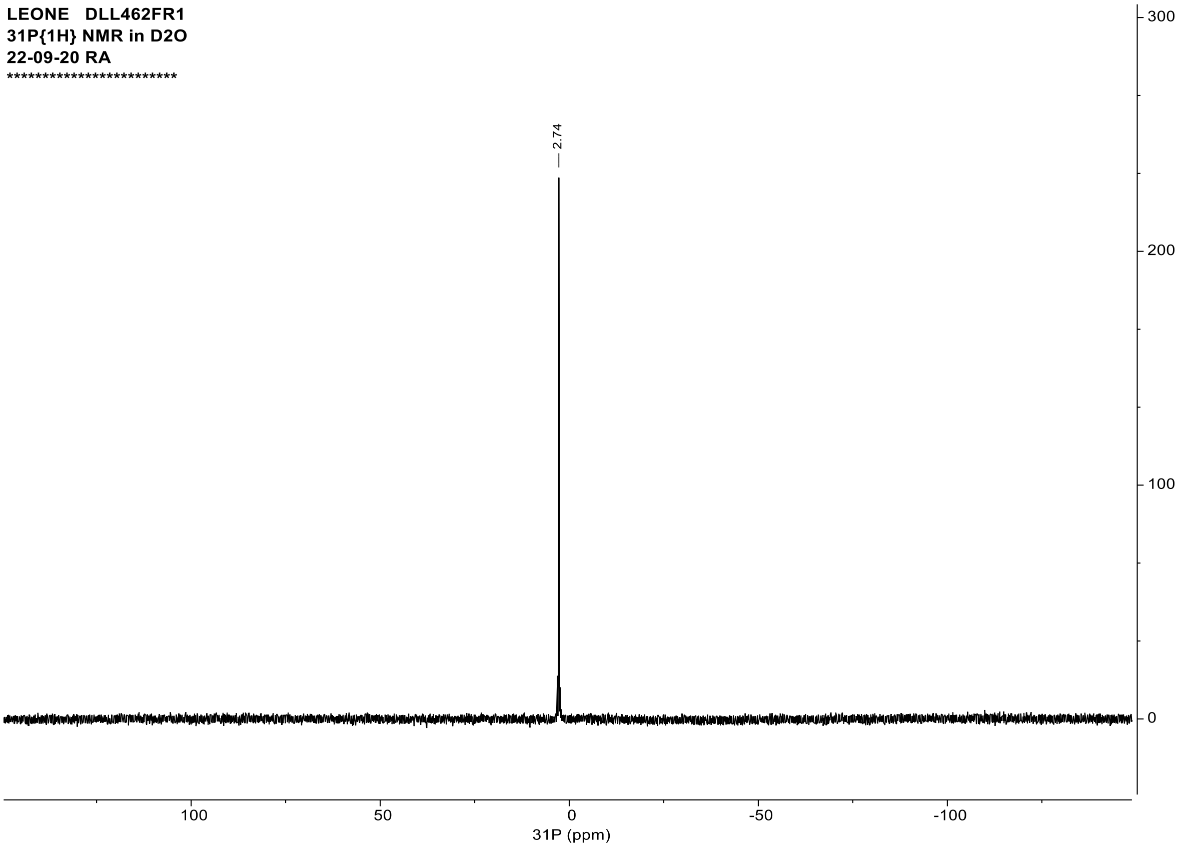
LEONE DLL462FR1
31P{1H} NMR in D2O
22-09-20 RA
****
2.74
31P (ppm)

^{1}H, ^{13}C, ^{31}P Spectra of dT^{PDO} MP

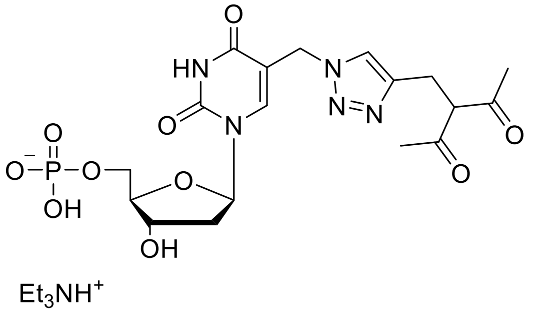

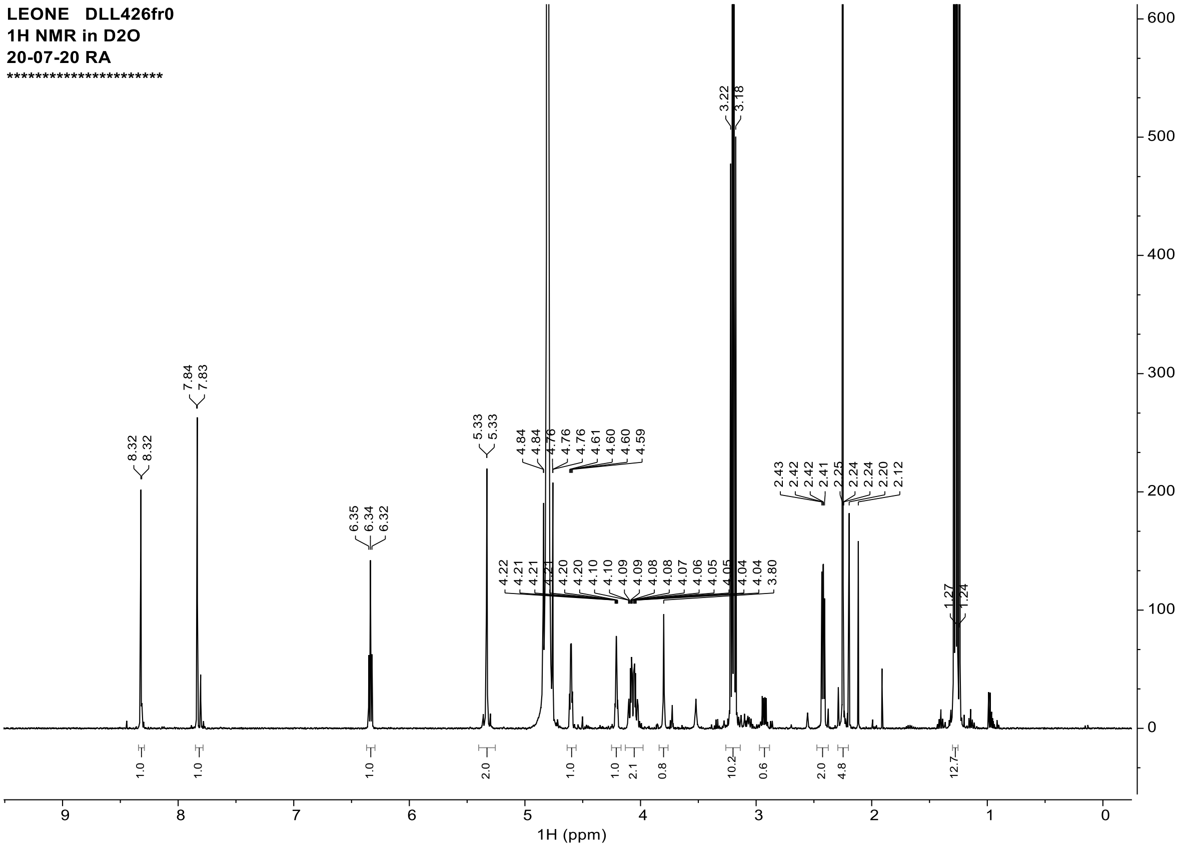

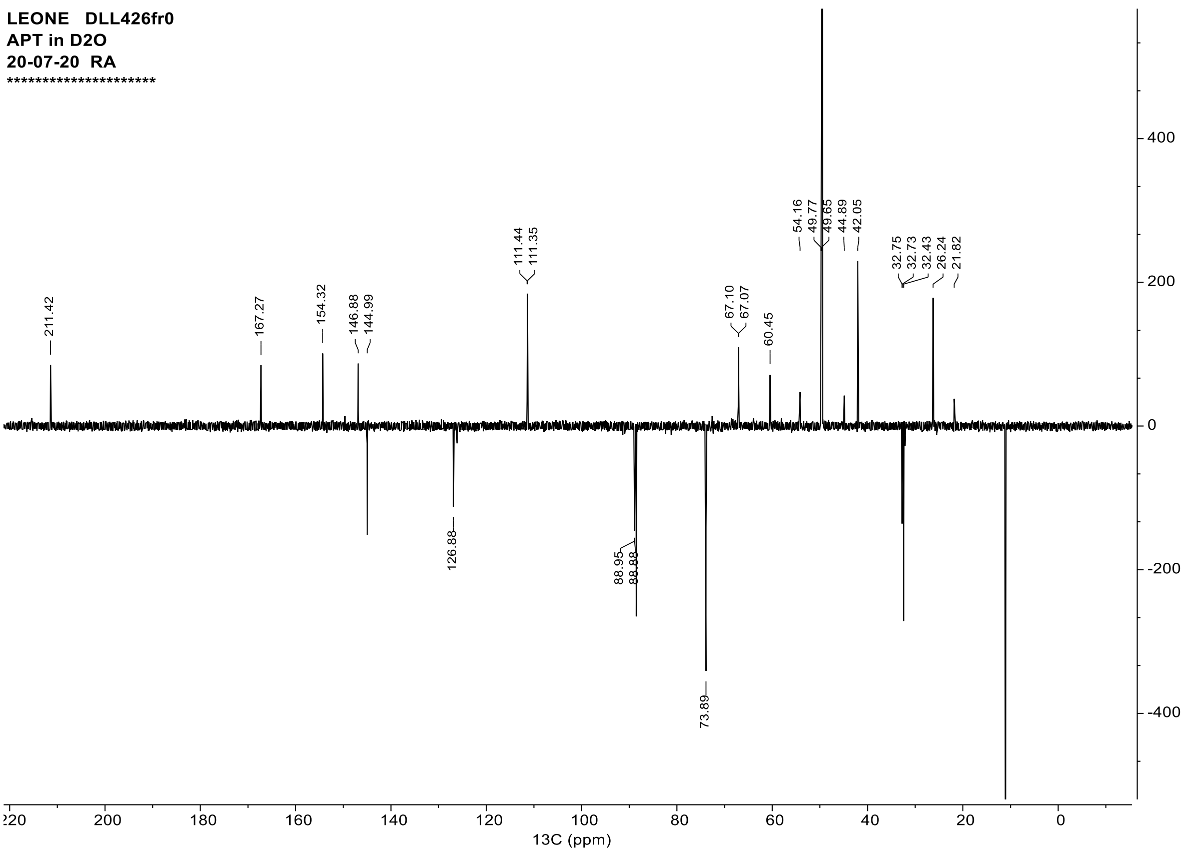
LEONE DLL426fr0
APT in D2O
20-07-20 RA
****
211.42
167.27
154.32
146.88
144.99
111.44
111.35
126.88
88.95
88.88
73.89
67.10
67.07
60.45
54.16
49.77
49.65
44.89
42.05
32.75
32.73
32.43
26.24
21.82
13C (ppm)

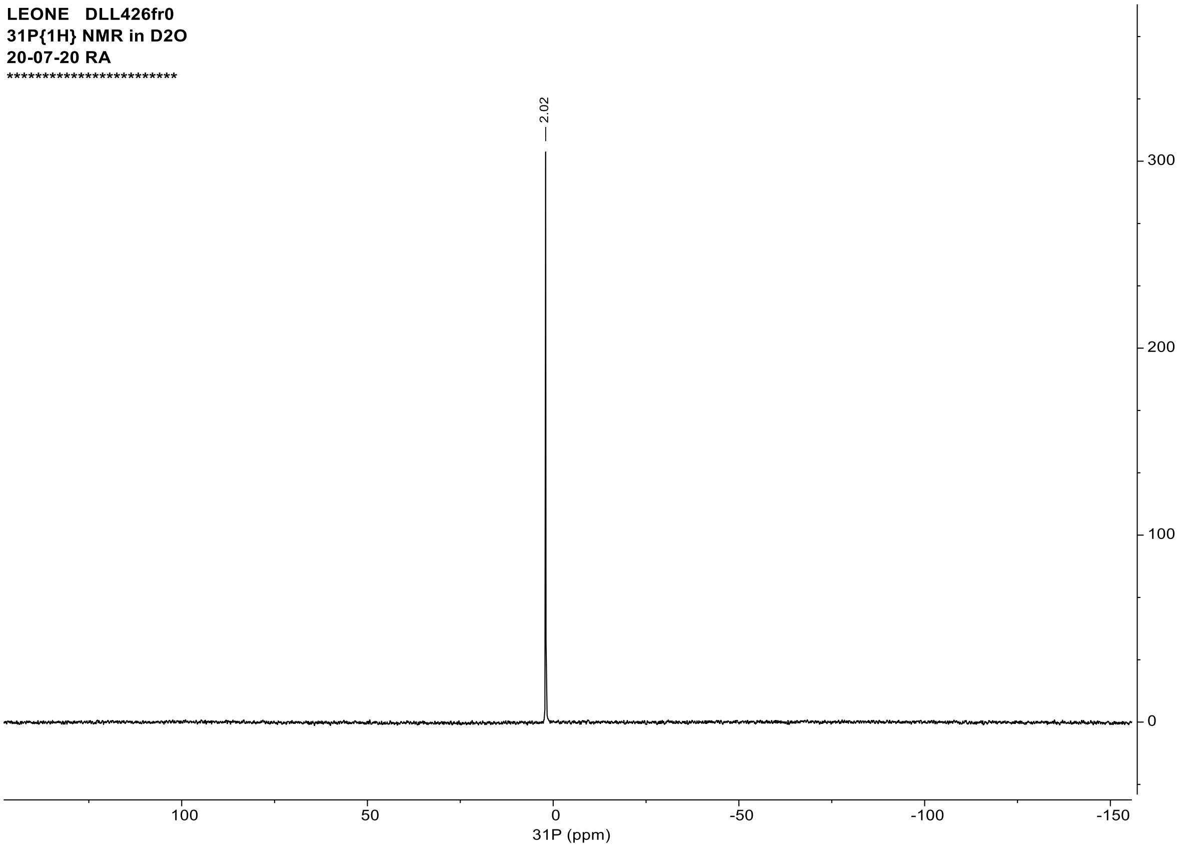
LEONE DLL426fr0
31P{1H} NMR in D2O
20-07-20 RA
****
2.02
31P (ppm)

^{1}H, ^{13}C, ^{31}P Spectra of dT^{HDO} TP

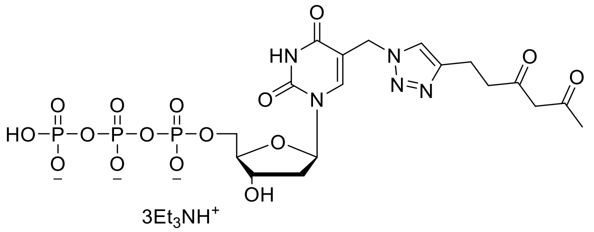

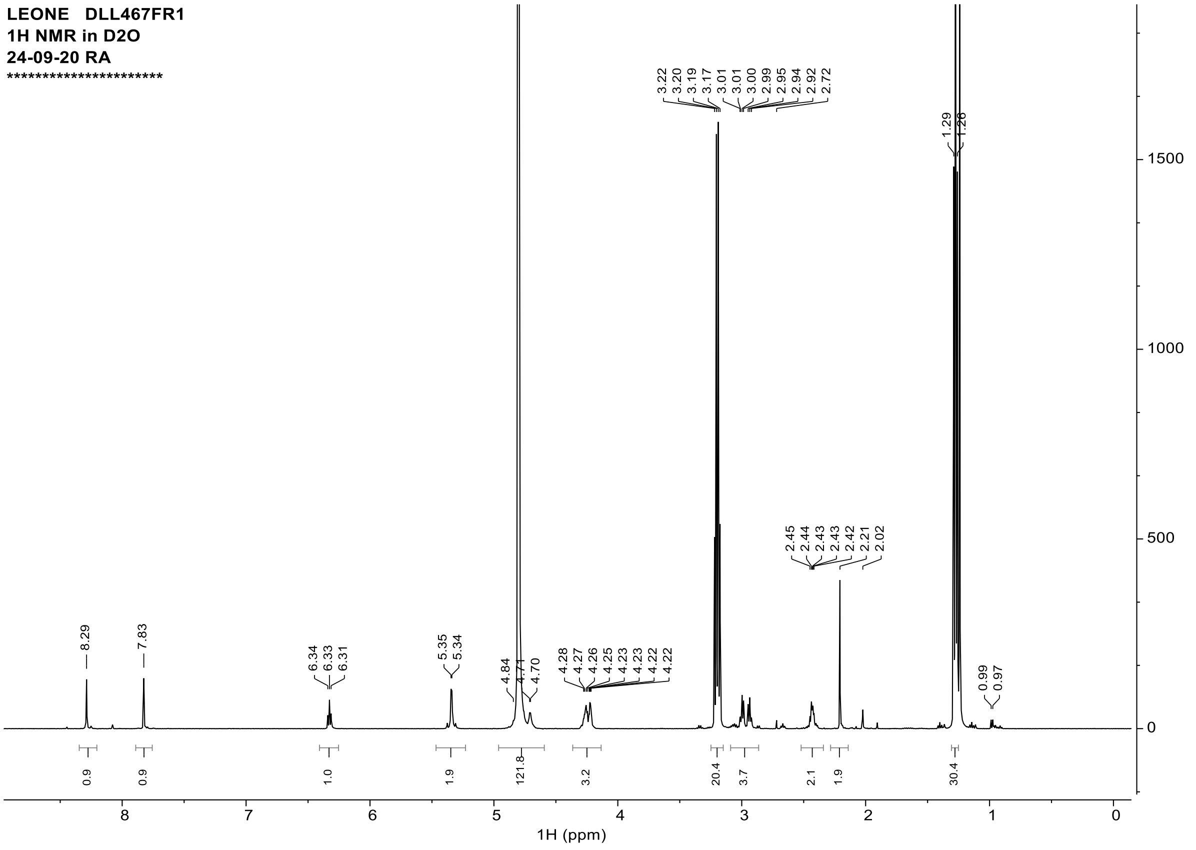

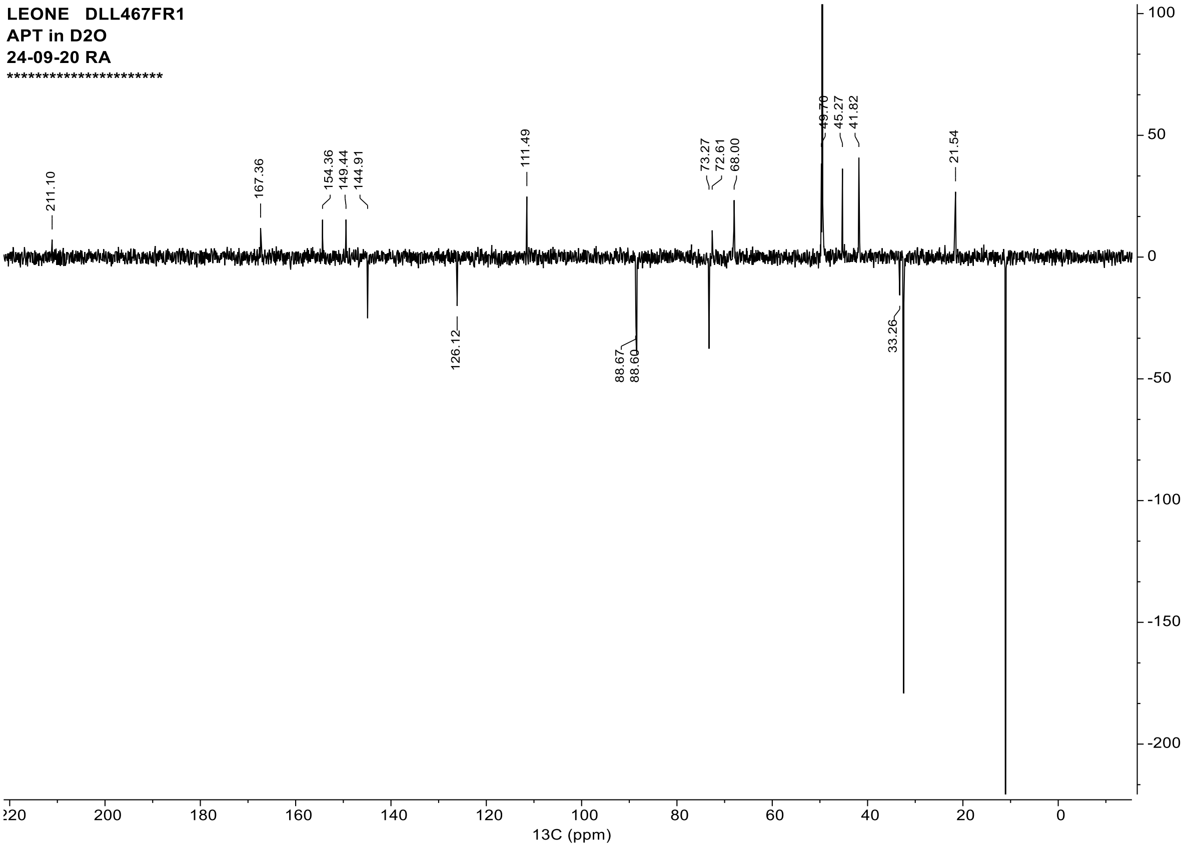
LEONE DLL467FR1
APT in D2O
24-09-20 RA
****
211.10
167.36
154.36
149.44
144.91
126.12
111.49
88.67
88.60
73.27
72.61
68.00
49.70
45.27
41.82
33.26
21.54
13C (ppm)

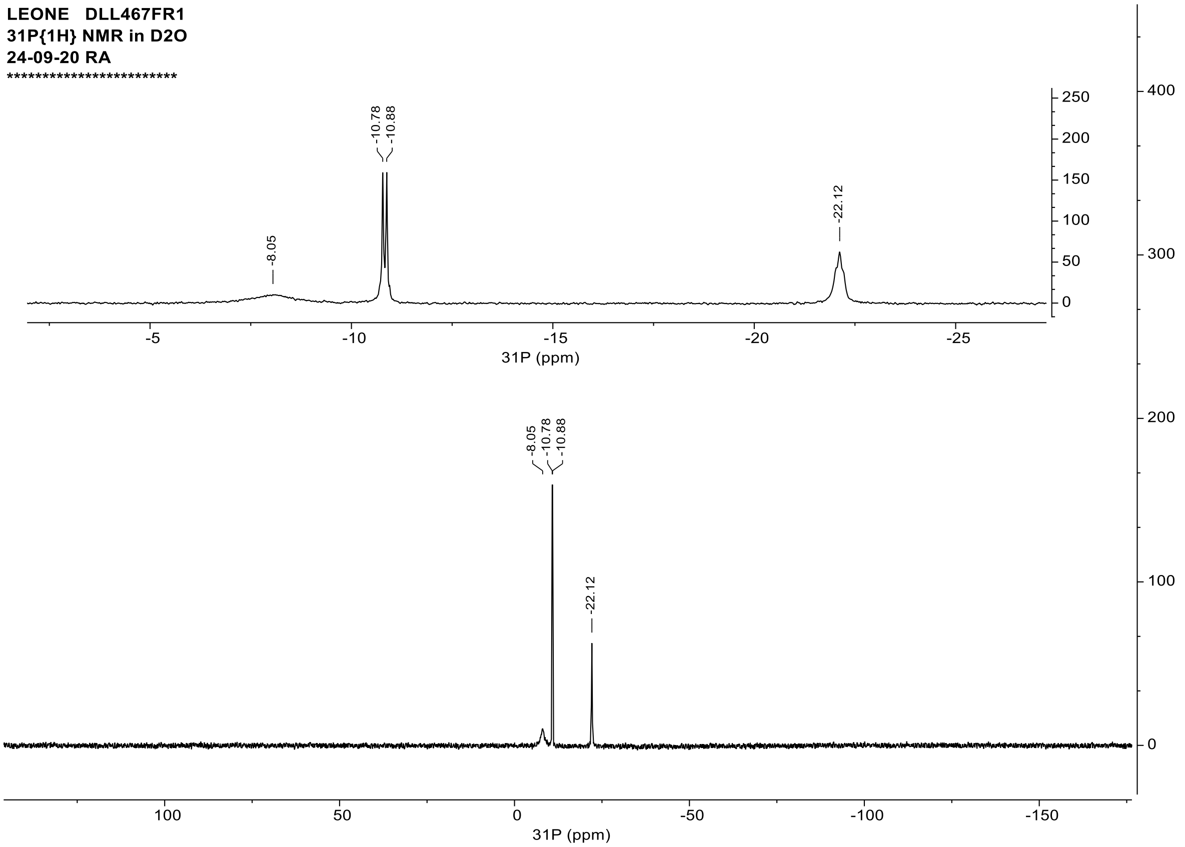
LEONE DLL467FR1
31P{1H} NMR in D2O
24-09-20 RA
****
-8.05
-10.78
-10.88
-22.12
31P (ppm)
31P (ppm)

^{1}H, ^{13}C, ^{31}P Spectra of dT^{PDO} TP

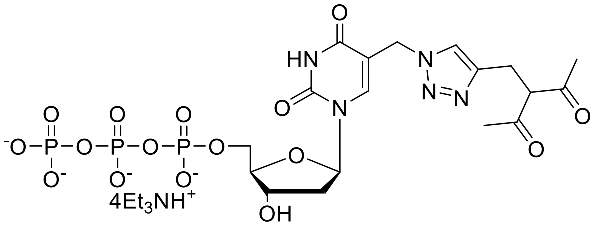

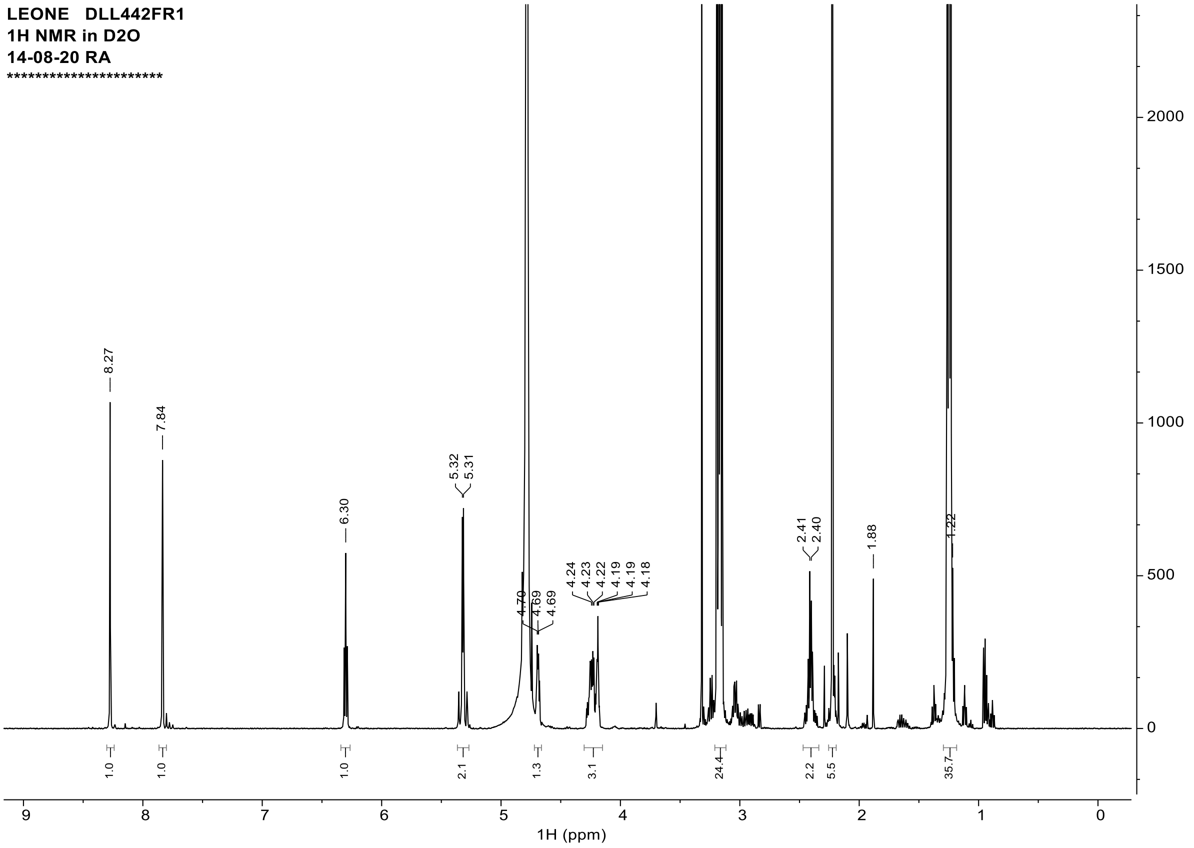

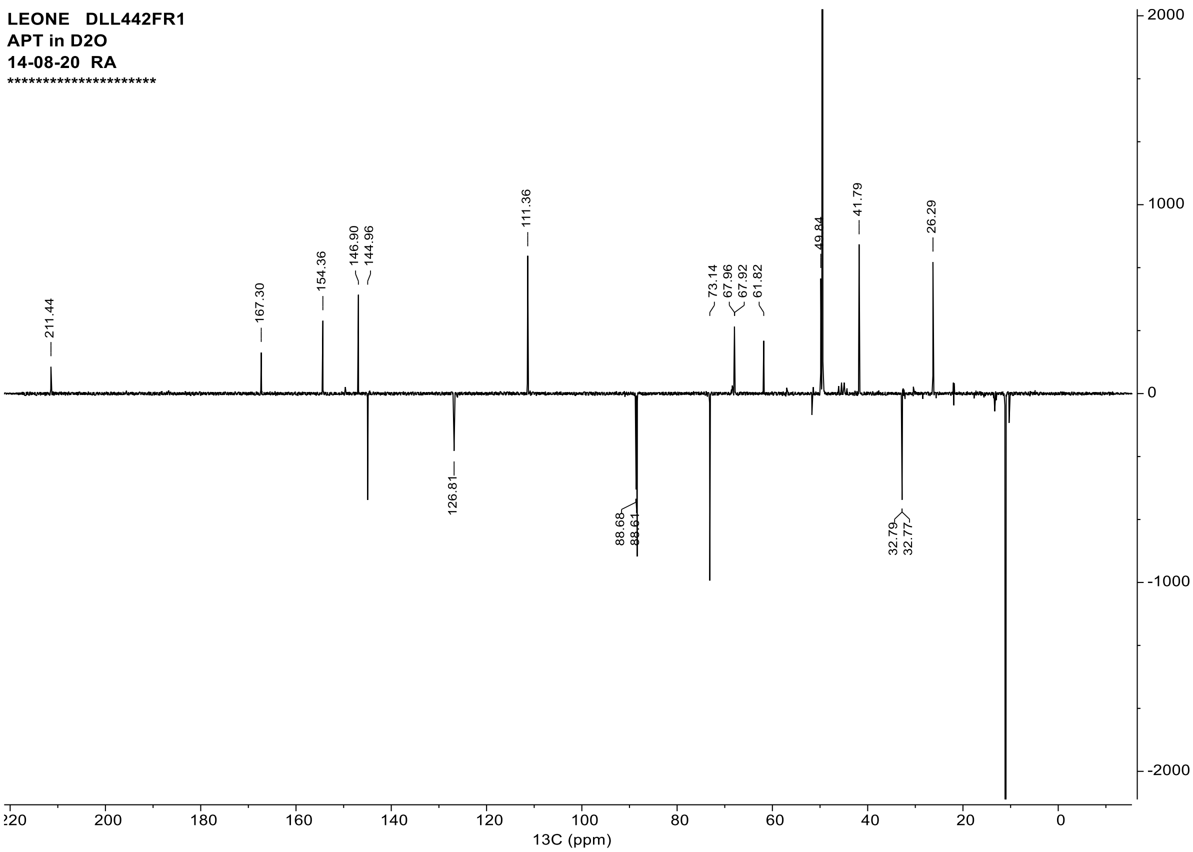
LEONE DLL442FR1
APT in D2O
14-08-20 RA
****
211.44
167.30
154.36
146.90
144.96
126.81
111.36
88.68
88.61
73.14
67.96
67.92
61.82
49.84
41.79
32.79
32.77
26.29
13C (ppm)

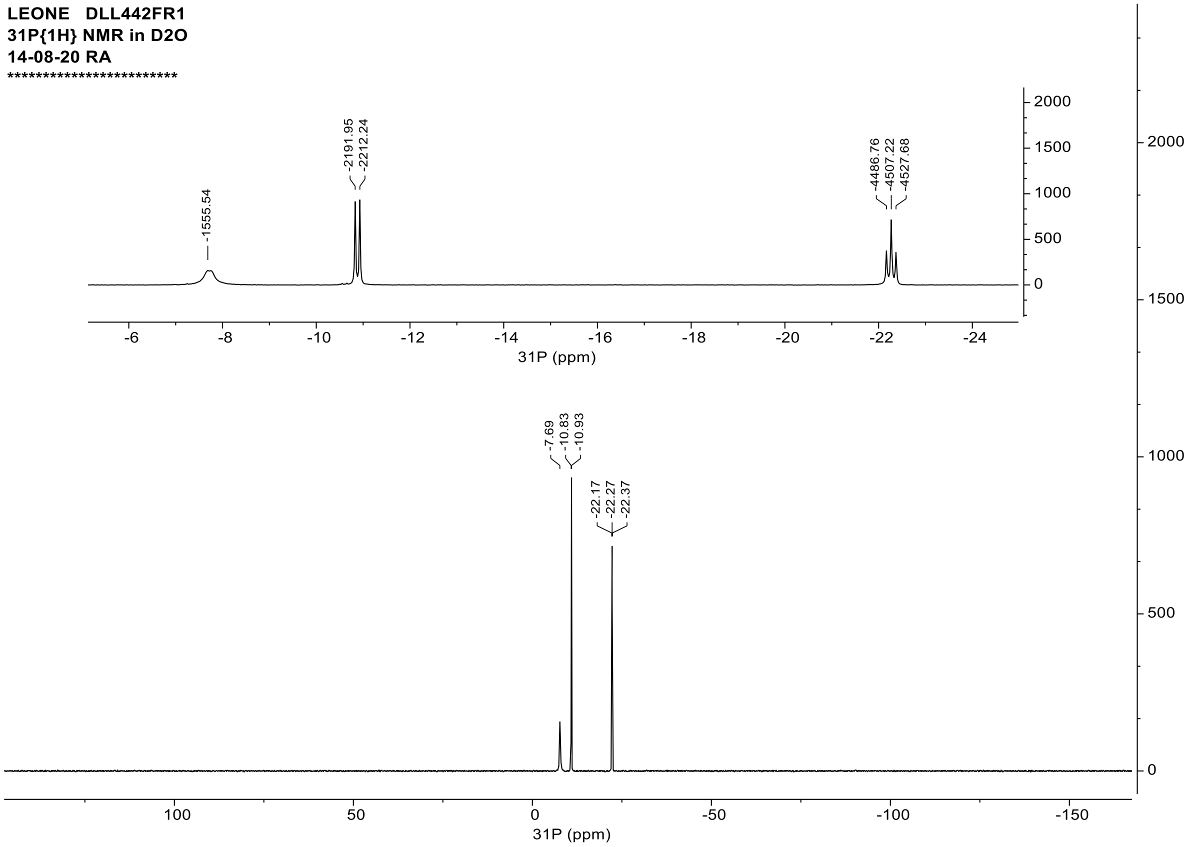
LEONE DLL442FR1
31P{1H} NMR in D2O
14-08-20 RA
****
-1555.54
-2191.95
-2212.24
-4486.76
-4507.22
-4527.68
31P (ppm)
-7.69
-10.83
-10.93
-22.17
-22.27
-22.37
31P (ppm)

^{1}H, ^{13}C, ^{31}P Spectra of dT^{HDOArg}MP

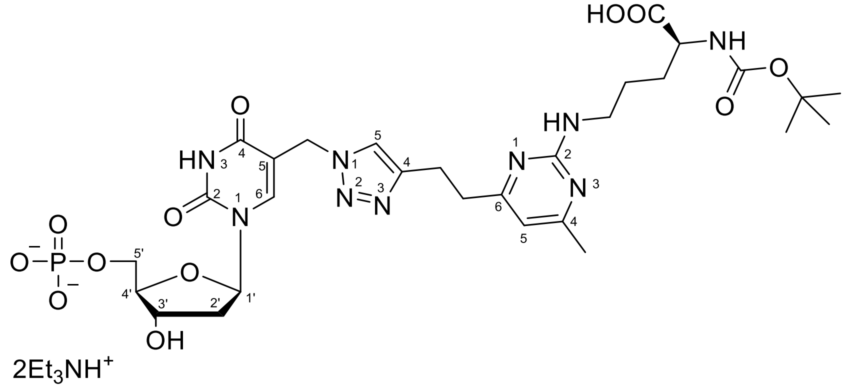

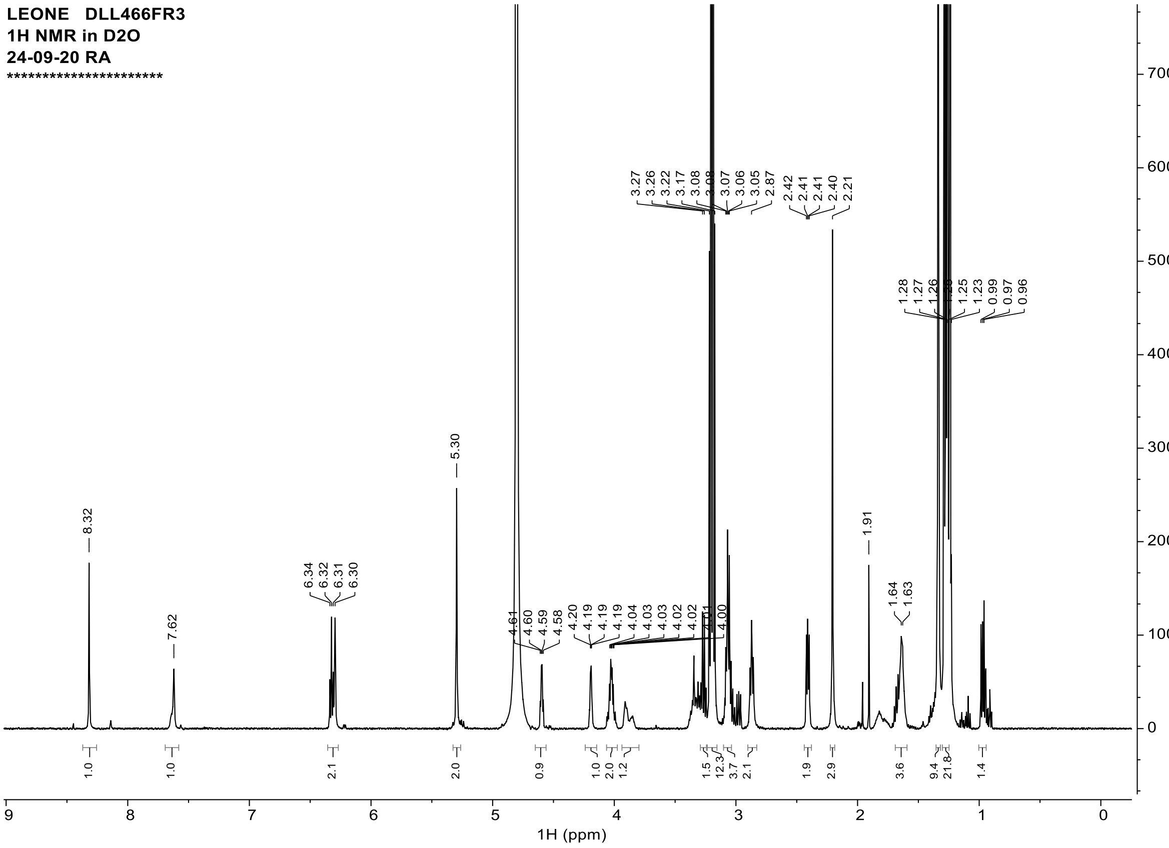

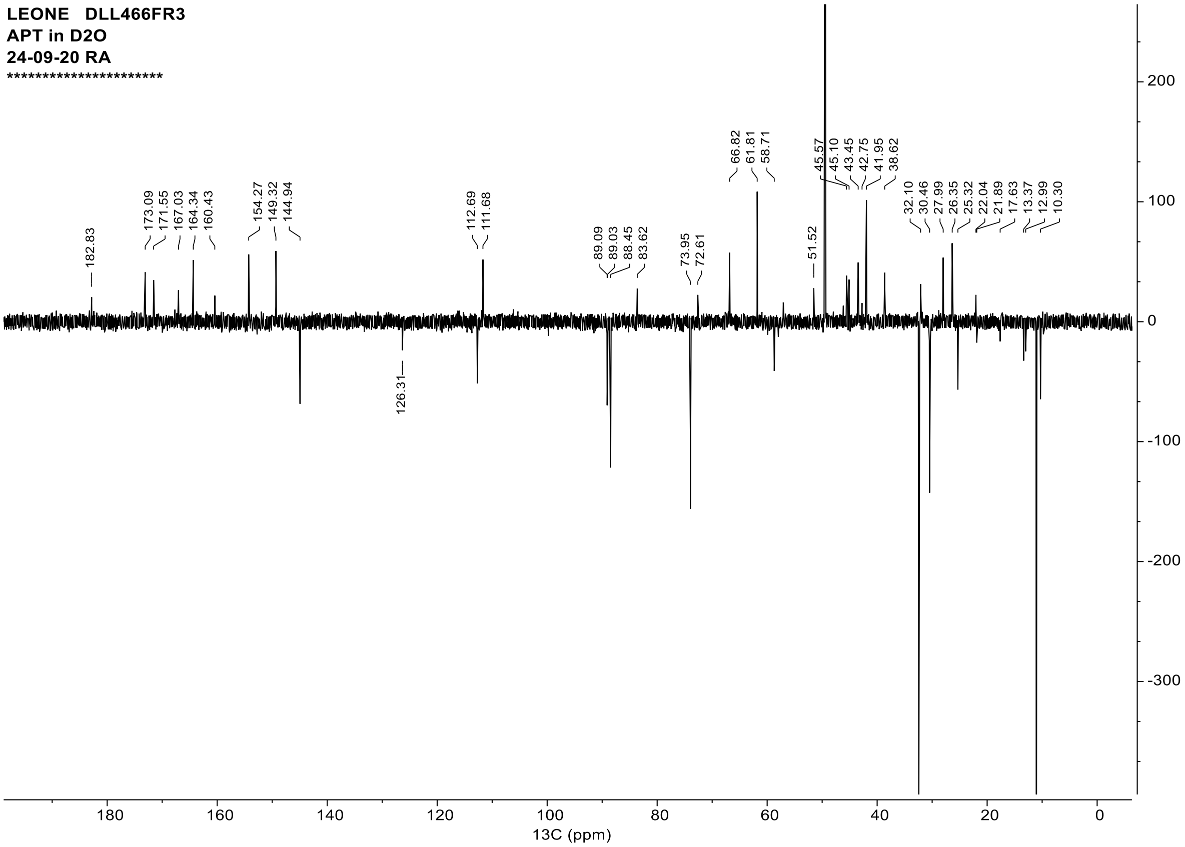
LEONE DLL466FR3
APT in D2O
24-09-20 RA
****
182.83
173.09
171.55
167.03
164.34
160.43
154.27
149.32
144.94
126.31
112.69
111.68
89.09
89.03
88.45
83.62
73.95
72.61
66.82
61.81
58.71
51.52
45.57
45.10
43.45
42.75
41.95
38.62
32.10
30.46
27.99
26.35
25.32
22.04
21.89
17.63
13.37
12.99
10.30
13C (ppm)

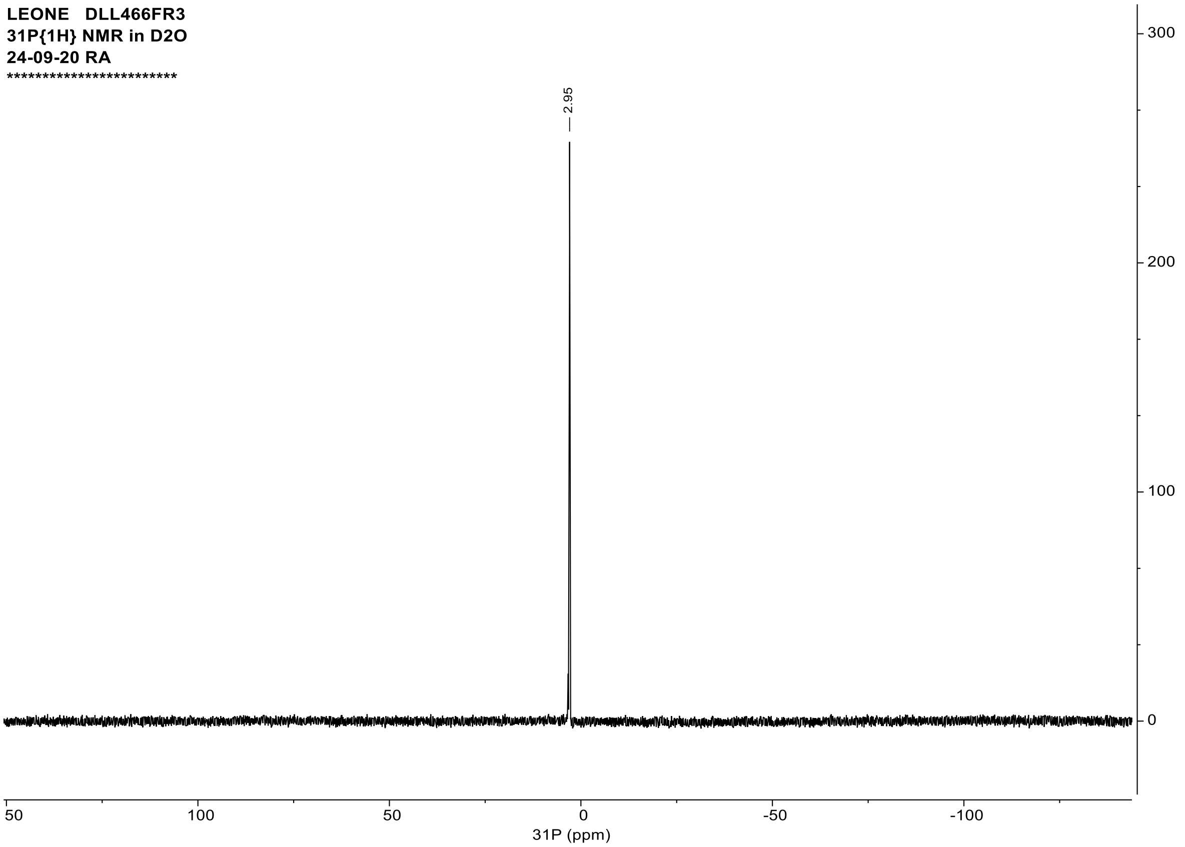
LEONE DLL466FR3
31P{1H} NMR in D2O
24-09-20 RA
****
2.95
31P (ppm)

^{1}H, ^{13}C, [31]P Spectra of dT^{HDOARA} MP

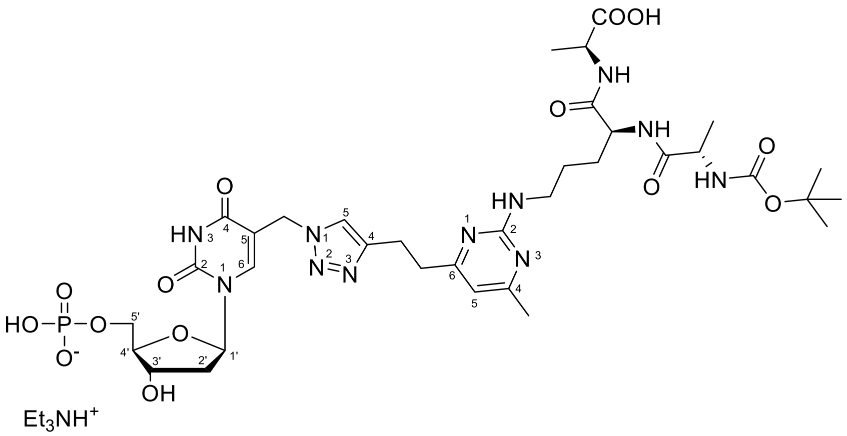

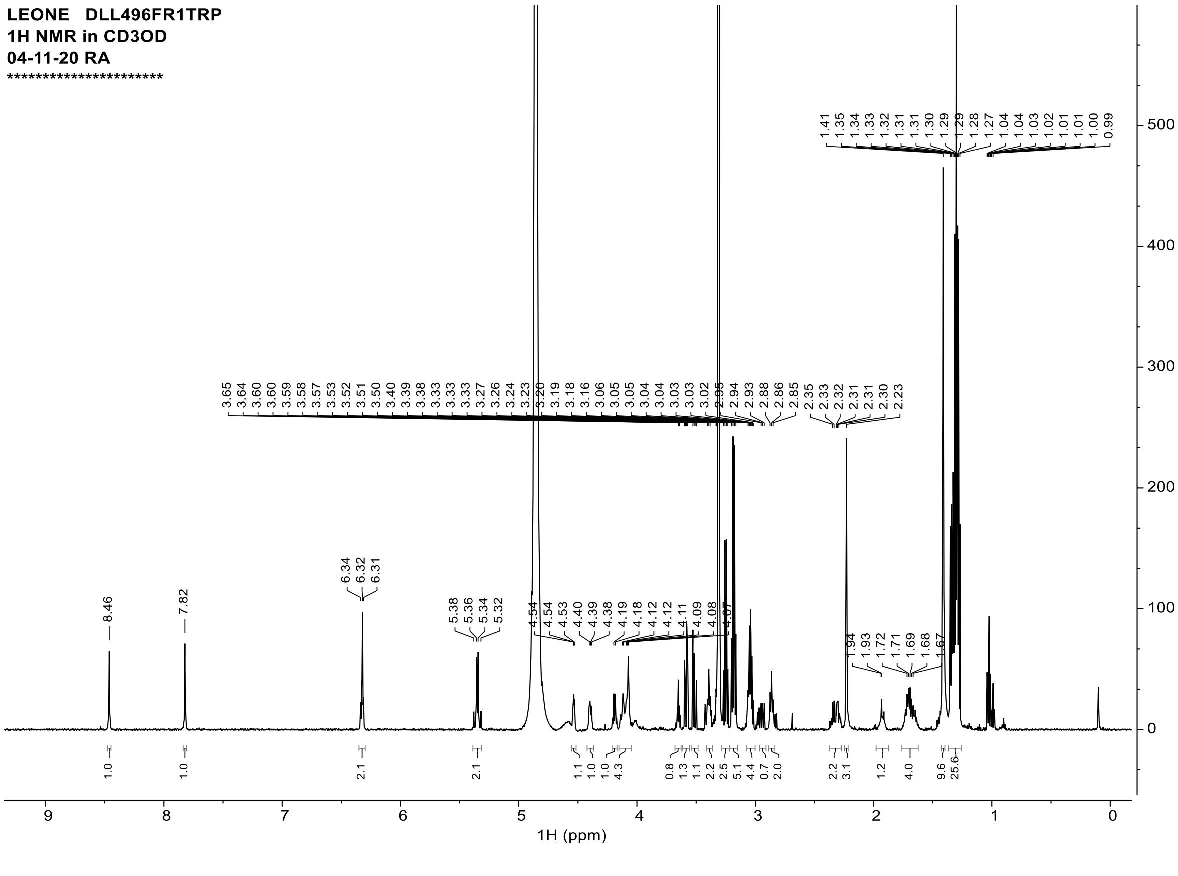

LEONE DLL496FR1TRP
APT in CD3OD
04-11-20 RA
****

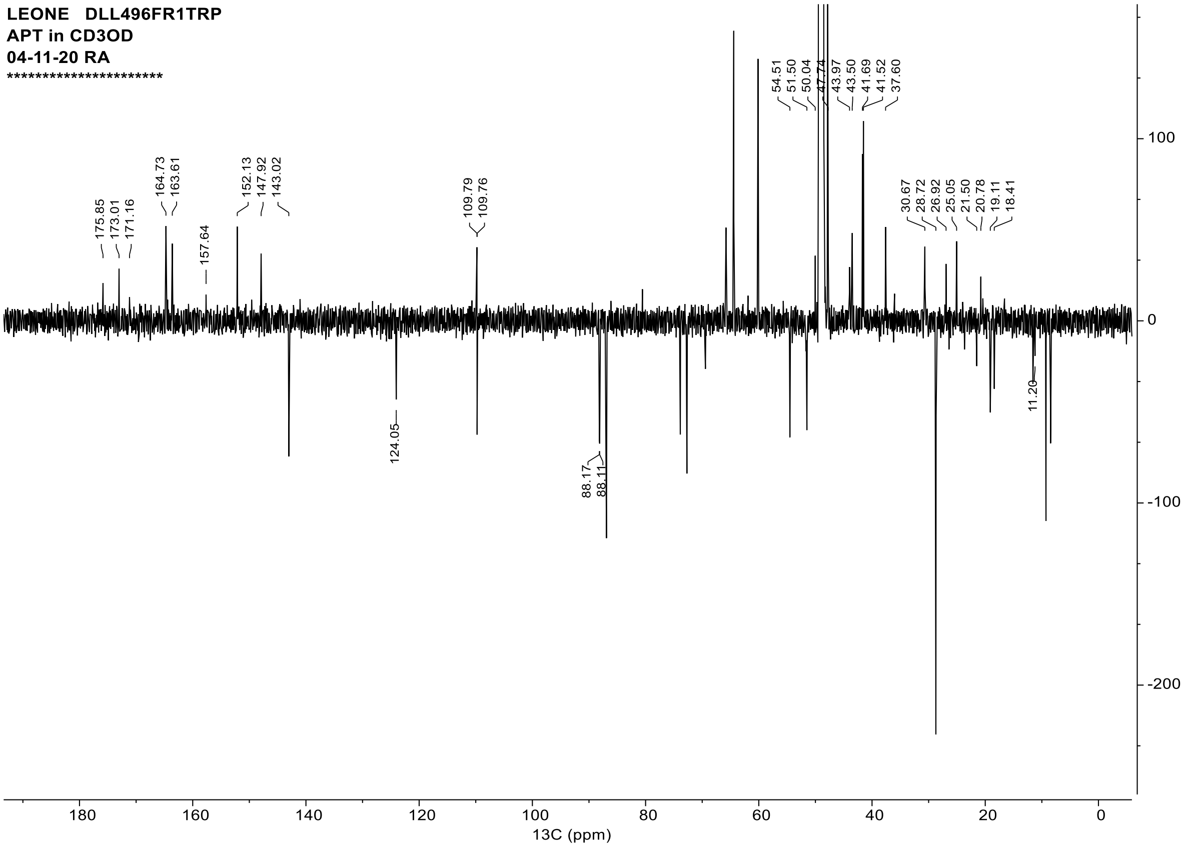

LEONE DLL496FR1TRP
31P{1H} NMR in CD3OD
05-11-20 RA
****

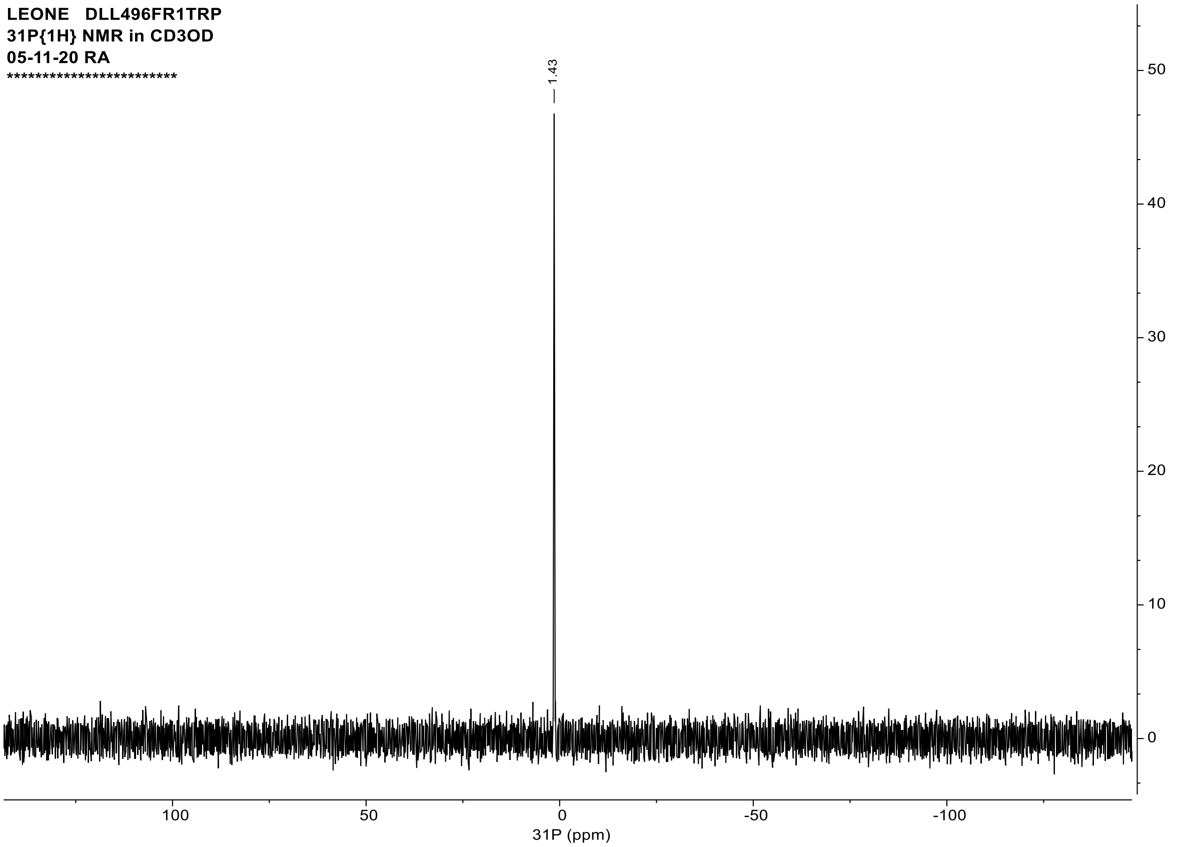

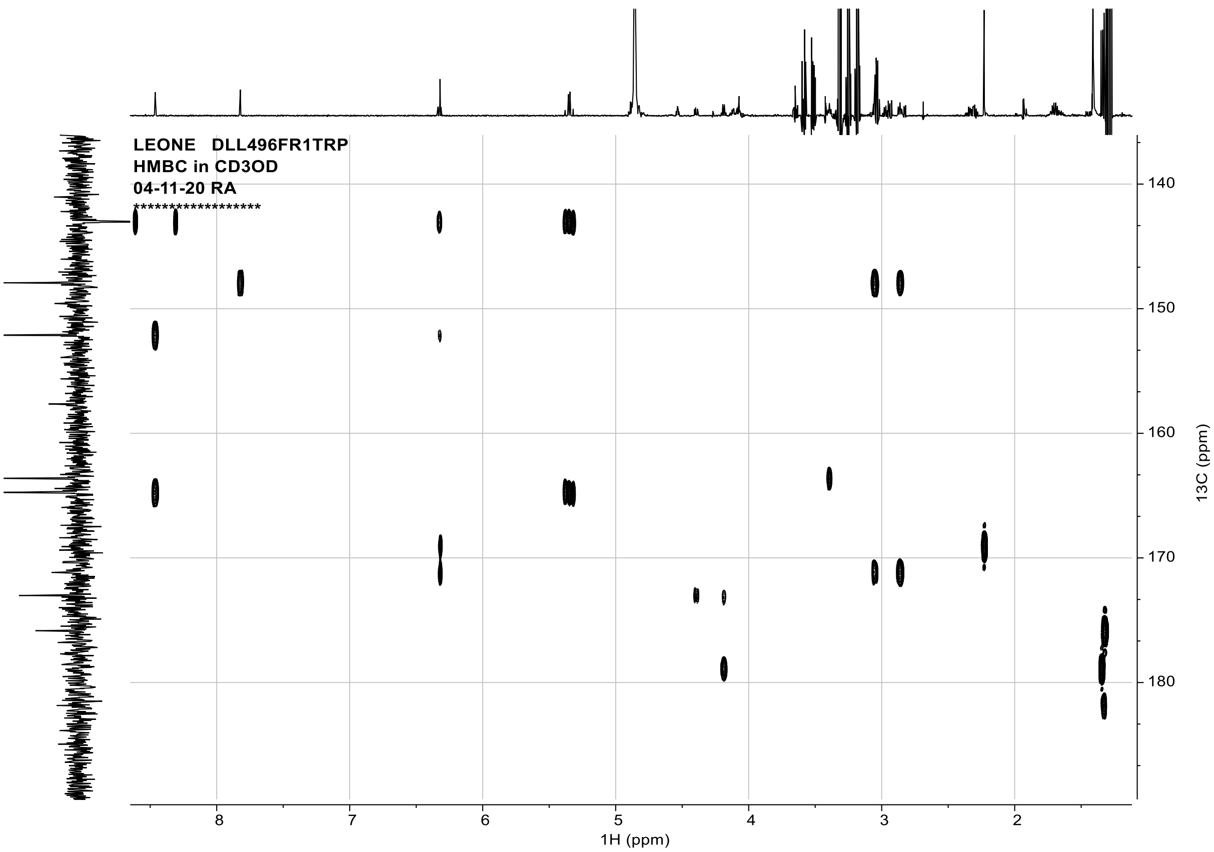

^{1}H, ^{13}C, ^{31}P Spectra of dT^{HDOAGA}MP

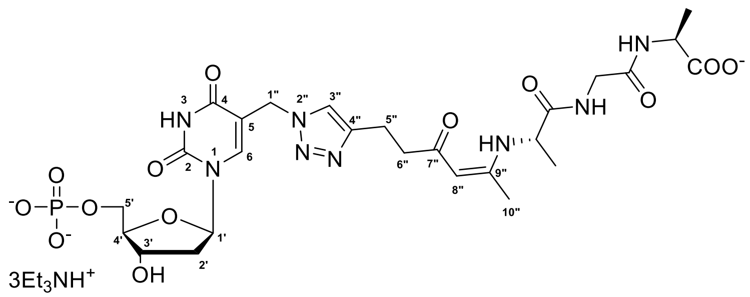

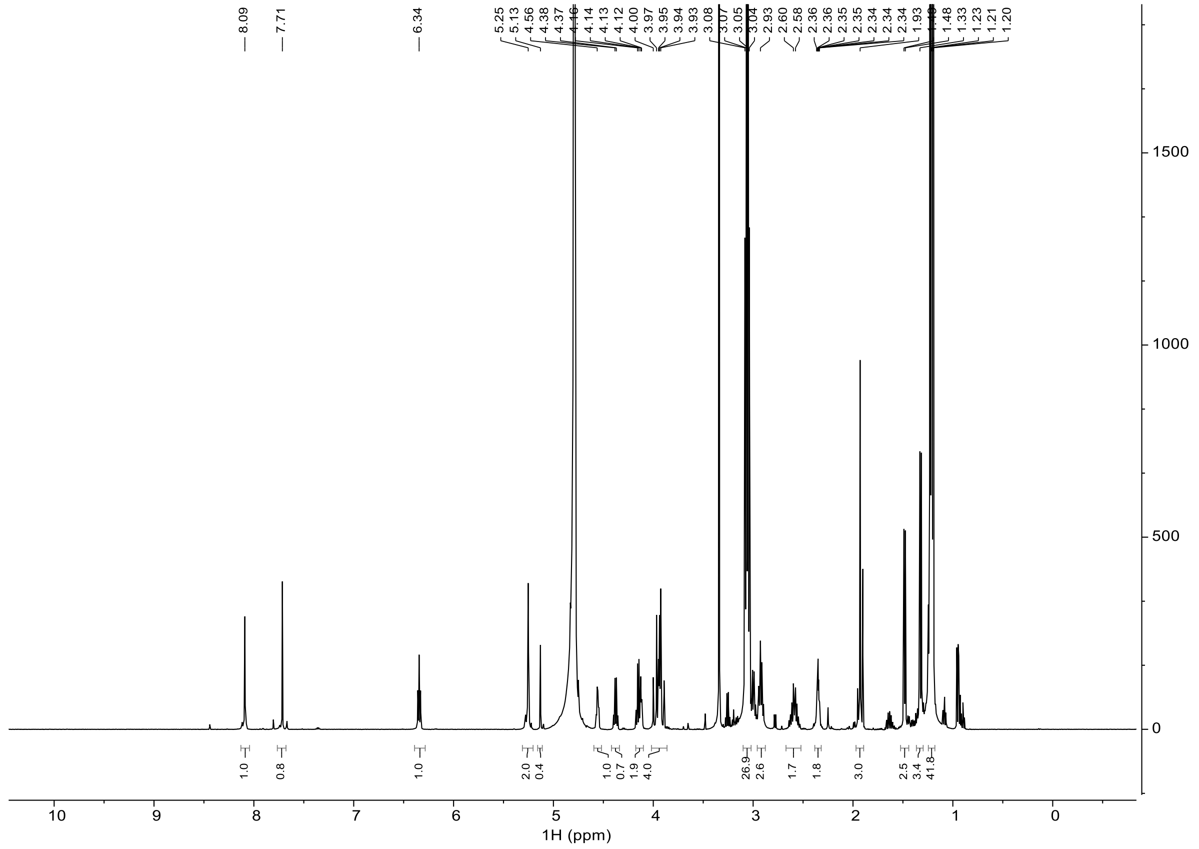

8.09
7.71
6.34
5.25
5.13
4.56
4.38
4.37
4.16
4.14
4.13
4.12
4.00
3.97
3.95
3.94
3.93
3.08
3.07
3.05
3.04
2.93
2.60
2.58
2.36
2.36
2.35
2.35
2.34
2.34
2.34
2.34
1.93
1.48
1.48
1.33
1.23
1.21
1.20
1500
1000
500
0
1.0
0.8
1.0
2.0
0.4
1.0
0.7
1.9
4.0
26.9
2.6
1.7
1.8
3.0
2.5
3.4
41.8
10
9
8
7
6
5
4
3
2
1
0
1H (ppm)

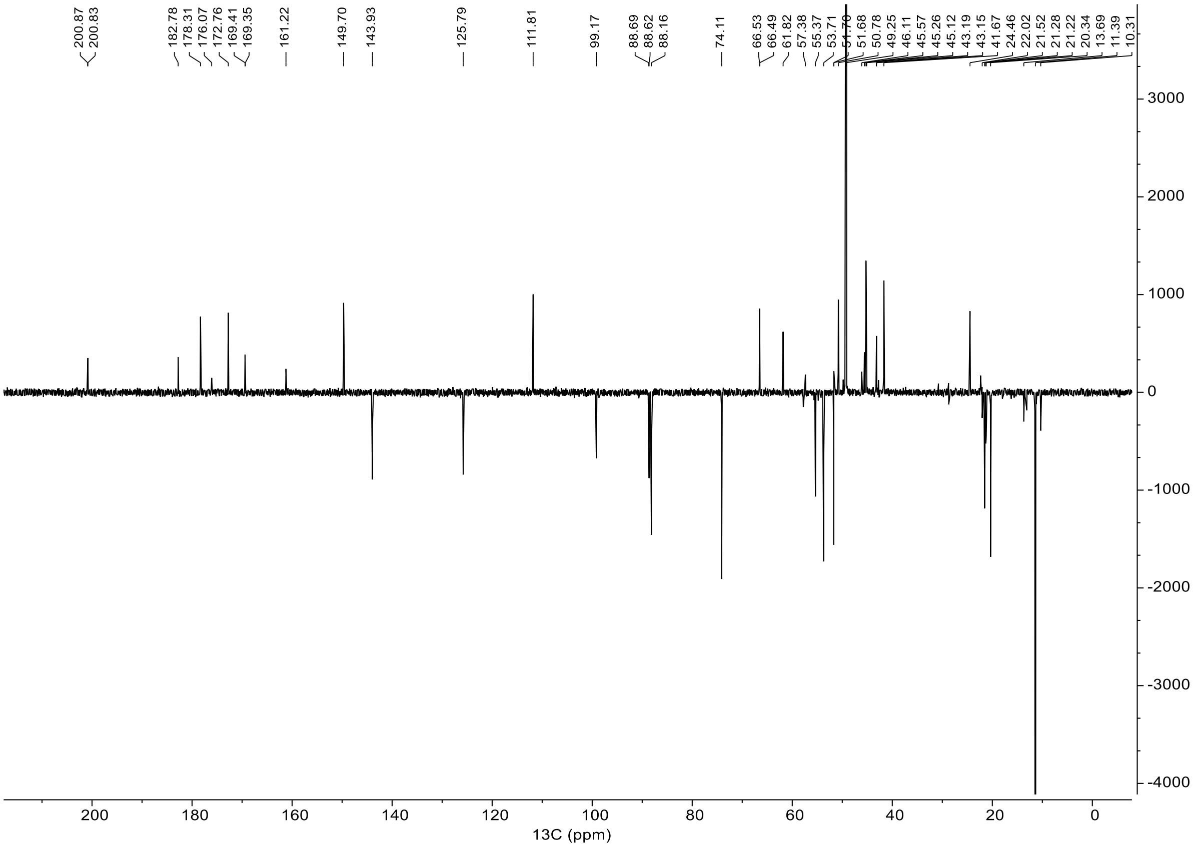
200.87
200.83
182.78
178.31
176.07
172.76
169.41
169.35
161.22
149.70
143.93
125.79
111.81
99.17
88.69
88.62
88.16
74.11
66.53
66.49
61.82
57.38
55.37
53.71
51.70
51.68
50.78
49.25
46.11
45.57
45.26
45.12
43.19
43.15
41.67
24.46
22.02
21.52
21.28
21.22
20.34
13.69
11.39
10.31
13C (ppm)

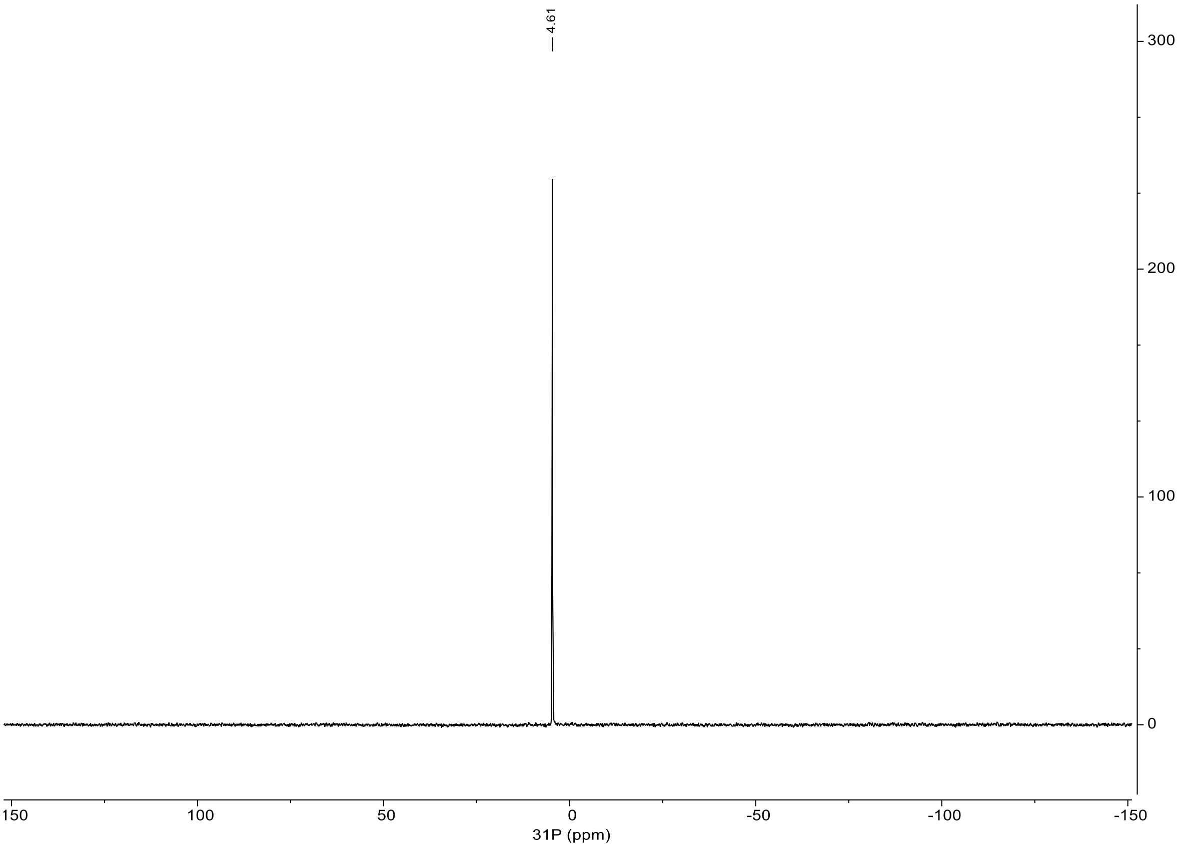
4.61
31P (ppm)

^{1}H, ^{13}C, ^{31}P Spectra of dT^{HDOAKA}MP

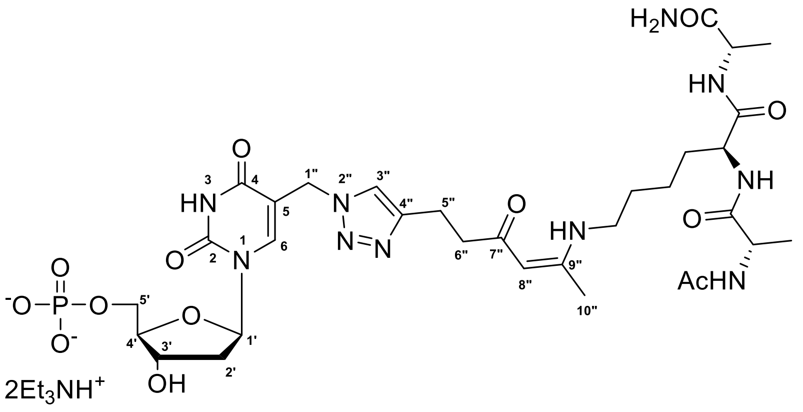

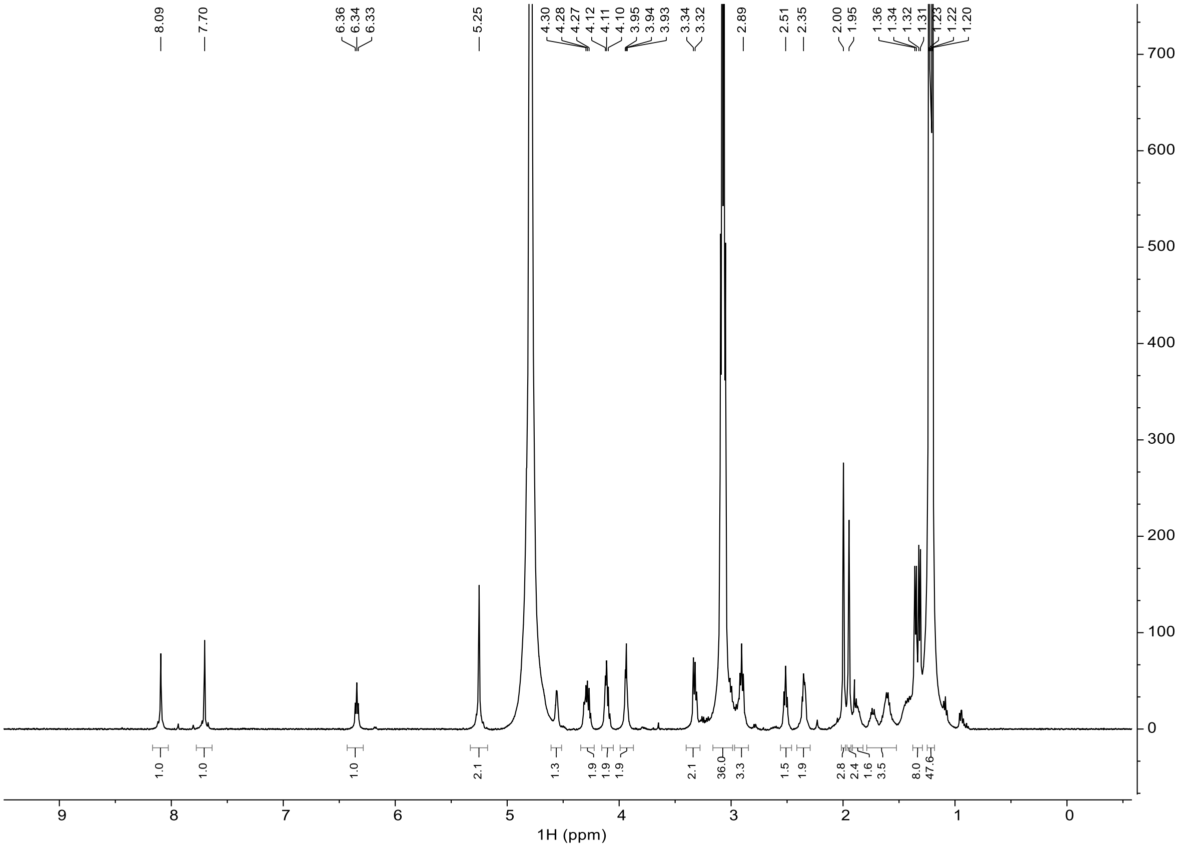

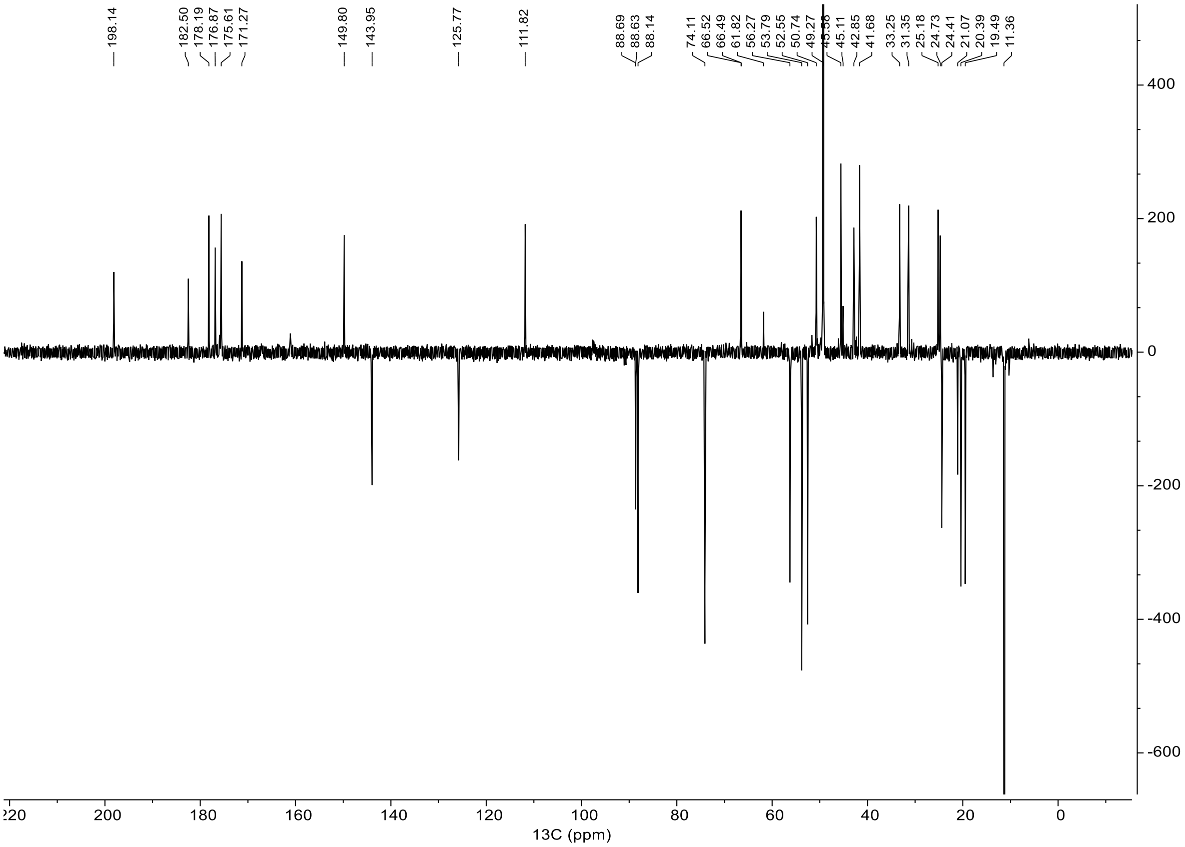
198.14
182.50
178.19
176.87
175.61
171.27
149.80
143.95
125.77
111.82
88.69
88.63
88.14
74.11
66.52
66.49
61.82
56.27
53.79
52.55
50.74
49.27
45.11
42.85
41.68
33.25
31.35
25.18
24.73
24.41
21.07
20.39
19.49
11.36
13C (ppm)

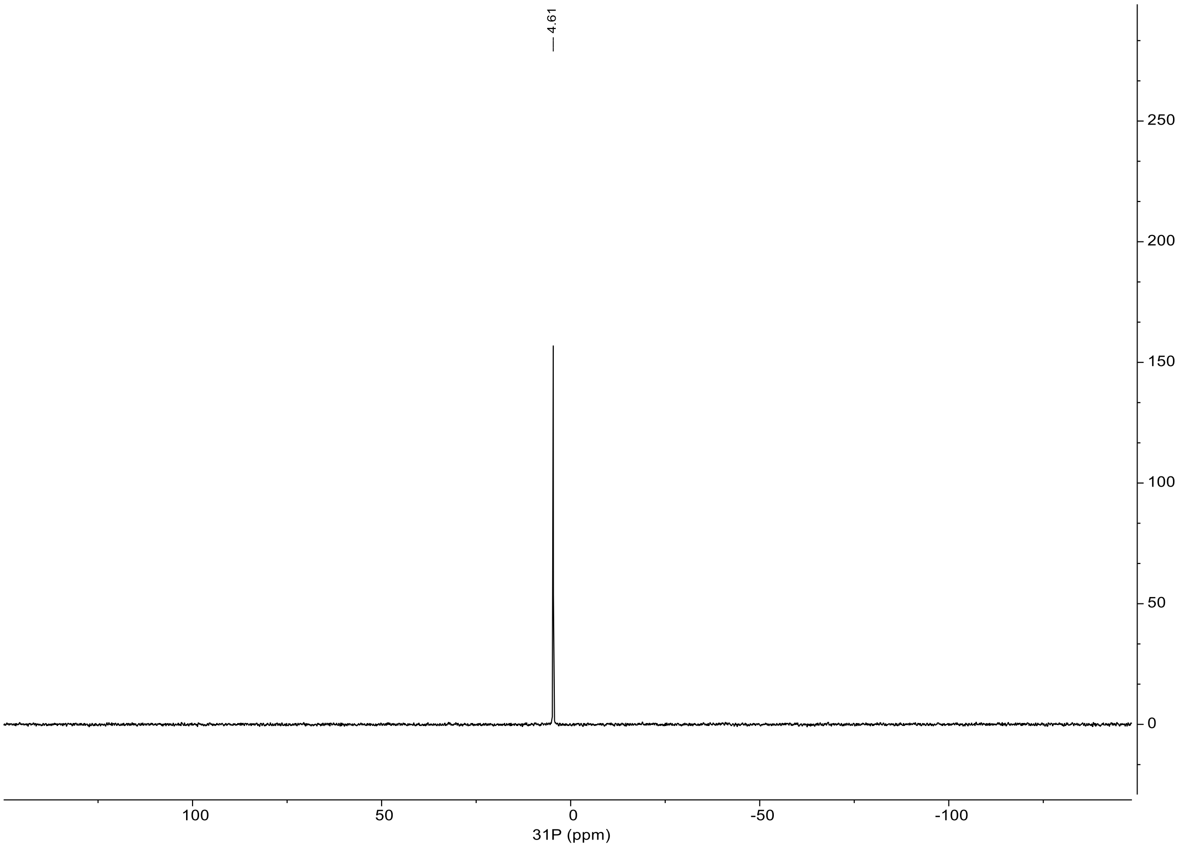
4.61
31P (ppm)

^{1}H, ^{13}C, ^{31}P Spectra of dT^{HDOARA}MP

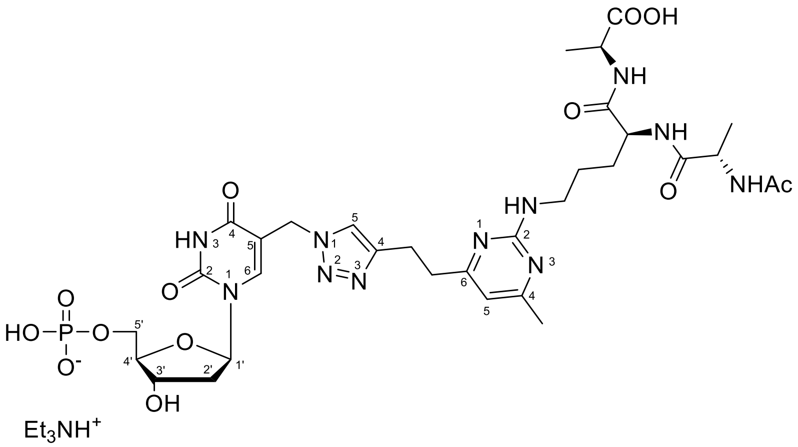

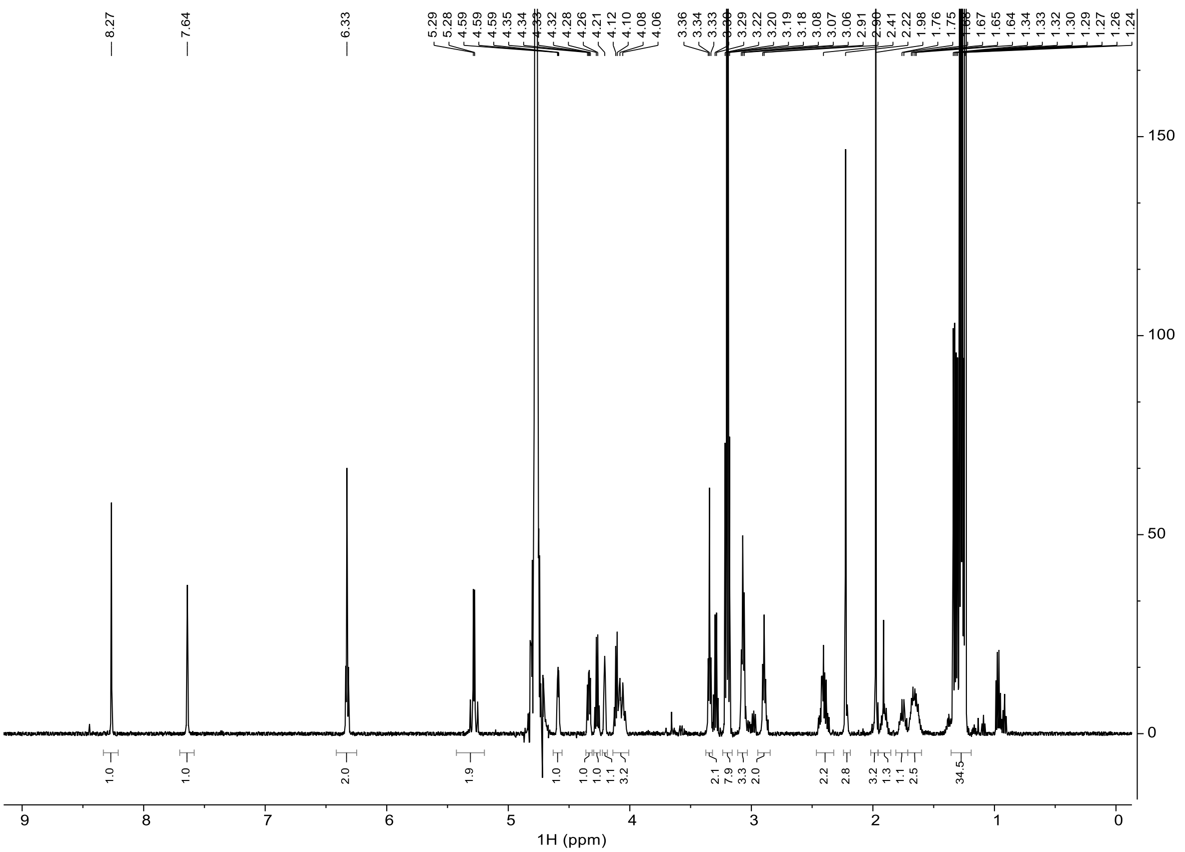

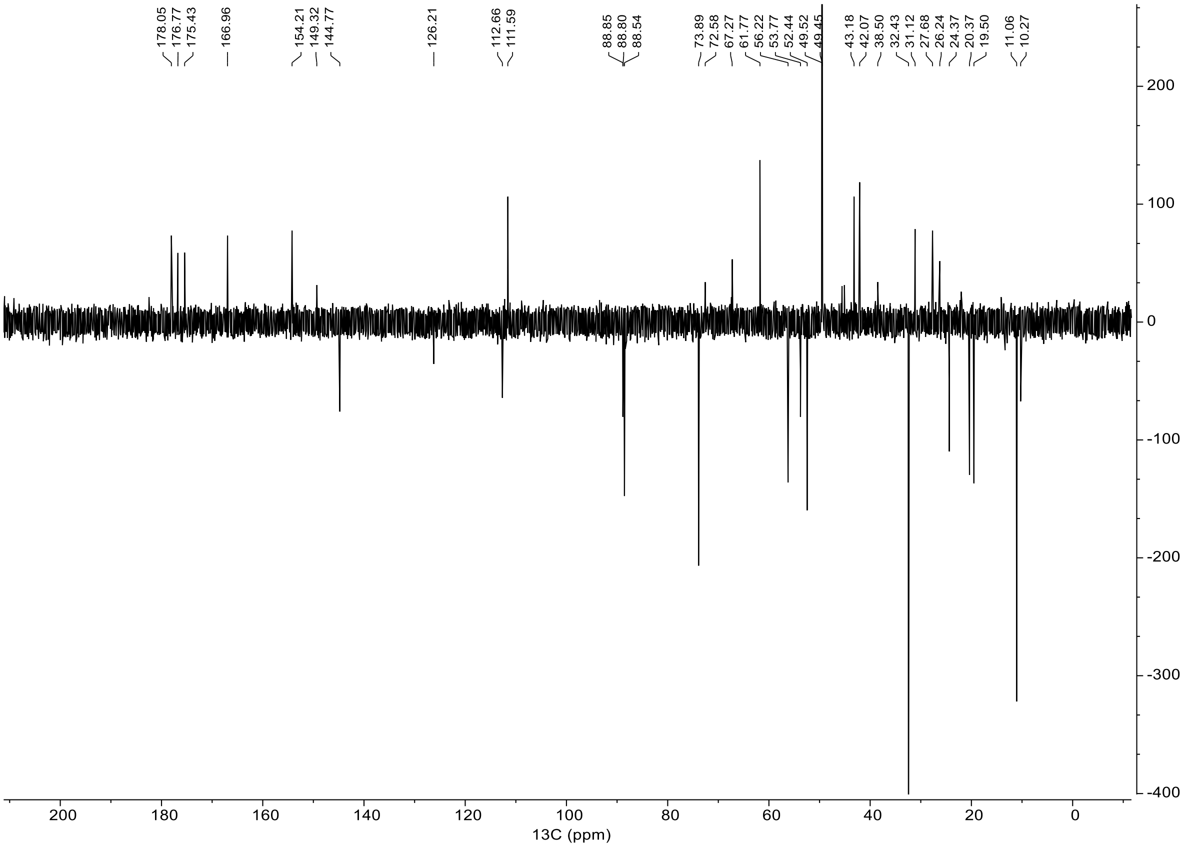
178.05
176.77
175.43
166.96
154.21
149.32
144.77
126.21
112.66
111.59
88.85
88.80
88.54
73.89
72.58
67.27
61.77
56.22
53.77
52.44
49.52
49.45
43.18
42.07
38.50
32.43
31.12
27.68
26.24
24.37
20.37
19.50
11.06
10.27
13C (ppm)

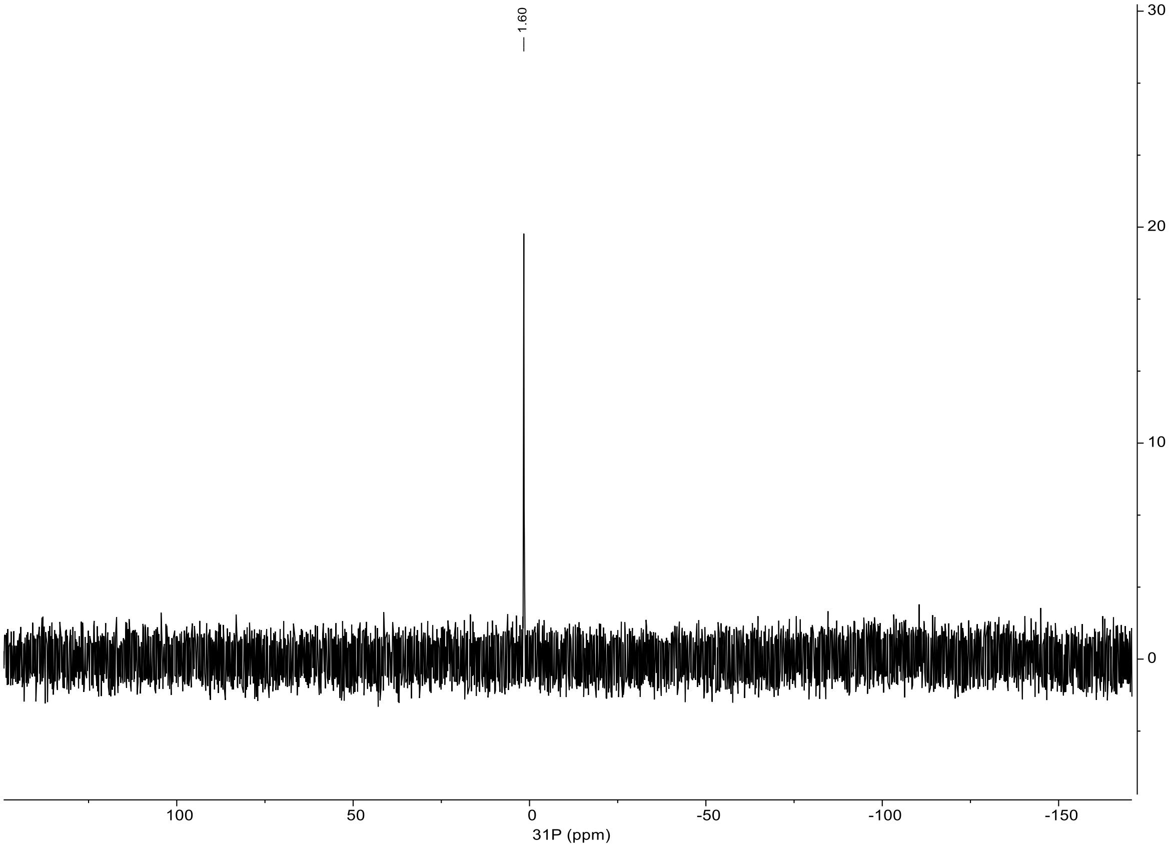
1.60
31P (ppm)

6. References

1. X. Ren, A. El-Sagheer, T. Brown, *Analyst.* **2015,** *140* (8), 2671-2678.

2. A.B. Neef , N. W. Luedtke, *ChemBioChem.* **2014**, *15* (6), 789-793.

3 C. Subin, Srinivasulu V., S. Ha, Park C-M. *Chemical Communications*. **2017**, *53* (24), 3481 – 3484.

4. I. Misztalewska, A.Z. Wilczewska, O. Wojtasik, K. H. Markiewicz, P. Kuchlewski Majcher A.M. . *RSC Advances* **2015,** *5*, 100281..

5. I. Ivancová, R. Pohl, M. Hubálek, M. Hocek, *Angew. Chem. Int*. **2019**, *58*, 13345-13348.

6. M. V. Monakhova, E. A. Kubareva, E. A. Romanova, A. S. Semkina, D. S. Naberezhnov, D. N. Rao, T. S. Zatsepin, T. S. Oretskaya, *Russ. J. Bioorg. Chem.* **2019**, *45*, 144–154.

7.A.G. Ferrige, M.J. Seddon, B.N. Green, S.A. Jarvis, J. *Rapid Commun. Mass Spectrom*. **1992**, 6, 707-711.
